# Telluride-Based Pillar[5]arene: A Recyclable Catalyst for Alkylation Reactions in Aqueous Solution

**DOI:** 10.1021/acs.joc.4c00997

**Published:** 2024-09-05

**Authors:** Patrick C. Nobre, Pâmella Cordeiro, Ingrid C. Chipoline, Victor Menezes, Kaila V. S. Santos, Alix Y. Bastidas Ángel, Eduardo E. Alberto, Vanessa Nascimento

**Affiliations:** † SupraSelen Laboratory, Department of Organic Chemistry, Universidade Federal Fluminense, Campus do Valonguinho, Niterói, Rio de Janeiro 24020-141, Brazil; ‡ Departamento de Química, Universidade Federal de Minas Gerais−UFMG, Belo Horizonte, Minas Gerais 31270-901, Brazil

## Abstract

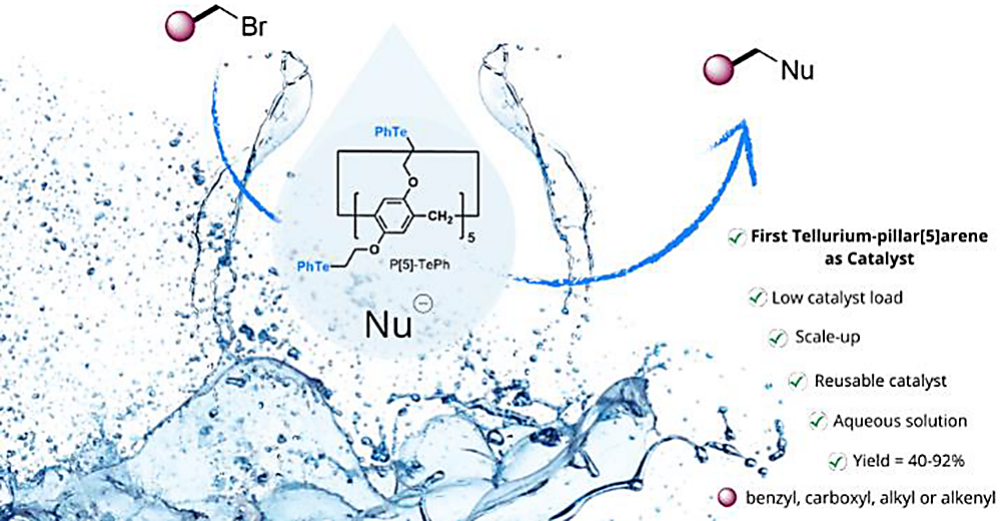

The syntheses of previously unknown sulfide- and telluride-pillar[*n*]arenes are reported here. These macrocycles, among others, were tested as catalysts for alkylation reactions in aqueous solutions. Telluride-pillar[5]arene (**P[5]-TePh**) showed the best performance, emulating the behavior of the methyltransferase enzyme cofactor *S*-adenosyl-l-methionine. Using 1.0 mol % of **P[5]-TePh**, benzyl bromides reacted with NaCN/NaN_3_ in water, yielding organic nitriles/azides. The catalyst was recycled and efficiently reused for up to six cycles. ^1^H NMR experiments indicate a possible interaction between the substrate and **P[5]-TePh**’s cavity.

## Introduction

**Figure 1:**
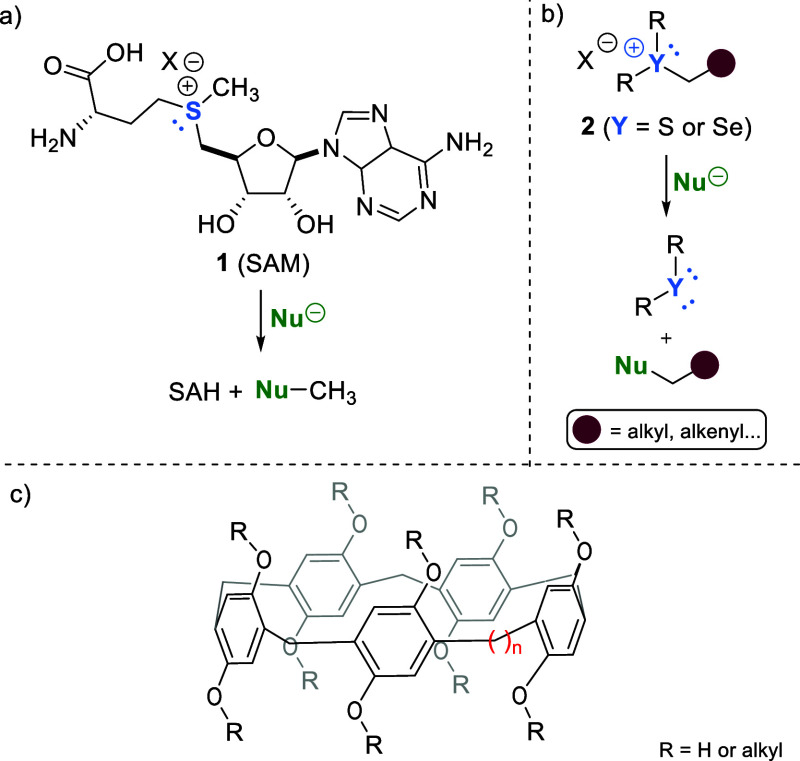
(a) *S*-adenosyl-L-methionine (SAM) **1**, an electrophile for methylation of nucleophiles; (b) chalcogenonium salts **2** as alkylating agents; (c) representation of a pillar[*n*]arene.

Enzymes are great catalysts with impressive efficiency, specificity, and selectivity.^1,2^ Methyltransferases, for instance, promote the transference of a methyl group to a variety of substrates (e.g., proteins, lipids, RNA, and DNA) in biological processes related to metabolism, biosynthesis, and detoxification of exogenous compounds.^3−5^ The cofactor of this enzyme is a sulfonium salt named *S*-adenosyl-l-methionine (SAM) **1**, which is the donor of the methyl group to a given nucleophile (Figure 1a).^6−9^ Inspired by the transformation mediated by methyltransferases, many reports in the literature have been disclosed with a focus on the preparation and utilization of SAM and derivatives (including its selenium analog) and their application as group transfer agents in a broader sense.^10−15^ Structurally simpler SAM derivatives **2**, most as sulfonium or selenonium salts,^16−22^ but rarely telluronium salts,^23,24^ have also been applied as alkylating agents (Figure 1b).

Synthetic macrocycles with tailored cavities can produce noncovalent bonding interactions with different substrates, resulting in the stabilization and organization of intermediates, offering a different environment for chemical reactions.^25^ Pillar[*n*]arenes have emerged as a new generation of supramolecular macrocyclic hosts in the past decade (Figure 1c).^26,27^ These macrocycles can form inclusion complexes with different small molecules through dipole–dipole interactions, hydrogen bonding, π–π stacking, etc.^15,28−46^ They have shown several promising applications for drug delivery,^31−34^ as nanomaterials,^35−37^ sensors,^38−40^ and as transmembrane channels.^41−43^ In organic synthesis, the use of pillar[*n*]arenes is still little explored, especially owing to their application as catalysts.^44−46^ Xiao and co-workers reported the synthesis of pillar[5]arenes which when combined with PdCl_2_(CH_3_CN)_2_, could efficiently catalyze Heck coupling reactions of styrene and aryl halides.^44^ In 2019, Lan and co-workers described a cross-linked porous polymeric material based on pillar[5]quinone, which was used to load Pd-catalyst and prepare a heterogeneous catalyst. The catalyst was highly efficient for Suzuki-coupling reactions and could be recycled and reused six times without any drop in reaction yield.^45^ Recently, a pillar[5]arene-based [2]rotaxane was designed and developed by Guo and co-workers, which, upon coordination with Pd ions, was efficiently used to catalyze Suzuki couplings.^46^ Although less reported, there are examples of pillararenes catalyzing reactions in aqueous environments, demonstrating their significant potential as catalysts.^47,48^

Accordingly, as part of our interest in developing new organochalcogen compounds with privileged molecular structures for different applications,^49−53^ allied to the synthesis and application of pillar[*n*]arenes,^54−57^ we report herein the synthesis of chalcogen-based pillar[5]arenes, including unknown sulfide- and telluride-based pillar[*n*]arenes. Additionally, we screened their catalytic activity in promoting the alkylation of nucleophiles dissolved in aqueous solutions and the possibility of recycling and reusing them.

## Results and Discussion

The monomeric catalysts (**M-YPh**, where Y = S, Se, or Te) depicted in Table 1 employed in this study were prepared from the corresponding monomeric bromides (**M-Br**).^54^ Likewise, the pillar[*n*]arene catalysts (**P[***n***]-YPh**, where *n* is 5 or 6 and Y = S, Se, or Te) were obtained from the bromide starting material. To date, only the selenium-pillar[*n*]arene analog is known.^58^ All catalysts were obtained in good yields (see the Supporting Information for the experimental details). To test their ability to promote an alkylation reaction in aqueous solution, we chose the conversion of benzyl bromide **3a** to the corresponding cyanide **4a** as the model experiment (Table 1).^59−61^ Without a catalyst, the reaction of **3a** and 2 equiv of NaCN produced only 15% of product **4a** after stirring the reaction at room temperature for 24 h as previously reported (entry 1).^16^ Addition of 5.0 mol % of monomeric catalysts accelerated product formation, especially for the tellurium analog **M-TePh**. In that case, 45% of benzyl nitrile **4a** was obtained after 24 h (entry 4). Next, chalcogen-based pillar[*n*]arenes were screened. At this time, however, 1.0 mol % of the catalyst was used to keep the same amount of chalcogen in the reaction media (entries 5–10). As observed for the monomeric species, the reaction showed a clear trend between the nature of the chalcogen and the yield observed for product **4a**. Telluride **P[5]-TePh** outperformed all other catalysts, delivering the product in near quantitative yield after 24 h of reaction (Entry 7). We performed experiments with shorter duration and observed that after 12 h the reaction was essentially done (entries 8 and 9). It is noteworthy that the contribution of the pillar[*n*]arene scaffold to the reaction outcome. Keeping the catalytic amount of chalcogen the same in all experiments, **4a** formation was significantly increased using **P[5]-TePh** compared to the reaction using **M-TePh** (entries 4 and 7). Moreover, it was observed that the pillar[*n*]arene cavity size was not critical for the catalytic activity under identical reaction conditions. **P[6]-TePh** performed similarly to **P[5]-TePh** (entries 7 and 10). Therefore, we continued our study with **P[5]-TePh** due to its easier preparation when compared to **P[6]-TePh**.

**Table 1:**
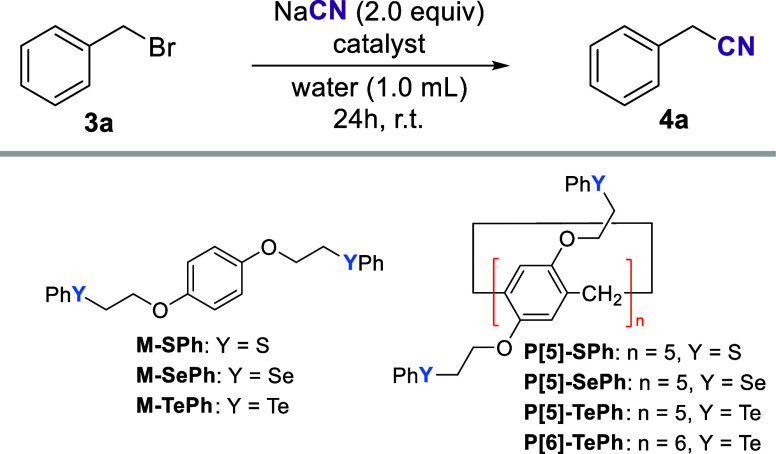
Optimization of the Reaction Conditions^a^

entry	catalyst	mol %	yield (%)^b^
1	none	0.0	15
2	**M-SPh**	5.0	5
3	**M-SePh**	5.0	29
4	**M-TePh**	5.0	45
5	**P[5]-SPh**	1.0	22
6	**P[5]-SePh**	1.0	55
7	**P[5]-TePh**	1.0	96
8^c^	**P[5]-TePh**	1.0	92
9^d^	**P[5]-TePh**	1.0	40
10	**P[6]-TePh**	1.0	85

a Reaction conditions: benzyl bromide (0.174 mmol), catalyst (0.0–5.0 mol %), NaCN (0.348 mmol) in H_2_O (1.0 mL) at 25 °C for 24 h.

b Isolated yield.

c 12 h of reaction.

d 6 h of reaction.

**Figure 2:**
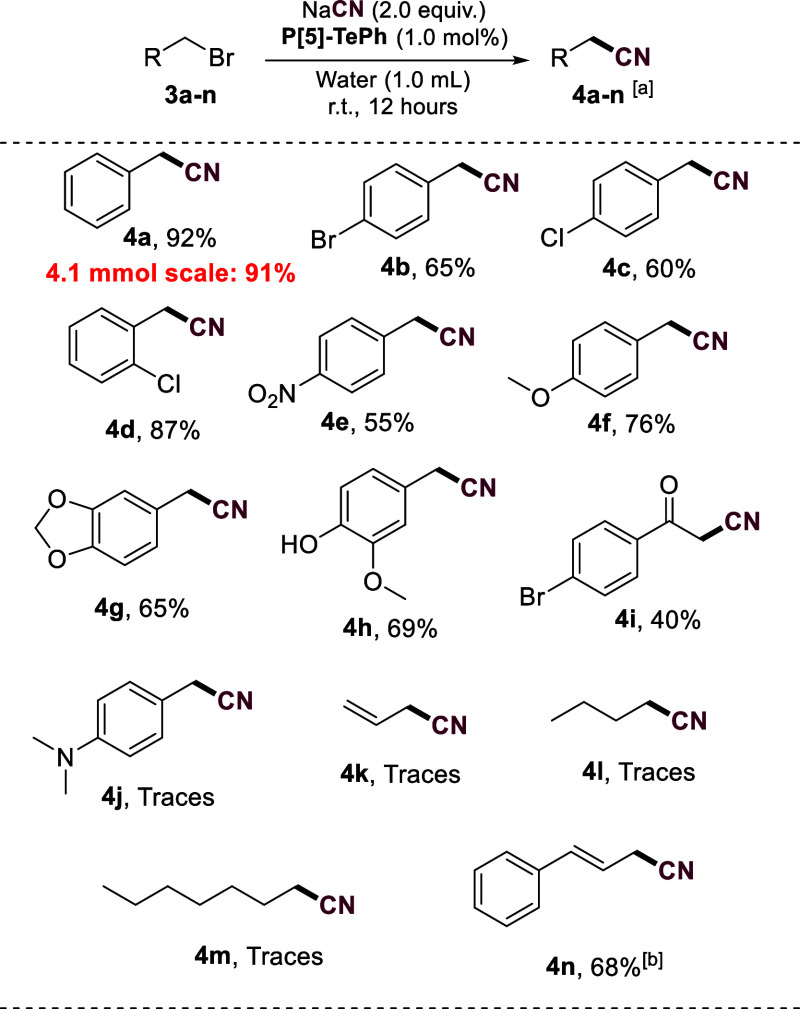
Substrate scope for conversion of halides to cyanides catalyzed by **P[5]-TePh**. ^a^Isolated yield. ^b^Cinnamyl chloride was used as substrate.

Next, we applied the best reaction conditions to convert other substrates to nitriles catalyzed by 1.0 mol % of **P[5]-TePh** in aqueous solution (Figure 2). First, we observed that the yield of the conversion of benzyl bromide to **3a** was not affected on a larger scale. Then, aryl bromides assembled with electron-donating or electron-withdrawing groups were converted to the corresponding nitriles **4b**–**h** in reasonable to good yields. Noteworthy, no clear correlation between the electronic properties and product yield was observed for these substrates. Product **4i** obtained in 40% yield revealed that α-carbonyl bromides are feasible substrates for this transformation. Additionally, nitrile **4n** was prepared in 68% yield when the same reaction conditions were applied using cinnamyl chloride as the substrate. On the contrary, products **4j**-**m** from an electron-rich benzyl bromide, allyl bromide, or alkyl bromides could not be produced in desirable amounts. To improve the yield of product **4j**, we prepared the bromide starting material and immediately used it in the reaction. However, the strong electron donation capacity of the dimethylamino substituent triggered its decomposition faster than the reaction with the nucleophile. The results collectively indicate that at the current stage, regardless of the substrate solubility in water, only activated starting materials toward displacement reactions are feasible substrates for the transformation. The reaction conditions for effective conversions of less reactive aliphatic halides or to obtain products with lower boiling points should be better designed. Finding better catalysts is crucial in this context.

**Figure 3:**
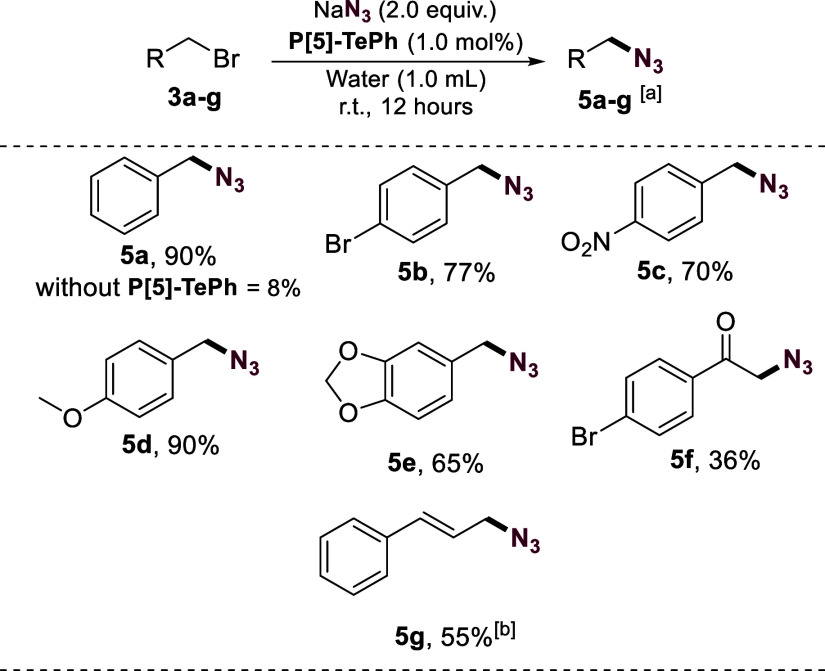
Substrate scope for conversion of halides to azides catalyzed by **P[5]-TePh**. ^a^Isolated yield. ^b^Cinnamyl chloride was used as substrate.

To demonstrate the effectiveness of this protocol, we studied the conversion of selected substrates to azides using NaN_3_ as the nucleophile (Figure 3). Gratifyingly, benzyl bromide was converted to respective azide **5a** in 90% yield. Importantly, without **P[5]-TePh,** only 8% **5a** was obtained. Other benzyl bromides containing electron-donating or electron-withdrawing groups, an α-carbonyl derivative, and cinnamyl chloride were used as substrates. Products **5b**-**5g** were obtained in reasonable to good yields, mirroring the results obtained with cyanide as the nucleophilic species.

**Figure 4:**
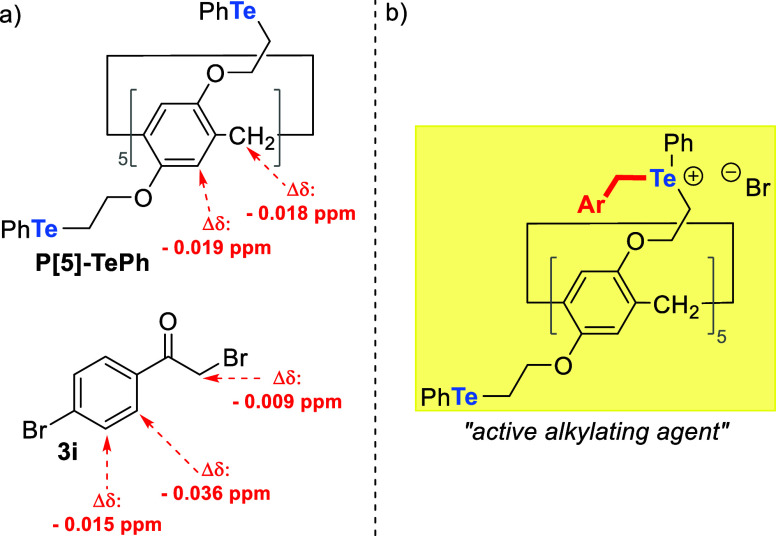
(a) Signal shifts observed by ^1^H NMR of a mixture **P[5]-TePh** and **3i** in CDCl_3_; (b) structure of the proposed active alkylating agent.

Due to their electron-rich cavities, pillar[*n*]arenes have excellent host–guest properties, forming stable complexes through charge transfer interactions (e.g., cation-π interactions, CH-π interactions, and π–π stacking).^31−34^ To further understand the interactions between substrates and **P[5]-TePh** in the reaction, we performed ^1^H NMR analysis of a mixture of **P[5]-TePh** and bromide **3i**, chosen because it has a higher molecular weight (more experimental details and ^1^H NMR spectra are given in the Supporting Information). Although small, various signal shifts could be observed for catalyst and bromide **3i** (Figure 4a). These results might suggest the formation of an inclusion complex between the pillar[*n*]arene and the substrate. In addition, since signal shifts were observed for the aromatic protons of **3i**, we suggest that the aryl group of **3i** could be engulfed into the **P[5]-TePh** cavity, while the CH_2_ group was not. These observations are consistent with previous reports, considering that the cavity size of the pillar[5]arenes can accommodate a benzene ring^62^ and lead to the formation of an efficient host–guest complex.^63−69^

Nevertheless, the catalytic activity of pillar[*n*]arene catalysts **P[*n*]-YPh** as an alkylating agent is not solely related to the size of the cavity or its host–guest interactions with the substrate. The chalcogen atom is crucial in the substrate conversion to products. Based on our previous results and reported literature,^70−73^ we propose that the active species is a telluronium salt, as depicted in Figure 4b.

**Figure 5:**
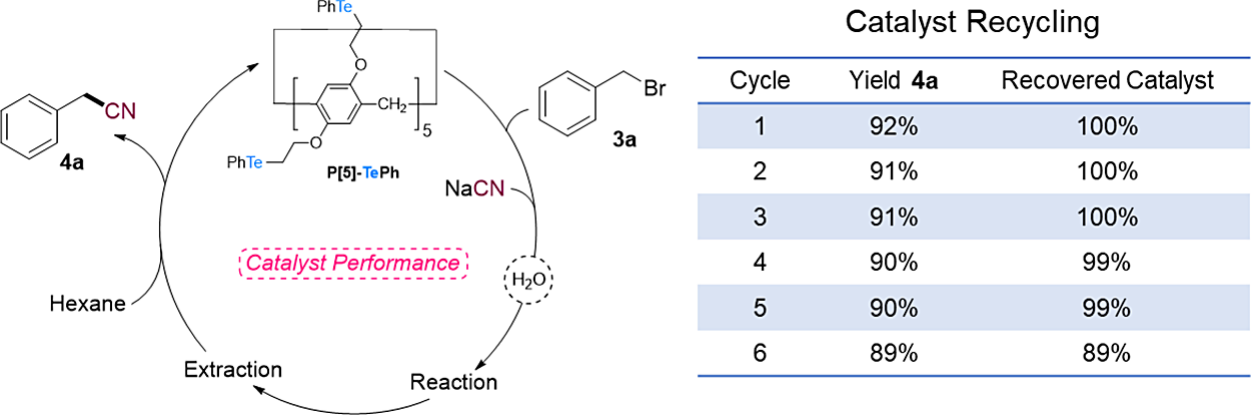
Evaluation of recovery and reuse of **P[5]-TePh** on conversion of BnBr to **4a**.

Finally, developing safer and greener organic reactions is a major goal nowadays.^74,75^ Accordingly, in addition to water being used as the reaction solvent, we studied the possibility of recovering and reusing catalyst **P[5]-TePh**. To our delight, we found that after the extraction of the reaction mixture, benzyl bromide and product **4a** could be separated from **P[5]-TePh** by washing the crude mixture with hexanes. In this way, the catalyst could be reused for another reaction after water and NaCN addition. This process could be efficiently repeated during 5 cycles with excellent catalyst recovery and without reducing the reaction yield on **4a** formation. Although the reaction yield remained constant, after the fifth cycle, a drop in the recovery of the catalyst was observed (Figure 5).

## Conclusions

In conclusion, we have described, for the first time, the synthesis of sulfur- and tellurium-based pillar[*n*]arenes. These novel macrocycles were obtained in excellent yields via a simple nucleophilic substitution reaction from the corresponding bromide. The ability of monomeric catalysts **M-YPh** (5.0 mol %) and pillar[*n*]arene catalysts **P[*****n*****]-YPh** (1.0 mol %) to promote the conversion of bromides to the related nitriles or azide in an aqueous solution containing NaCN or NaN_3_ was investigated. It was found that the chalcogen nature and the pillar cavity size are critical for catalytic performance. ^1^H NMR experiments of mixtures of **P[5]-TePh** and a substrate indicated possible host–guest interactions in the form of an inclusion complex. Moreover, it was feasible to perform the reaction on a larger scale, and the catalyst **P[5]-TePh** could be recovered and reused effectively for five reaction cycles. Inspired by these results, we are pursuing further developments, including the design of new chalcogen-pillar[*n*]arenes and their application to a broader panel of organic transformations.

## Experimental Section

### General Remarks

The reactions were monitored by TLC carried out on Merck silica gel (60 F254) using UV light as a visualizing agent, an iodine chamber, and 5% vanillin in 10% H_2_SO_4_ and heat as developing agents. Baker silica gel (particle size 0.040–0.063 mm) was used for flash chromatography. Proton nuclear magnetic resonance spectra (^1^H NMR) were obtained on a Varian AS-400 or a Bruker Avance NEO 500 MHz employing a direct broadband probe at 500 MHz. Spectra are recorded in CDCl_3_ solutions. Chemical shifts are reported in parts per million, referenced to tetramethylsilane (TMS) as the internal reference. Coupling constants (*J*) are reported in Hertz. Abbreviations to denote the multiplicity of a particular signal are s (singlet), d (doublet), dd (doublet of doublets), ddd (doublet of doublet of doublets), q (quartet), quint (quintet), sex (sextet), t (triplet), and m (multiplet). Carbon-13 (^13^C{1H} NMR) nuclear magnetic resonance spectra and Carbon-13-attached proton test (^13^C{1H}-APT NMR) nuclear magnetic resonance spectra were obtained on a Varian AS-400 or on Bruker Avance NEO 500 MHz employing a direct broadband probe at 125 MHz. The high-resolution atmospheric pressure chemical ionization mass spectrometry (APCI-QTOF) and electrospray ionization (ESI-QTOF) mass spectrometry analyses were performed on a Bruker Daltonics micrOTOF-Q II instrument in operating positive mode. The samples were solubilized in HPLC-grade acetonitrile and injected into the source by means of a syringe pump at a flow rate of 5.0 μL min-1. The following instrument parameters were applied: capillary and cone voltages were set to +4000 and −500 V, respectively, with a desolvation temperature of 180 °C. For data acquisition, processing, and isotope simulations, Compass 1.3 for micrOTOF-Q II software (Bruker daltonics, USA) was used. Melting point (mp) values were measured in a Fisatom 430D instrument with a 0.1 °C precision. The Fourier transform infrared (FTIR) measurements were performed on a Nicolet iS50 (Thermo Fisher Scientific). UV–visible absorption spectra were obtained in the UV/visible range (from 200 to 800 nm) using a Varian Cary 50 Scan spectrophotometer and quartz cuvettes with a path length of 10 mm and 1.5 mL. UV–visible spectra were recorded using dichloromethane. Final concentrations of compounds: **P[5]Br**, **P[5]TePh**, and **P[6]TePh** = 1,0 μM. **P[6]Br** = 0,2 μM.

### General Procedure for Alkylation of NaCN Catalyzed by **P[5]-TePh**

A 10.0 mL round-bottomed glass vial was added with the appropriate alkyl bromide **3a**-**n** (0.174 mmol), **P[5]-TePh** (0.00174 mmol, 8.0 mg; 1.0 mol %), and water (1.0 mL). The resulting mixture was stirred at room temperature for 5 min. After this, NaCN (0.348 mmol, 17.1 mg) was added, and the mixture was stirred for an additional 12 h. The reactions were monitored by TLC until the total disappearance of the starting materials (the progress of the reaction could also be visually observed. See Figure S1). After that, the reaction mixture was extracted with ethyl acetate (3 × 15.0 mL). The combined organic layers were dried over Na_2_SO_4_ and concentrated under a vacuum. The residue was purified by preparative TLC using hexane/ethyl acetate (90:10) as the eluent.

## Data Availability

The data underlying this study are available in the published article and its Supporting Information.

## Supporting Information Available

General procedures for compounds **M-SPh**, **M-SePh**, **M-TePh**, **P[5]-SPh**, **P[5]-SePh**, **P[5]-TePh**, **P[6]-TePh;** general procedure for the synthesis of compounds **4a**–**n** and **5a**–**g;** general procedure for Gram-scale reaction; general procedure for recovery and reuse of the catalyst **P[5]-TePh;** experiments to detect the interactions between substrates and **P[5]-TePh;** and spectral data and copies of NMR spectra for all compounds (PDF)
