# Supplementary material for: Telluride-Based Pillar[5]arene: A Recyclable Catalyst for Alkylation Reactions in Aqueous Solution
Source: J Org Chem. 2024 Sep 5;89(18):12982–8. doi: 10.1021/acs.joc.4c00997 (PMC11421007; doi:10.1021/acs.joc.4c00997)
Supplement: Supplementary file 1 — jo4c00997_si_001.pdf [file jo4c00997_si_001.pdf]

SUPPORTING INFORMATION

Telluride-Based Pillar[5]arene: A Recyclable Catalyst For Alkylation Reactions In Aqueous Solution

Patrick C. Nobre,[a] Pâmella Cordeiro,[a] Ingrid C. Chipoline,[a] Victor Menezes,[a] Kaila V. S. Santos,[a] Alix Y. Bastidas Ángel,[b] Eduardo E. Alberto[b]* and Vanessa Nascimento[a]*

[a] SupraSelen Laboratory, Departament of Chemistry, Universidade Federal Fluminense. Institute of Chemistry, Campus do Valonguinho, 24020-141, Niterói-RJ, Brazil.
[b] Departamento de Química, Universidade Federal de Minas Gerais – UFMG. 31.270-901, Belo Horizonte, MG, Brazil.

*E-mail corresponding authors: nascimentovanessa@id.uff.br; albertoee@ufmg.br

Contents

General procedure for the synthesis of the **M-Chalcogen**	**S2**
General procedure for the synthesis of the **P[5]-Chalcogen**	**S4**
General procedure for the synthesis of the **P[6]-TePh**	**S6**
UV-Vis spectra of **P[n]-Br** and **P[n]-TePh**	**S6**
General procedure for alkylation of NaCN catalyzed by **P[5]-TePh**	**S7**
General procedure for alkylation of NaN_3 catalyzed by **P[5]-TePh**	**S9**
General procedure for Gram-scale reaction	**S10**
General procedure for reuse of the catalyst	**S10**
Experiments to detect the interactions between substrates and **P[5]-TePh**	**S10**
References	**S14**
Selected spectra	**S15**

General procedure for synthesis of the 1,4-Bis(2-(chalcogen)ethoxy)benzene derivatives (M-Chalcogen):

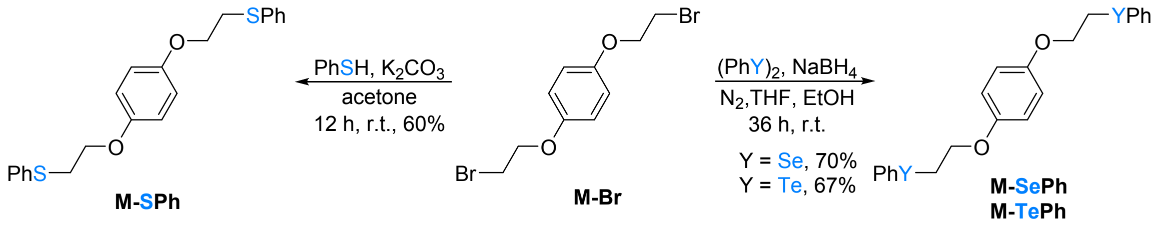

Procedure for the sulfur derivative: A 10.00 mL round-bottomed glass vial were added thiophenol (0.623 mmol, 70 mg), acetone (2.0 mL), K_2CO_3 (0.684 mmol, 94 mg) and **M-Br**[1] (0.261 mmol, 85 mg). The resulting mixture was stirred at room temperature for 15 h. The reaction was monitored by TLC until the total disappearance of the starting materials. After that, the reaction mixture was poured into a solution of 5% of the NaOH (50.0 mL), and extracted with DCM (3x 15.0 mL). The combined organic phases were dried over $MgSO_4$, and concentrated under vacuum. The residue was purified by column chromatography on silica gel 60A (0.060-0.200 mm-Across) using a 6:4 (v/v) solution of $CHCl_3$/hexane to afford **M-SPh** in 60% yield.

Spectral data of the compound

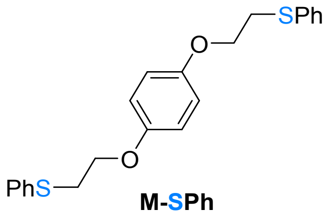

M-SPh

1,4-Bis(2-(phenylthio)ethoxy)benzene **M-SPh**: Purified by chromatography column (hexane/$CHCl_3$ = 60:40); Yield: 0.0598 g (60%); white solid, m.p.: 125-127 ºC. ^{1}H NMR ($CDCl_3$, 500 MHz) δ (ppm) = δ 7.41-7.39 (m, 4H); 7.31-7.27 (m, 4H); 7.22-7.19 (m, 2H); 6.76 (s, 4H); 4.08 (t, J = 7.0 Hz, 4H); 3.25 (t, J = 7.0 Hz, 4H). ^{13}C{^{1}H} NMR ($CDCl_3$, 125 MHz) δ = 152.8, 135.5, 129.8, 129.0, 126.5, 115.7, 67.4, 32.9. HRMS (ESI-QTOF) calculated mass for $C_{22}H_{22}O_2S_2$ $[M]^+$: 382.1061, found: 382.1007.

Procedure for the selenium and tellurium derivatives: A 25.0 mL round-bottomed double-necked flask was charged with a solution of diphenyl diselenide or diphenyl ditelluride (0.210 mmol; 65.9 mg and 86.9 mg respectively) and a mixture of dry THF (1.0 mL) and EtOH (0.5 mL). Subsequently, the system was saturated with nitrogen and $NaBH_4$ (0.525 mmol; 20.0 mg) was added to the solution. Afterward, the reaction mixture was vigorously stirred at room temperature, and after about 30 minutes, the solution became colorless, indicating the total cleavage of the chalcogen–chalcogen bond. Then, a solution of **M-Br**[1] (0.210 mmol; 67.6 mg). in dry THF (1.0 mL) was added dropwise. After the

end of the addition, the mixture was stirred at room temperature for an additional 36 hours. Then, the mixture was poured into a solution of HCl 0.1 mol L^{-1} (10.0 mL) and extracted with DCM (3 × 10.0 mL). The combined organic layers were dried over Mg_2SO_4, and the solvent evaporated under reduced pressure. The crude product was purified by column chromatography using a 6:4 (v/v) solution of DCM/hexane to afford **M-SePh** in 70% yield and **M-TePh** in 67% yield.

Spectral data of the compounds

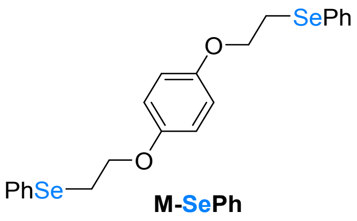

1,4-Bis(2-(phenylselanyl)ethoxy)benzene **M-SePh**:[3] Purified by chromatography column (hexane/DCM = 60:40); Yield: 0.0703 g (70%); white solid, m.p.: 98-99 ºC, lit.[3] = 90.5-93.3 ºC. ^{1}H NMR ($CDCl_3$, 500 MHz) δ (ppm) = 7.54-7.52 (m, 4H); 7.28-7.23 (m, 6H); 6.73 (s, 4H); 4.12 (t, J = 7.3 Hz, 4H); 3.18 (t, J = 7.3 Hz, 4H). ^{13}C{^{1}H} NMR ($CDCl_3$, 125 MHz) δ = 152.7, 132.9, 129.3, 129.1, 127.2, 115.6, 68.1, 26.1.

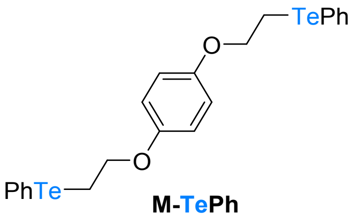

1,4-Bis(2-(phenyltellanyl)ethoxy)benzene **M-TePh**: Purified by chromatography column (hexane/DCM = 60:40); Yield: 0.0813 g (67%); white solid, m.p.: 93-95 ºC. ^{1}H NMR ($CDCl_3$, 500 MHz) δ (ppm) = δ 7.77-7.75 (m, 4H); 7.30-7.27 (m, 2H); 7.21-7.18 (m, 4H); 6.72 (s, 4H); 4.23 (t, J = 7.5 Hz, 4H); 3.16 (t, J = 7.5 Hz, 4H). ^{13}C{^{1}H} NMR ($CDCl_3$, 125 MHz) δ = 152.6, 138.6, 129.2, 127.9, 115.7, 111.0, 70.1, 7.0. ^{125}Te{^{1}H} NMR (158 MHz, CDCl3) δ = 443.8. HRMS (APCI-QTOF) calculated mass for $C_{22}H_{22}O_2Te_2$ $[M]^+$: 577,9744, found: 577,9757.

General procedure for synthesis of the Chalcogen-Pillar[5]arenes derivatives (P[5]-Chalcogen):

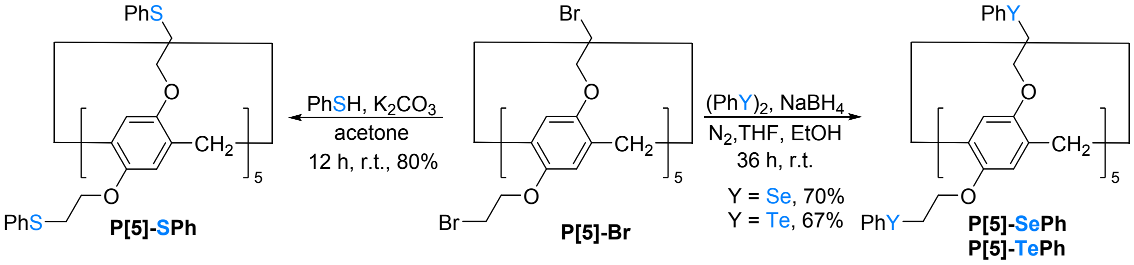

Procedure for the sulfur derivative: A 10.0 mL round-bottomed glass vial, were added the thiophenol (0.623 mmol; 68.5 mg), acetone (1.5 mL), K_2CO_3 (0.684 mmol, 94.4 mg) and **P[5]-Br**[1] (0.051 mmol; 85.2 mg). The resulting mixture was stirred at room temperature for 15 hours. The reaction we monitored by TLC until the total disappearance of the starting materials. After that, the reaction mixture was poured into a solution of 5% of the NaOH (50.0 mL) and extracted with ethyl acetate (3 x 15.0 mL). The combined organic layers were dried over $MgSO_4$ and concentrated under vacuum. The residue was purified by column chromatography on silica gel 60A (0.060-0.200 mm-Across) using a 6:4 (v/v) solution of $CHCl_3$/hexane to afford **P[5]-SPh** in 80% yield.

Spectral data of the compound

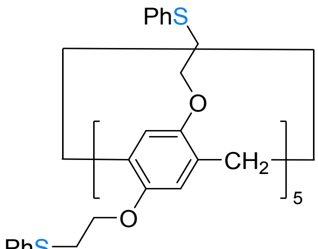

P[5]-SPh

Compound **P[5]-SPh**: Purified by chromatography column (hexane/$CHCl_3$ = 60:40); Yield: 0.0803 g (80%); yellowish oil. ^{1}H NMR ($CDCl_3$, 500 MHz) δ (ppm) = δ 7.34-7.32 (m, 20H); 7.24-7.21 (m, 20H); 7.17-7.13 (m, 10H); 6.87 (s, 10H); 4.16-4.12 (m, 10H); 3.97-3.92 (m, 10H); 3.72 (s, 10H); 3.20-3.16 (m, 20H). ^{13}C{^{1}H} NMR ($CDCl_3$, 125 MHz) δ = 149.7, 136.2, 129.3, 129.0, 128.7, 126.2, 115.6, 67.3, 33.7, 29.3. HRMS (ESI-QTOF) calculated mass for $C_{115}H_{110}O_{10}S_{10}$ $[M+H]^+$: 1971,5379, found: 1971.5302.

Procedure for the Selenium and Tellurium derivatives: A 25 mL round-bottomed double-necked flask was charged with a solution of diphenyl diselenide or diphenyl ditelluride (0.214 mmol; 67.2 mg and 88.6 mg respectively) in a mixture of dry THF (1.0 mL) and EtOH (0.5 mL). Subsequently, the system was saturated with nitrogen and $NaBH_4$ (0.536 mmol; 20.4 mg) was added to the solution. After the addition, the reaction mixture was vigorously stirred at room temperature, and after about 30 minutes, the solution became colorless, indicating the total cleavage of the chalcogen–chalcogen bond. Then, a solution of **P[5]-Br**[1] (0.043 mmol; 71,8 mg) in dry THF (1.0 mL) was added dropwise. After the end of the addition, the mixture was stirred at room temperature for an additional 36 hours. Then, the mixture was poured into a solution of HCl 0.1 mol L^{-1} (10.0 mL) and extracted with DCM (3 × 10.0 mL). The combined organic layers were dried over Mg_2SO_4, and the solvent evaporated under reduced pressure. The crude product was purified by preparative TLC using a solution of DCM/hexane [1:1; (v/v)] to afford **P[5]-SePh** in 91% yield and **P[5]-TePh** in 70% yield.

Spectral data of the compounds

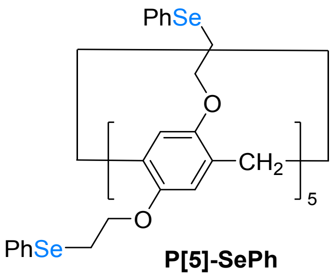

Compound **P[5]-SePh**:[3] Purified by preparative TLC (hexane/DCM = 50:50); Yield: 0.0737 g (70%); yellowish oil. ^{1}H NMR ($CDCl_3$, 500 MHz) δ (ppm) = δ 7.50-7.48 (m, 20H); 7.21-7.20 (m, 30H); 6.87 (s, 10H); 4.23-4.19 (m, 10H); 4.05-4.00 (m, 10H); 3.71 (s, 10H); 3.21-3.14 (m, 20H). ^{13}C{^{1}H} NMR ($CDCl_3$, 125 MHz) δ = 149.6, 132.5, 130.1, 129.2, 128.6, 127.0, 115.5, 68.1, 29.3, 27.1.

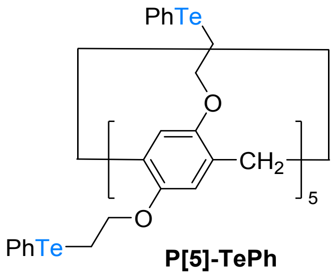

Compound **P[5]-TePh**: Purified by preparative TLC (hexane/DCM = 50:50); Yield: 0.0850 g (67%); yellowish oil. ^{1}H NMR ($CDCl_3$, 500 MHz) δ (ppm) = δ 7.73-7.72 (m, 20H); 7.25-7.21 (m, 10H); 7.17-7.14 (m, 20H); 6.85 (s, 10H); 4.37-4.11 (m, 10H); 4.15-4.11 (m, 10H); 3.72 (s, 10H); 3.30-3.25 (m, 10H), 3.16-3.11 (m, 10H). ^{13}C{^{1}H} NMR ($CDCl_3$, 125 MHz) δ = 150.1, 138.2, 129.2, 127.9, 127.6, 115.6, 111.8, 70.0, 29.7, 8.0. ^{125}Te{^{1}H} NMR ($CDCl_3$, 158 MHz) δ (ppm) = 440.3. IR (neat) = υ (cm^{-1}) 3049; 2923; 2851; 1495; 1402; 1016; 726.

General procedure for synthesis of Tellurium-Pillar[6]arene derivative (P[6]-TePh):

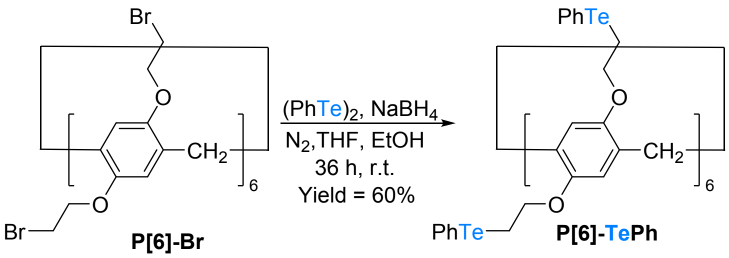

A 25.0 mL round-bottomed double-necked flask was charged with a solution of diphenyl ditelluride (0.214 mmol; 88.6 mg) in a mixture of dry THF (1.0 mL) and EtOH (0.5 mL). Subsequently, the system was saturated with nitrogen and $NaBH_4$ (0.536 mmol; 20.4 mg) was added to the solution. After the addition, the reaction mixture was vigorously stirred at room temperature, and after about 30 min, the solution became colorless, indicating the total cleavage of the chalcogen–chalcogen bond. Then, a solution of **P[6]-Br**[2] (0.043 mmol, 85.6 mg) in dry THF (1.0 mL) was added dropwise. After the end of the addition, the mixture was stirred at room temperature for an additional 36 hours. Then, the mixture

was poured into a solution of HCl 0.1 mol L^{-1} (10.0 mL) and extracted with DCM (3 × 10.0 mL). The combined organic layers were dried over Mg_2SO_4, and the solvent evaporated under reduced pressure. The crude product was purified by preparative TLC using a 1:1 (v/v) solution of DCM/hexane to afford **P[6]-TePh** in 60% yield.

Spectral data of the compound

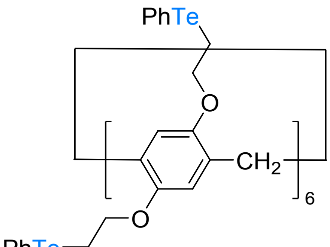

P[6]-TePh

Compound **P[6]-TePh**: Purified by preparative TLC (hexane/DCM = 50:50); Yield: 0.0850 g (60%); yellowish oil. ^{1}H NMR ($CDCl_3$, 500 MHz) δ (ppm) = δ 7.62 (d, J = 7.3 Hz, 24H); 7.18 (t, J = 7.3 Hz, 12H); 7.09 (t, J = 7.3 Hz, 24H); 6.69 (s, 12H); 4.13-4.10 (m, 24H); 3.73 (s, 12H); 3.07-3.04 (m, 24H). ^{13}C{^{1}H} NMR ($CDCl_3$, 125 MHz) δ = 150.1, 138.2, 129.2, 127.9, 127.6, 115.6, 111.8, 70.0, 29.7, 8.0. ^{125}Te{^{1}H} NMR ($CDCl_3$, 158 MHz) δ (ppm) = 443.4. IR (neat) = υ (cm^{-1}) 3050; 2921; 2852; 1498; 1405; 1202; 1016; 726; 689.

UV-Vis spectra of P[n]-Br and P[n]-TePh

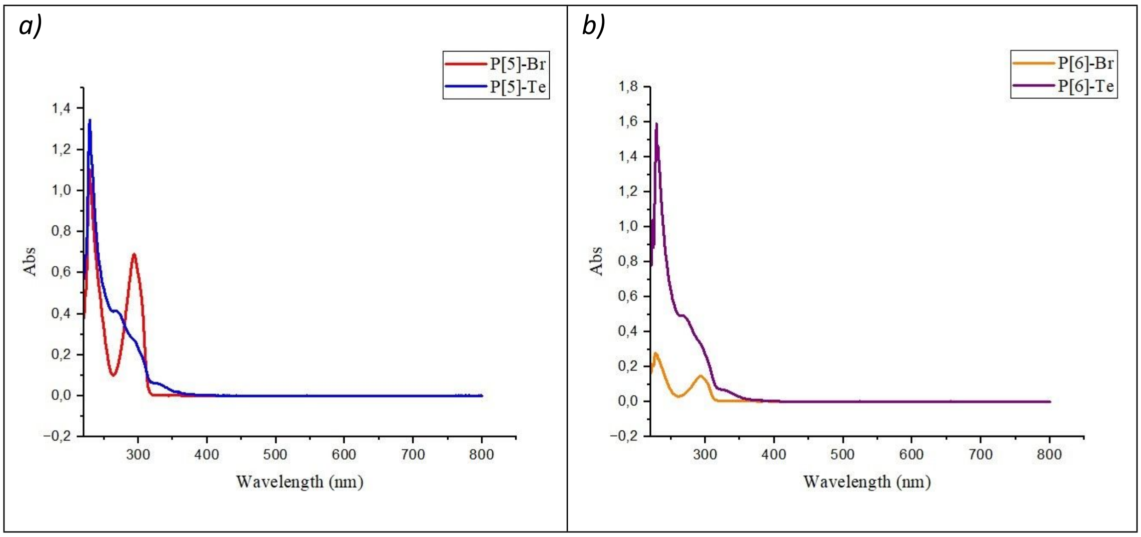

a) **P[5]Br** and **P[5]TePh**, 1,0 μM in CH_2Cl_2. b) **P[6]Br** and **P[6]TePh**, 0,2 μM and 1,0 μM in CH_2Cl_2, respectively.

General procedure for alkylation of NaCN catalyzed by P[5]-TePh:

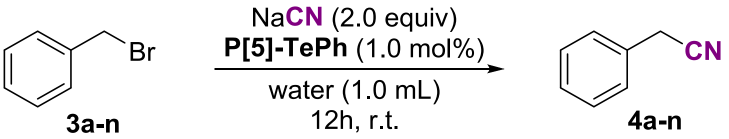

A 10.0 mL round-bottomed glass vial was added the appropriate alkyl bromide **3a-n** (0.174 mmol), **P[5]-TePh** (0.00174 mmol, 8.0 mg; 1.0 mol%) and water (1.0 mL). The resulting mixture was stirred at room temperature for 5 minutes. After this, NaCN (0.348 mmol, 17.1 mg) was added, and the mixture was stirred for an additional 12 hours. The reactions were monitored by TLC until the total disappearance of the starting materials (the progress of the reaction could also be visually observed. See Figure S1). After that, the reaction mixture was extracted with ethyl acetate (3x 15.0 mL). The combined organic layers were dried over Na_2SO_4 and concentrated under vacuum. The residue was purified by preparative TLC using hexane/ethyl acetate [90:10; (v/v)] as the eluent.

Spectral data of the compounds

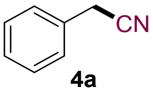

4a

2-Phenylacetonitrile **4a**:[4] Purified by preparative TLC (hexane/ethyl acetate = 90:10); Yield: 0.0187 g (92%); colorless oil. ^{1}H NMR ($CDCl_3$, 500 MHz) δ (ppm) = δ 7.33-7.30 (m, 2H); 7.27-7.25 (m, 3H); 3.68 (s, 2H). $^{13}C\{^{1}H\}$ NMR ($CDCl_3$, 125 MHz) δ = 129.9, 129.1, 128.8, 127.9, 117.8, 23.6.

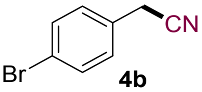

4b

2-(4-Bromophenyl)acetonitrile **4b**:[4] Purified by preparative TLC (hexane/ethyl acetate = 90:10); Yield: 0.0220 g (65%); white solid; mp.: 51-52 °C, lit.[4]: 46.3-47.6. ^{1}H NMR ($CDCl_3$, 500 MHz) δ (ppm) = δ 7.44 (d, J = 8.5 Hz, 2H); 7.14 (d, J = 8.5 Hz, 2H); 3.63 (s, 2H). $^{13}C\{^{1}H\}$ NMR ($CDCl_3$, 125 MHz) δ = 132.2, 129.5, 128.8, 122.1, 117.3, 23.1.

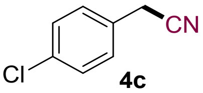

4c

2-(4-Chlorophenyl)acetonitrile **4c**:[5] Purified by preparative TLC (hexane/ethyl acetate = 90:10); Yield: 0.0158 g (60%); yellowish oil ^{1}H NMR ($CDCl_3$, 500 MHz) δ (ppm) = δ 7.29 (d, J = 8.5 Hz, 2H); 7.20 (d, J = 8.5 Hz, 2H); 3.66 (s, 2H). $^{13}C\{^{1}H\}$ NMR ($CDCl_3$, 125 MHz) δ = 134.2, 129.3, 129.3, 128.3, 117.4, 23.1.

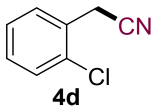

4d

2-(2-Chlorophenyl)acetonitrile **4d**:[5] Purified by preparative TLC (hexane/ethyl acetate = 90:10); Yield: 0.0229 g (87%); white oil; ^{1}H NMR ($CDCl_3$, 500 MHz) δ (ppm) = δ 7.46-7,44 (m, 1H); 7.36-7.34 (m, 1H); 7.25-7.23 (m, 2H); 3.77 (s, 2H). ^{13}C{^{1}H} NMR ($CDCl_3$, 125 MHz) δ = 133.5, 129.8, 129.7, 129.6, 128.2, 127.5, 116.8, 22.1.

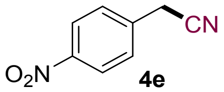

4e

2-(4-Nitrophenyl)acetonitrile **4e**:[8] Purified by preparative TLC (hexane/ethyl acetate = 90:10); Yield: 0.0155 g (55%); yellowish solid; mp.: 114-115 °C, lit.[7]: 116-117 °C . ^{1}H NMR ($CDCl_3$, 500 MHz) δ (ppm) = δ 8.14 (d, J = 8.8 Hz, 2H); 7.49 (d, J = 8.8 Hz, 2H); 4.45 (s, 2H). ^{13}C{^{1}H} NMR ($CDCl_3$, 125 MHz) δ = 147.8, 137.0, 132.8, 128.9, 124.3, 116.4, 23.5.

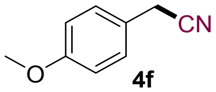

4f

2-(4-Methoxyphenyl)acetonitrile **4f**:[4] Purified by preparative TLC (hexane/ethyl acetate = 90:10); Yield: 0.0194 g (76%); colorless oil. ^{1}H NMR ($CDCl_3$, 500 MHz) δ (ppm) = δ 7.23 (d, J = 8.8 Hz, 2H); 6.90 (d, J = 8.8 Hz, 2H); 3.81 (s, 3H); 3.68 (s, 2H). ^{13}C{^{1}H} NMR ($CDCl_3$, 125 MHz) δ = 159.3, 129.1, 121.7, 118.2, 114.5, 55.3, 22.8.

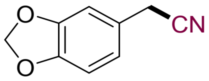

4g

2-(Benzo[d][1,3]dioxol-5-yl)acetonitrile **4g**:[4] Purified by preparative TLC (hexane/ethyl acetate = 90:10); Yield: 0.0182 g (65%); brown solid; m.p.: 51-52 °C, Lit.: 49-50 °C. ^{1}H NMR ($CDCl_3$, 500 MHz) δ (ppm) = δ 6.79-6.78 (m, 3H); 5,98 (s, 2H); 3.65 (s, 2H). ^{13}C{^{1}H} NMR ($CDCl_3$, 125 MHz) δ = 148.3, 147.4, 123.3, 121.2, 117.9, 108.6, 108.3, 101.4, 23.2.

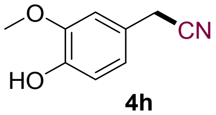

4h

2-(4-Hydroxy-3-methoxyphenyl)acetonitrile **4h**:[6] Purified by preparative TLC (hexane/ethyl acetate = 90:10); Yield: 0.0196 g (69%); yellowish oil. ^{1}H NMR ($CDCl_3$, 500 MHz) δ (ppm) = δ 6.89 (d, J = 8.0 Hz, 1H); 6.82-6.78 (m, 2H); 3.90 (s, 3H); 3.68 (s, 2H). ^{13}C{^{1}H} NMR ($CDCl_3$, 125 MHz) δ = 146.9, 145.4, 121.4, 121.0, 118.2, 114.8, 110.3, 56.0, 23.2.

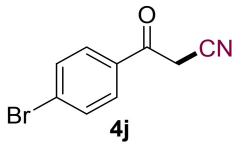

4j

3-(4-Bromophenyl)-3-oxopropanenitrile **4i**:[4] Purified by preparative TLC (hexane/ethyl acetate = 90:10); Yield: 0.0155 g (40%); yellowish solid; m.p.: 158-160 °C, lit.[4]: 156.9 -158.2 °C. ^{1}H NMR (Acetone-d$_6$, 500 MHz) δ (ppm) = δ 7.95 (d, J = 8.5 Hz, 2H); 7.77 (d, J = 8.5 Hz, 2H); 4.60 (s, 2H). ^{13}C{^{1}H} NMR ($CDCl_3$, 125 MHz) δ = 188.9, 134.8, 132.9, 131.0, 129.4, 115.3.

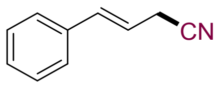

4l

(E)-4-Phenylbut-3-enenitrile **4j**:[4] Purified by preparative TLC (hexane/ethyl acetate = 90:10); Yield: 0.0169 g (68%); yellowish oil. ^{1}H NMR ($CDCl_3$, 500 MHz) δ (ppm) = δ 7.38-7.32 (m, 5H); 6.73 (dt, J = 15.8 and 1.7 Hz, 1H); 6.07-6.02 (m, 1H); 3.28 (dd, J = 5.7 and 1.7 Hz, 2H). ^{13}C{^{1}H} NMR ($CDCl_3$, 125 MHz) δ = 135.6, 134.6, 128.7, 128.3, 126.4, 117.3, 116.7, 20.7.

General procedure for alkylation of NaN$_3$ catalyzed by P[5]-TePh:

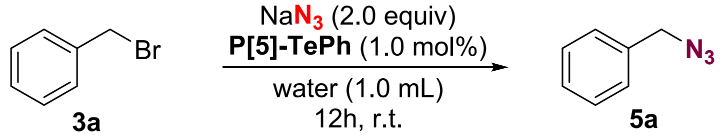

A 10.0 mL round-bottomed glass vial was added the alkyl bromide **3a-f** (0.174 mmol), **P[5]-TePh** (0.00174 mmol, 8.0 mg; 1 mol%) and water (1.0 mL). The resulting mixture was stirred at room temperature for 5 min. After this, NaN_3 (0.348 mmol, 22.6 mg) was added and the mixture was stirred for an additional 12 hours. The reaction was monitored by TLC until the total disappearance of the starting materials. After that, the reaction mixture was extracted with ethyl acetate (3x 15.0 mL). The combined organic layers were dried over Na_2SO_4 and concentrated under vacuum. The residue was purified by preparative TLC using hexane/ethyl acetate [90:10; (v/v)] as the eluent.

Spectral data of the compound

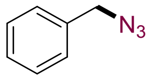

5a

(Azidomethyl)benzene **5a**:[7] Purified by preparative TLC (hexane/ethyl acetate = 90:10); Yield: 0.0208 g (90%); yellowish oil. ^{1}H NMR ($CDCl_3$, 500 MHz) δ (ppm) = δ 7.37-7.28 (m, 5H); 4.29 (s, 2H). ^{13}C{^{1}H} NMR ($CDCl_3$, 125 MHz) δ = 135.3, 128.7, 128.2, 128.1, 54.7.

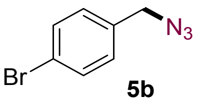

5b

1-(Azidomethyl)-4-bromobenzene **5b**:[9] Purified by preparative TLC (hexane/ethyl acetate = 90:10); Yield: 0.0283 g (77%); colorless oil. ^{1}H NMR ($CDCl_3$, 500 MHz) δ (ppm) = δ 7.51 (d, J = 8.3 Hz; 2H); 7.19 (d, J = 8.3 Hz; 2H); 4.30 (s, 2H). ^{13}C{^{1}H} NMR ($CDCl_3$, 125 MHz) δ = 134.3, 131.9, 129.8, 122.3, 54.0. IR (neat) = υ (cm^{-1}) 2927; 2093; 1488; 1282; 1246; 1012; 791.

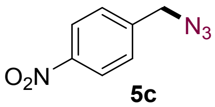

5c

1-(Azidomethyl)-4-nitrobenzene **5c**:[9] Purified by preparative TLC (hexane/ethyl acetate = 85:15); Yield: 0.0217 g (70%); colorless oil. ^{1}H NMR ($CDCl_3$, 500 MHz) δ (ppm) = δ 8.25 (d, J = 8.8 Hz, 2H); 7.50 (d, J = 8.8 Hz, 2H); 4.51 (s, 2H). ^{13}C{^{1}H} NMR ($CDCl_3$, 125 MHz) δ = 13C NMR (126 MHz, CDCl3) δ 147.7, 142.7, 128.6, 124.0, 53.7. IR (neat) = υ (cm^{-1}) 3081; 2097; 1516; 1340; 1294; 1256.

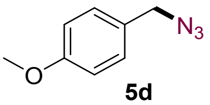

5d

1-(Azidomethyl)-4-methoxybenzene **5d**:[9] Purified by preparative TLC (hexane/ethyl acetate = 85:15); Yield: 0.0255 g (90%); colorless oil. ^{1}H NMR ($CDCl_3$, 500 MHz) δ (ppm) = δ 7.24 (d, J = 8.6 Hz, 2H); 6.91 (d, J = 8.6 Hz, 2H); 4.26 (s, 2H); 3.81 (s, 3H). ^{13}C{^{1}H} NMR ($CDCl_3$, 125 MHz) δ = 159.6, 129.7, 127.4, 114.2, 55.3, 54.4. IR (neat) = υ (cm^{-1}) 2935; 2091; 1611; 1512; 1243; 1175; 1032; 811.

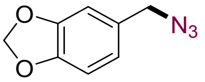

5e

5-(Azidomethyl)benzo[d][1,3]dioxole **5e**:[7] Purified by preparative TLC (hexane/ethyl acetate = 90:10); Yield: 0.0200 g (65%); yellowish oil. ^{1}H NMR ($CDCl_3$, 500 MHz) δ (ppm) = δ 6.81-6.76 (m, 3H); 5.97 (s, 2H); 4.22 (s, 2H). ^{13}C{^{1}H} NMR ($CDCl_3$, 125 MHz) δ = 148.0, 147.6, 129.0, 121.9, 108.7, 108.3, 101.2, 54.6. IR (neat) = υ (cm^{-1}) 2898; 2093; 2093; 1503; 1252; 1037; 927; 808.

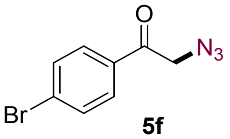

5f

2-Azido-1-(4-bromophenyl)ethan-1-one **5f**:[10] Purified by preparative TLC (hexane/ethyl acetate = 85:15); Yield: 0.0015 g (36%); colorless oil. ^{1}H NMR ($CDCl_3$, 500 MHz) δ (ppm) = δ 7.78 (d, J = 8.7 Hz, 2H); 7.65 (d, J = 8.7 Hz, 2H); 4.52 (s, 2H). ^{13}C{^{1}H} NMR ($CDCl_3$, 125 MHz) δ = 192.3, 133.1, 132.4, 129.5, 129.4, 54.8. IR (neat) = υ (cm^{-1}) 3086; 2904; 2098; 1692; 1293; 1215; 835.

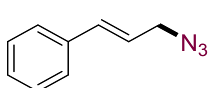

5g

(E)-(3-Azidoprop-1-en-1-yl)benzene **5g**:[7] Purified by preparative TLC (hexane/ethyl acetate = 90:10); Yield: 0.0152 g (55%); yellowish oil. ^{1}H NMR ($CDCl_3$, 500 MHz) δ (ppm) = δ 7.41-7.39 (m, 2H); 7.35-7.32 (m, 2H); 7.29-7.26 (m, 1H); 6.65 (d, J = 15.7 Hz, 1H); 6.24 (dt, J = 15.5 Hz and 6.6 Hz, 1H); 3.94 (d, J = 6.6 Hz, 2H). ^{13}C{^{1}H} NMR ($CDCl_3$, 125 MHz) δ = 136.0, 134.5, 128.6, 128.2, 126.6, 122.4, 53.0. IR (neat) = υ (cm^{-1}) 3028; 2926; 2093; 1233; 965.

General procedure for Gram-scale reaction:

A 10.0 mL round-bottomed glass vial was added the benzyl bromide **3a** (4.1 mmol, 70 mg), **P[5]-TePh** (0.041 mmol, 188.5 mg; 1 mol%) and water (23.6 mL). The resulting mixture was stirred at room temperature for 5 minutes. After this, NaCN (8.2 mmol, 401.8 mg) was added, and the mixture was stirred for an additional 12 hours. After that, the reaction mixture was extracted with ethyl acetate (3x 45.0 mL). The combined organic layers were dried over Na_2SO_4 and concentrated under vacuum. The residue was purified by preparative TLC using hexane/ethyl acetate (90:10) as the eluent, to afford the desired product 4a in 91% yield (3.7 mmol, 436 mg).

General procedure for recovery and reuse of the catalyst P[5]-TePh:

A 10.0 mL round-bottomed glass vial was added the appropriate benzyl bromide **3a** (0.174 mmol), **P[5]-TePh** (0.00174 mmol, 8.0 mg; 1 mol%) and water (1.0 mL). The resulting mixture was stirred at room temperature for 5 minutes. After this, NaCN (0.358 mmol, 17.5 mg) was added, and the mixture was stirred for an additional 12 hours. After that, the reaction mixture was extracted with ethyl acetate (3x 15.0 mL). The combined organic layers were dried over Na_2SO_4 and concentrated under vacuum. The crude was washed with hexanes (3 x 5 mL) to separate the catalyst **P[5]-TePh** from the crude product **4a**. Then, the catalyst is dried under vacuum and used directly in another cycle.

Experiments to detect the interactions between substrates and P[5]-TePh

^{1}H Nuclear Magnetic Resonance Analyses:

A test tube was charged with substrate **3i** (4.6 mg, 0.0169 mmol). P[5]-TePh (10.0 mg, 0.0039 mmol) and $CDCl_3$ (1 mL). After stirring for 15 minutes at 25 ºC, the solution was then transferred to an NMR tube and analyzed by ^{1}H NMR.

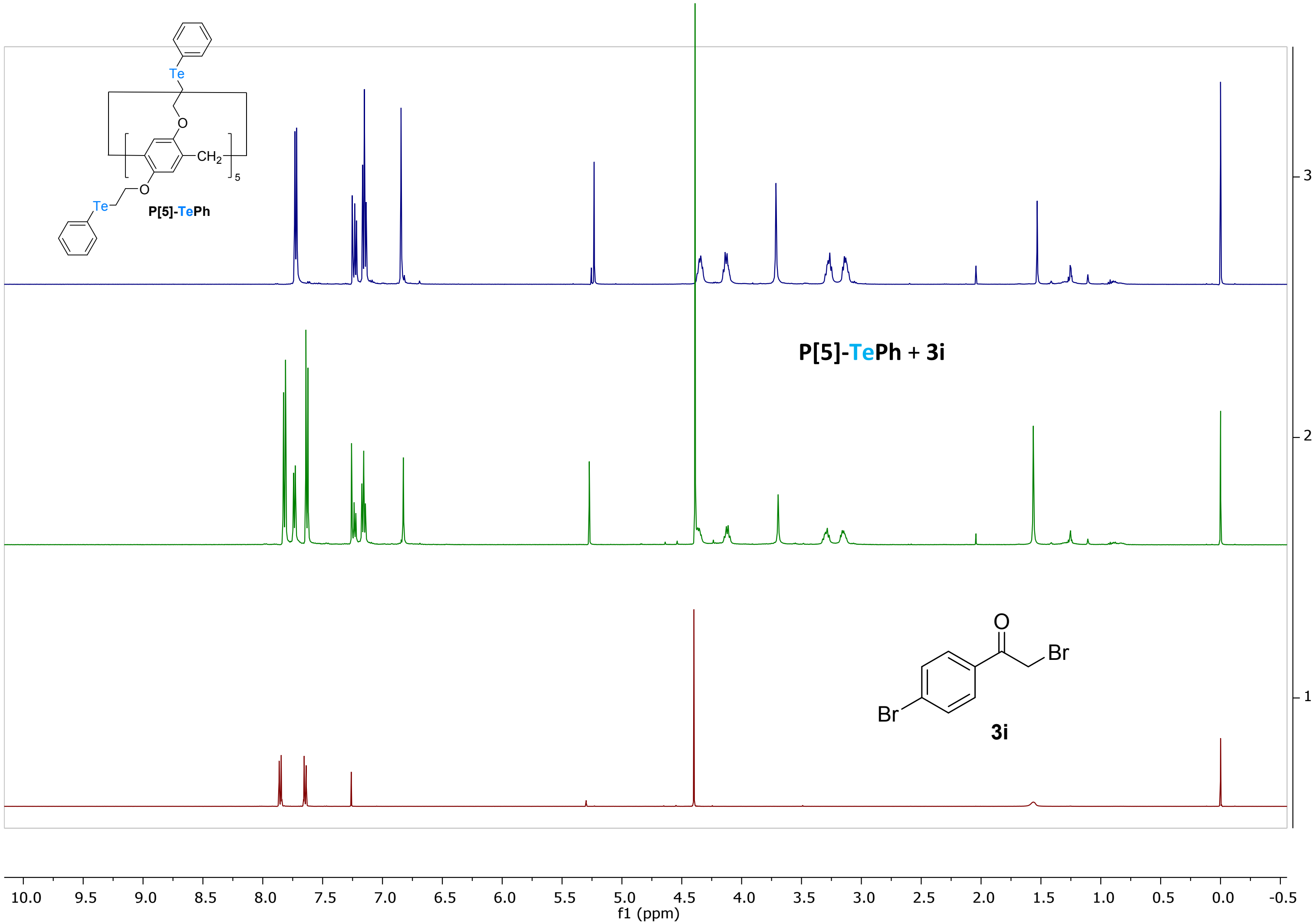

Figure S1. Stacked ^{1}H NMR spectra of **3i** (red), **P[5]-TePh** (blue) and **3i** + **P[5]-TePh** (green).

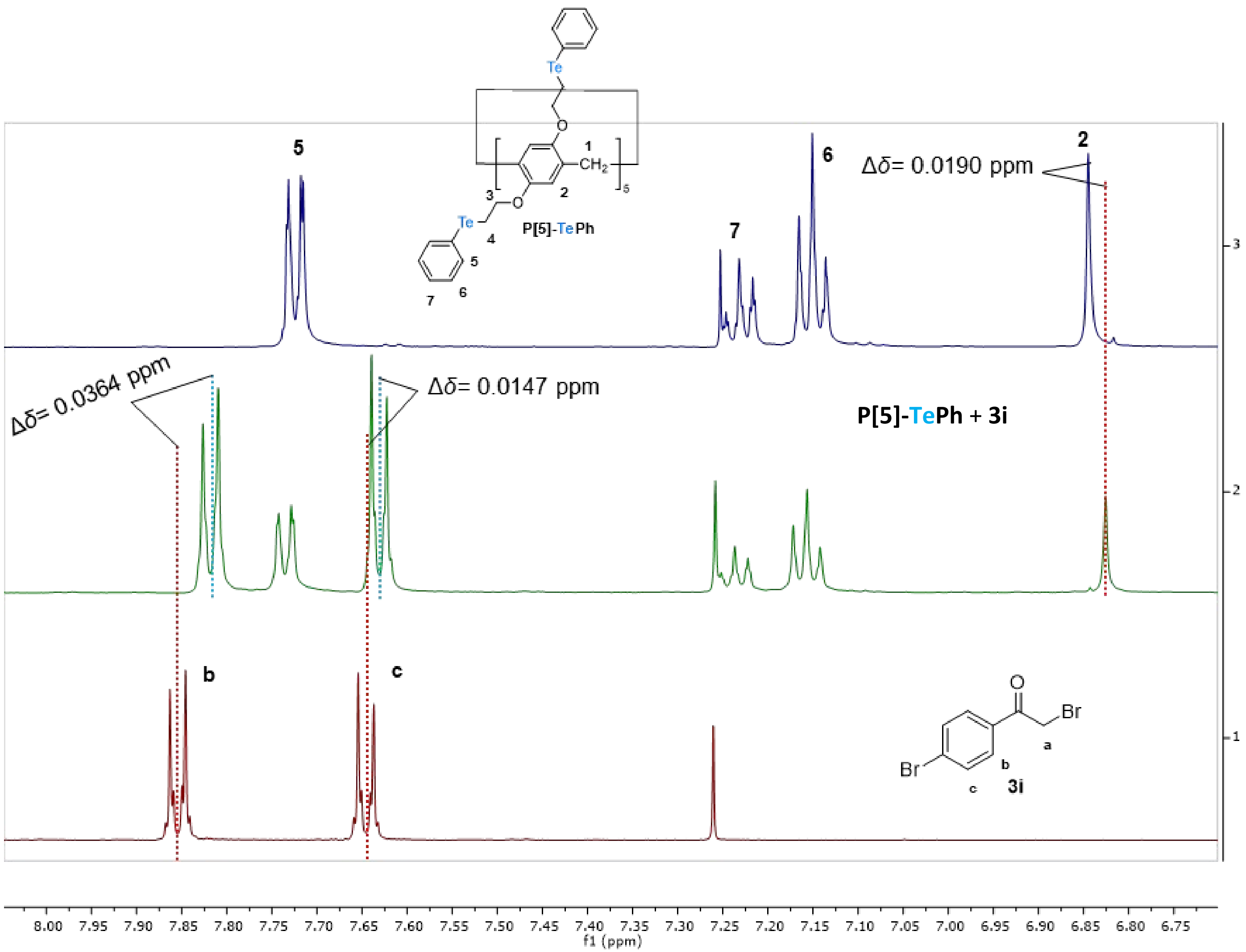

Figure S2: Partial ^{1}H NMR of **3i** (red), **P[5]-TePh** (blue) and **3i** + **P[5]-TePh** (green).

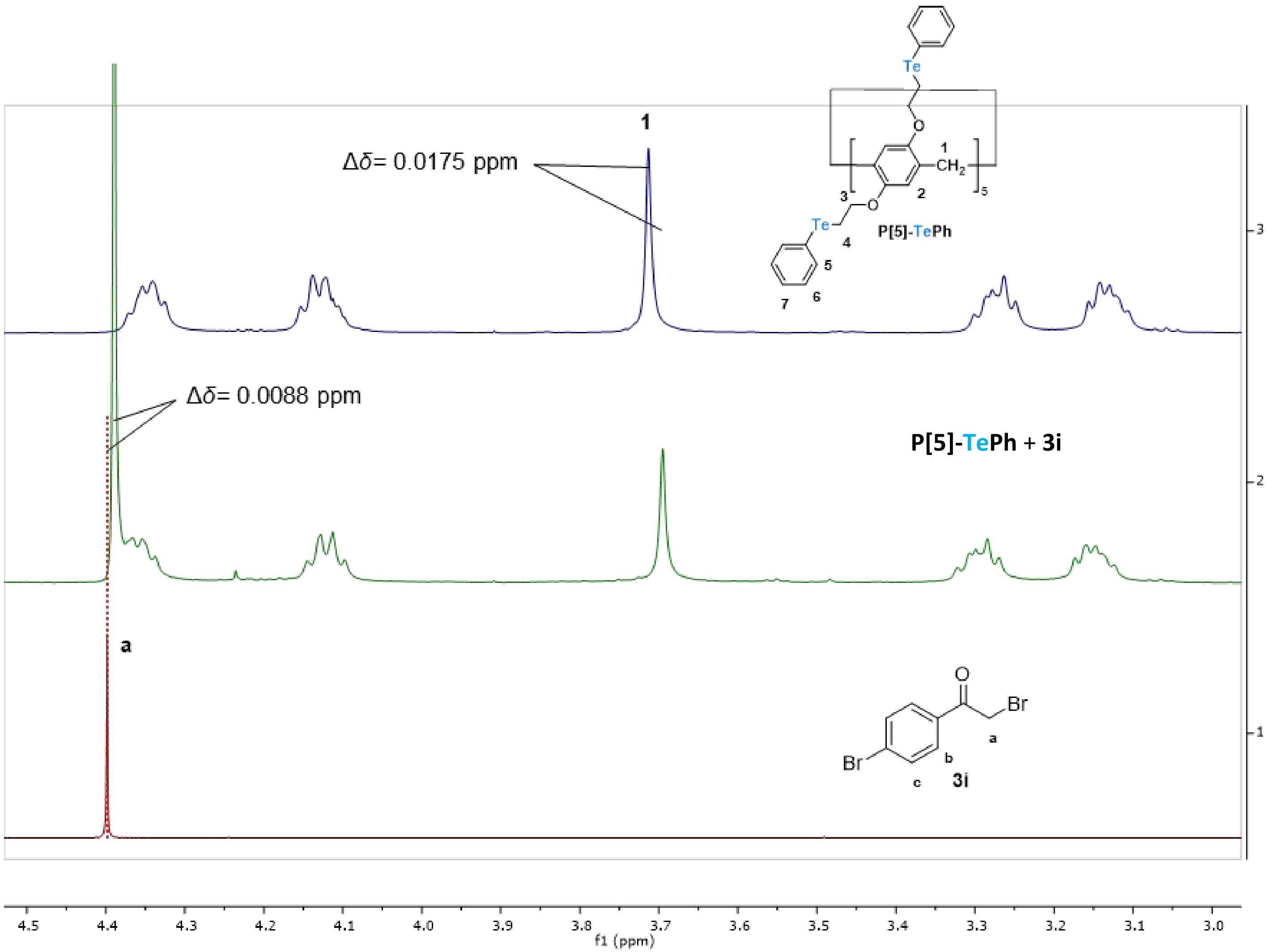

Figure S3: Partial ^{1}H NMR of **3i** (red), **P[5]-TePh** (blue) and **3i** + **P[5]-TePh** (green).

Progress of the reaction

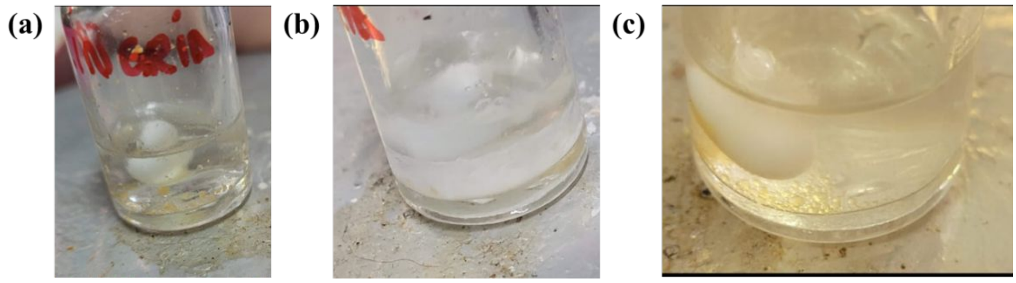

Figure S4. Progress of the reaction. **(a)** after addition of reagents, **(b)** during reaction **(c)** after the reaction is over.

References

[1] da Silva, A. F. M.; da Costa, N. M.; Fernandes, T. S.; Bessa, I. A. A.; D'Amato, D. L.; Senna, C. A.; Lohan-Codeço, M.; Nascimento, V.; Jr, P. A.; Archanjo, B. S.; Pinto, L. F. R.; dos Santos, T. C.; Ronconi, C. M. Responsive Supramolecular Devices Assembled from Pillar[5]arene Nanogate and Mesoporous Silica for Cargo Release *ACS Appl. Nano Mater* **2022**, *5*, 10, 13805-13819. DOI: 10.1021/acsanm.2c01408[2] Nierengarten, I.; Guerra, S.; Holler, M.; Karmazin-Brelot, L.; Barberá, J.; Deschenaux, R.; Nierengarten, J.-F. Macrocyclic Effects in the Mesomorphic Properties of Liquid-Crystalline Pillar[5]- and Pillar[6]arenes *Eur. J. Org, Chem.* **2013**, 3675-3684. DOI: 10.1002/ejoc.201300356.

[3] Zhou, Y..; Jie, K.; Huang, F. A redox-responsive selenium-containing pillar[5]arene-based macrocyclic amphiphile: synthesis, controllable self-assembly in water, and application in controlled release *Chem. Commun.* **2017**, *53*, 8364-8367. DOI: 10.1039/C7CC04779G.

[4] Martins, N. S.; Ángel, A. Y. B.; Anghinoni, J. M.; Lenardão, E. J.; Barcellos, T.; Alberto, E. E. From Stoichiometric Reagents to Catalytic Partners: Selenonium Salts as Alkylating Agents for Nucleophilic Displacement Reactions in Water *Adv. Synth. Catal.* **2022**, *364*, 87-93. DOI: 10.1002/adsc.202100797.

[5] Zhang, J.-Q.; Liu, J.; Hu, D.; Song, J.; Zhu, G.; Ren, H. Rapid and Simple Access to α-(Hetero)arylacetonitriles from Gem-Difluoroalkenes *Org. Lett.* **2022**, *24*, 786-790. DOI: 10.1021/acs.orglett.1c04336.

[6] Röckl, J. L.; Imada, Y.; Chiba, K.; Franke, R.; Waldvogel, S. R. Dehydrogenative Anodic Cyanation Reaction of Phenols in Benzylic Positions *ChemElectroChem* **2019**, *6*, 4184-418. DOI: 10.1002/celc.201801727.

[7] Lenstra, D. C.; Lenting, P. E.; Mecinović, J. Sustainable organophosphorus-catalysed Staudinger reduction *Green Chem.* **2018**, *20*, 4418-4422. DOI: 10.1039/C8GC02136H.

[8] Chen, X.; Peng, Y.; Yu, W.; Zhang, X.; Shao, X.; Xu, X.; Li, Z. Condition-Based Selective Synthesis of 3,4,5-Trisubstituted Isoxazoline N-oxides, 4,5-Dihydroisoxazoles and Isoxazoles *ChemistrySelect* **2018**, *3*, 6344-6348. DOI: 10.1002/slct.201800839.

[9] Bao, M; Lu, W.; Su, H.; Qiu, L.; Xu, X. A convergent formal [4 + 2] cycloaddition of 1,6-diynes and benzyl azides: construction of spiro-polyheterocycles *Org. Biomol. Chem.* **2018**, *16*, 3258-3265. DOI: 10.1039/C8OB00735G.

[10] Panday, P.; Garg, P.; Singh, A. Manganese-Dioxide-Catalyzed Trifluoromethylation and Azidation of Styrenyl Olefins via Radical Intermediates *Asian J. Org. Chem.* **2018**, *7*, 111-115. 10.1002/ajoc.201700508.

Selected Spectra

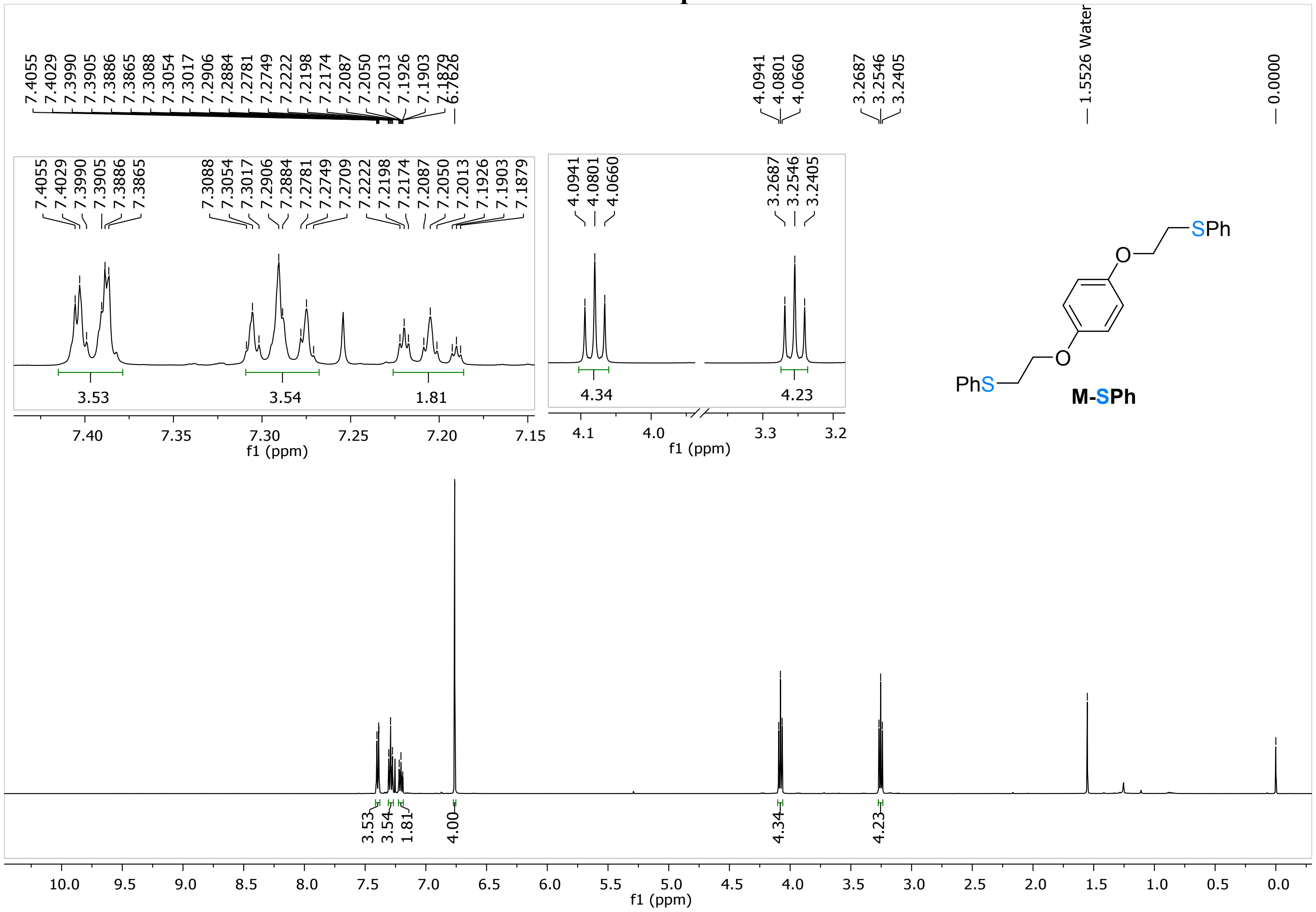

Figure S5. ^{1}H NMR (500 MHz, $CDCl_3$) of the compound **M-SPh.**

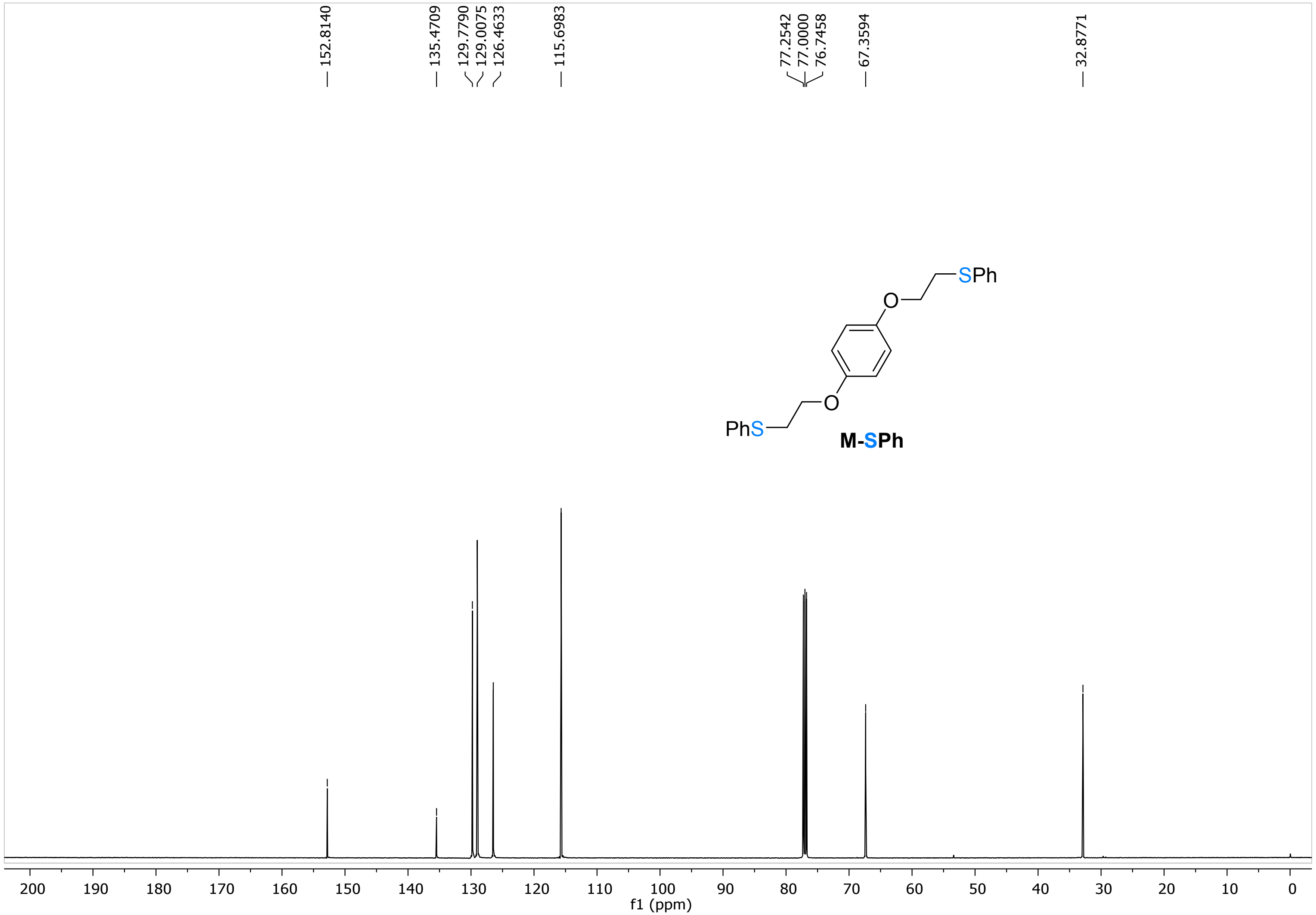

Figure S6. ^{13}C NMR (125 MHz, $CDCl_3$) of the compound **M-SPh.**

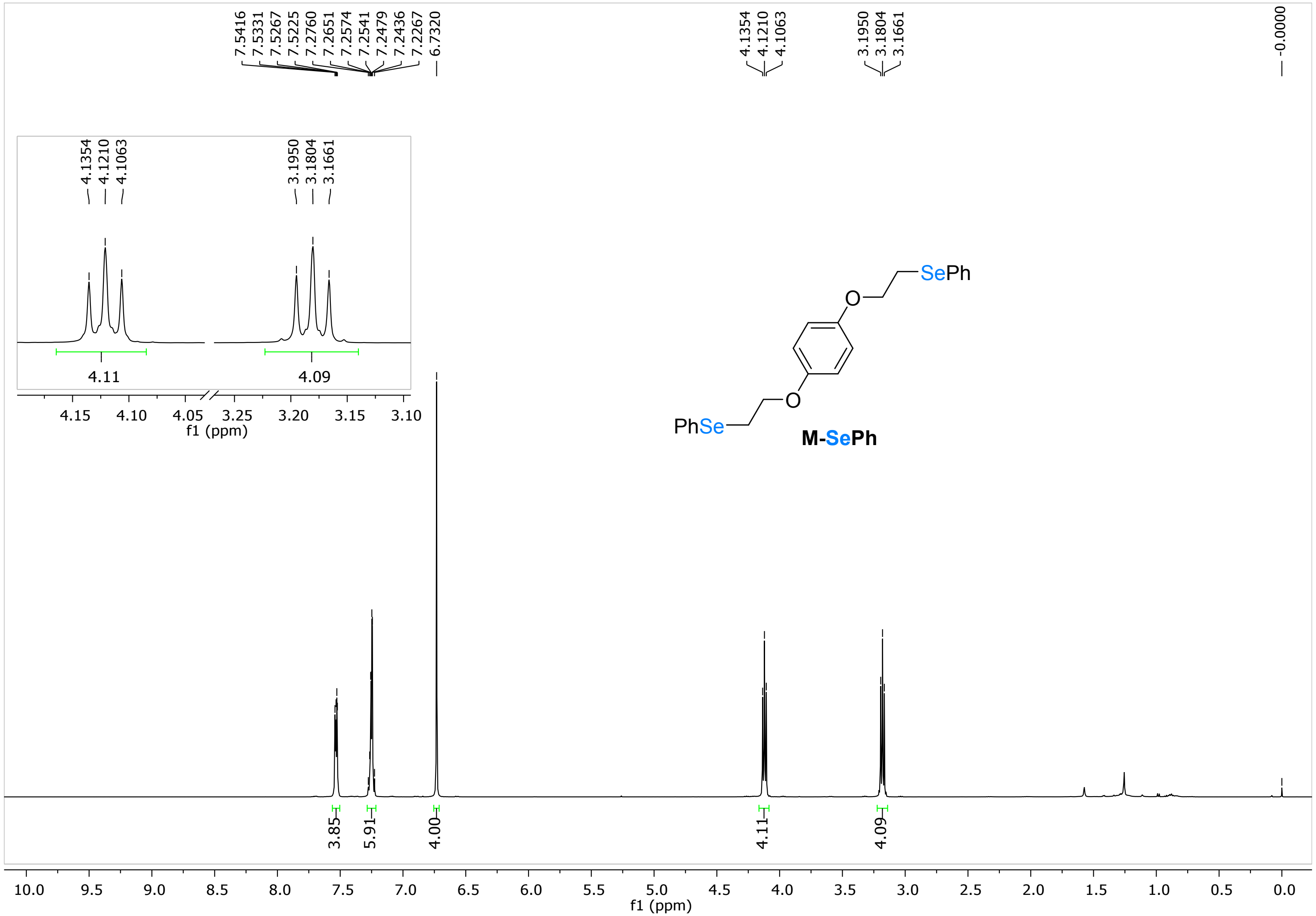

Figure S7. ^{1}H NMR (500 MHz, $CDCl_3$) of the compound **M-SePh.**

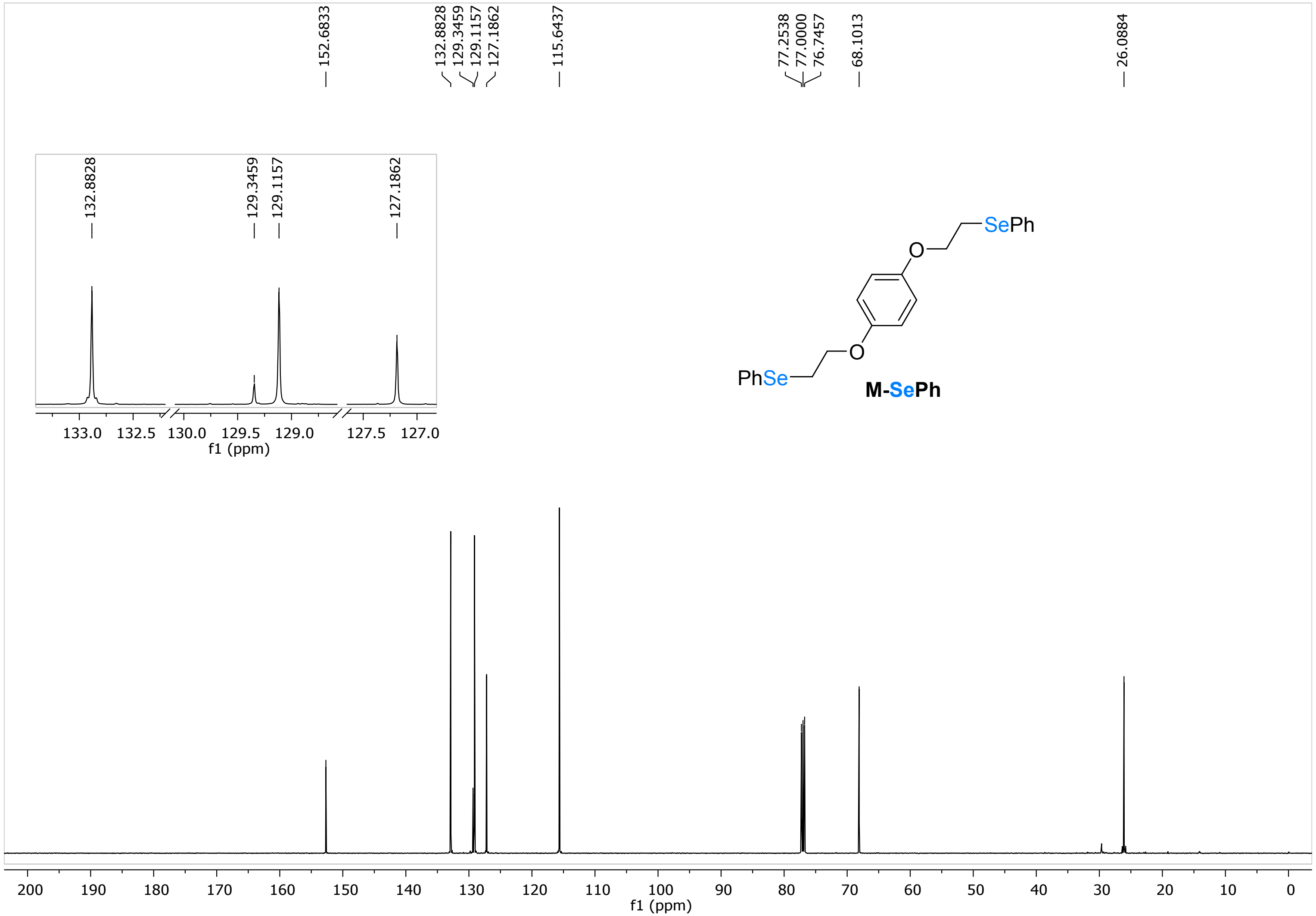

Figure S8. ^{13}C NMR (125 MHz, $CDCl_3$) of the compound **M-SePh.**

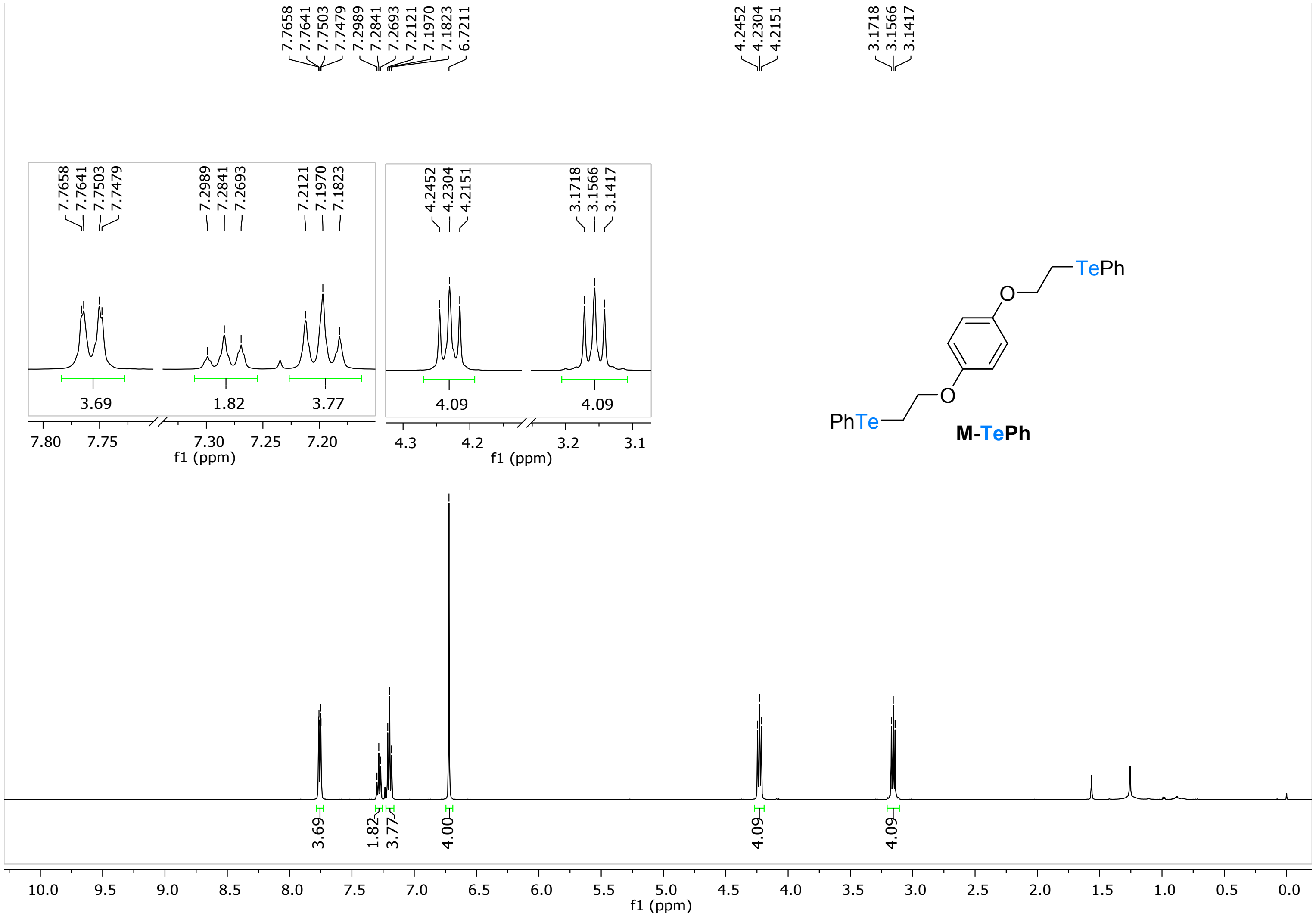

Figure S9. ^{1}H NMR (500 MHz, $CDCl_3$) of the compound **M-TePh.**

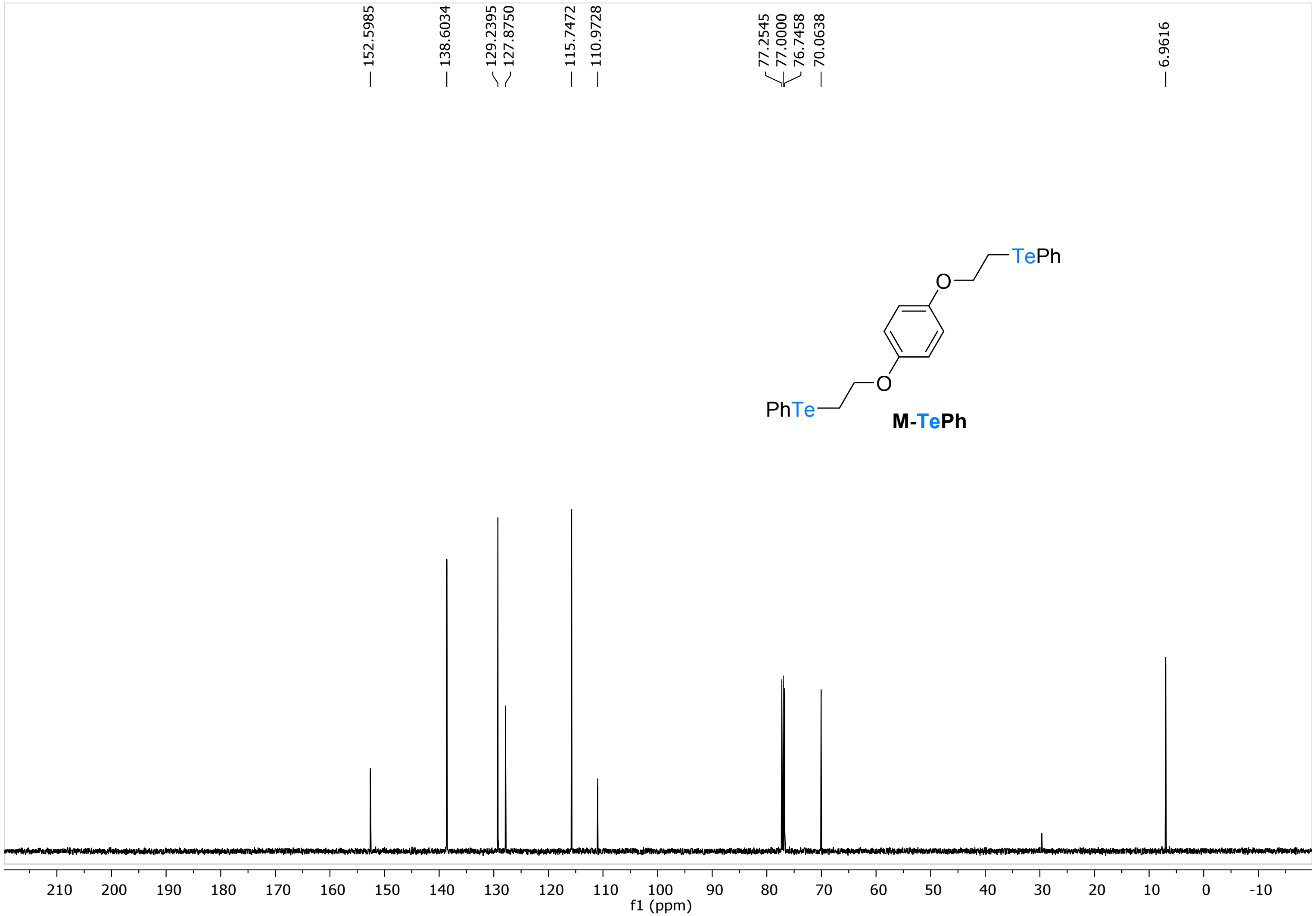

Figure S10. ^{13}C NMR (125 MHz, $CDCl_3$) of the compound **M-TePh.**

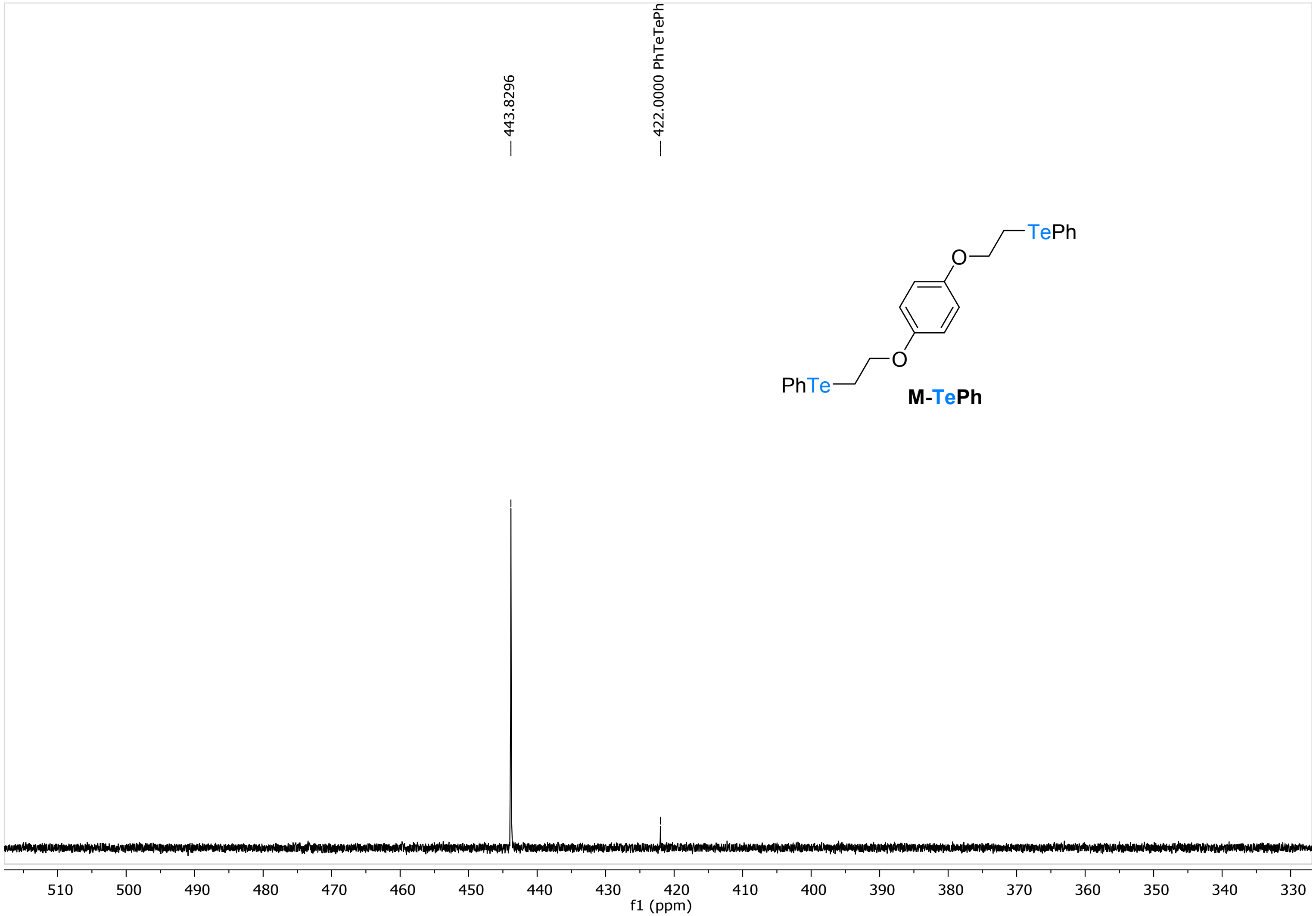

Figure S11. ^{125}Te NMR (158 MHz, $CDCl_3$) of the compound **M-TePh.**

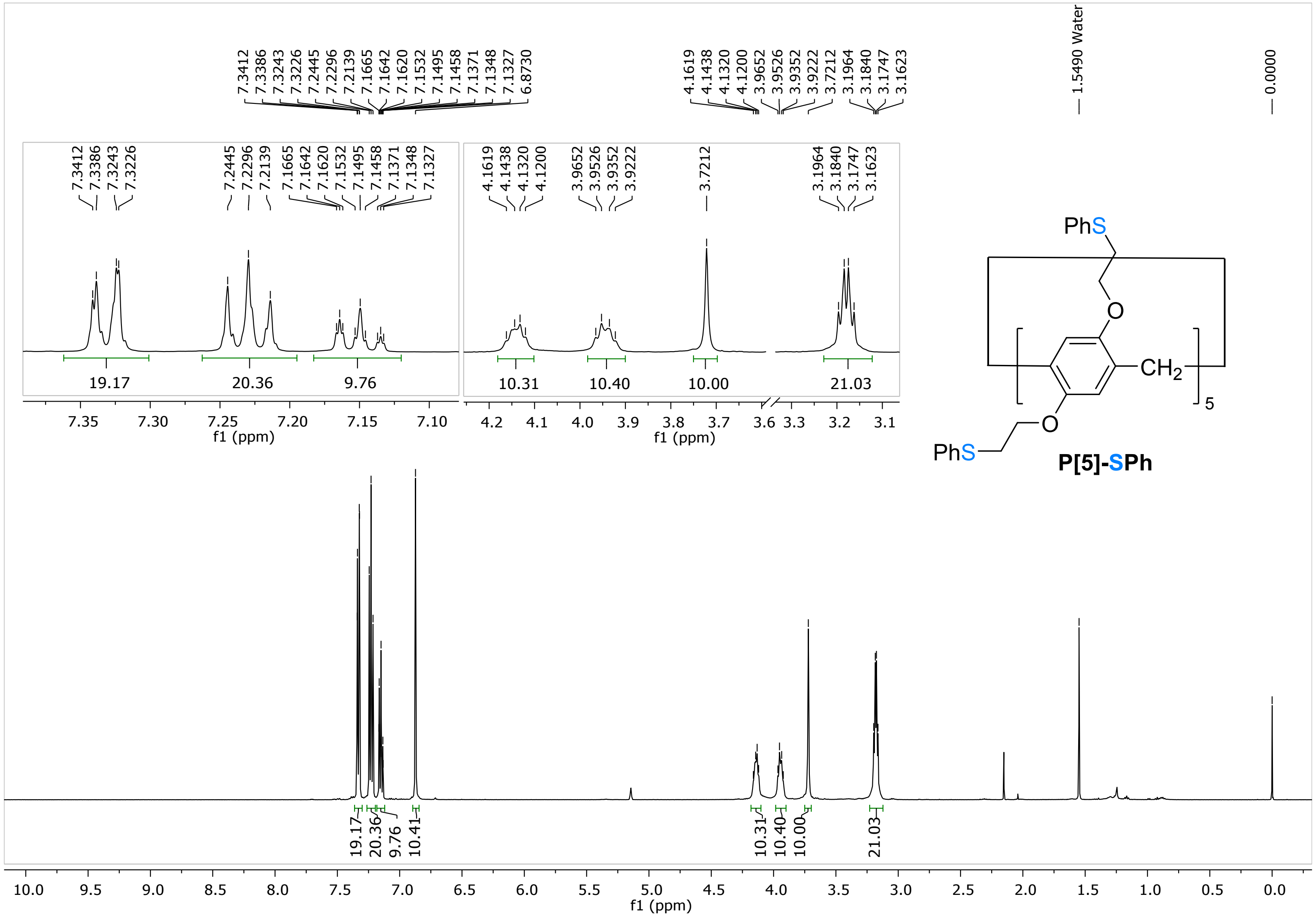

Figure S12. ^{1}H NMR (500 MHz, $CDCl_3$) of the compound **P[5]-SPh.**

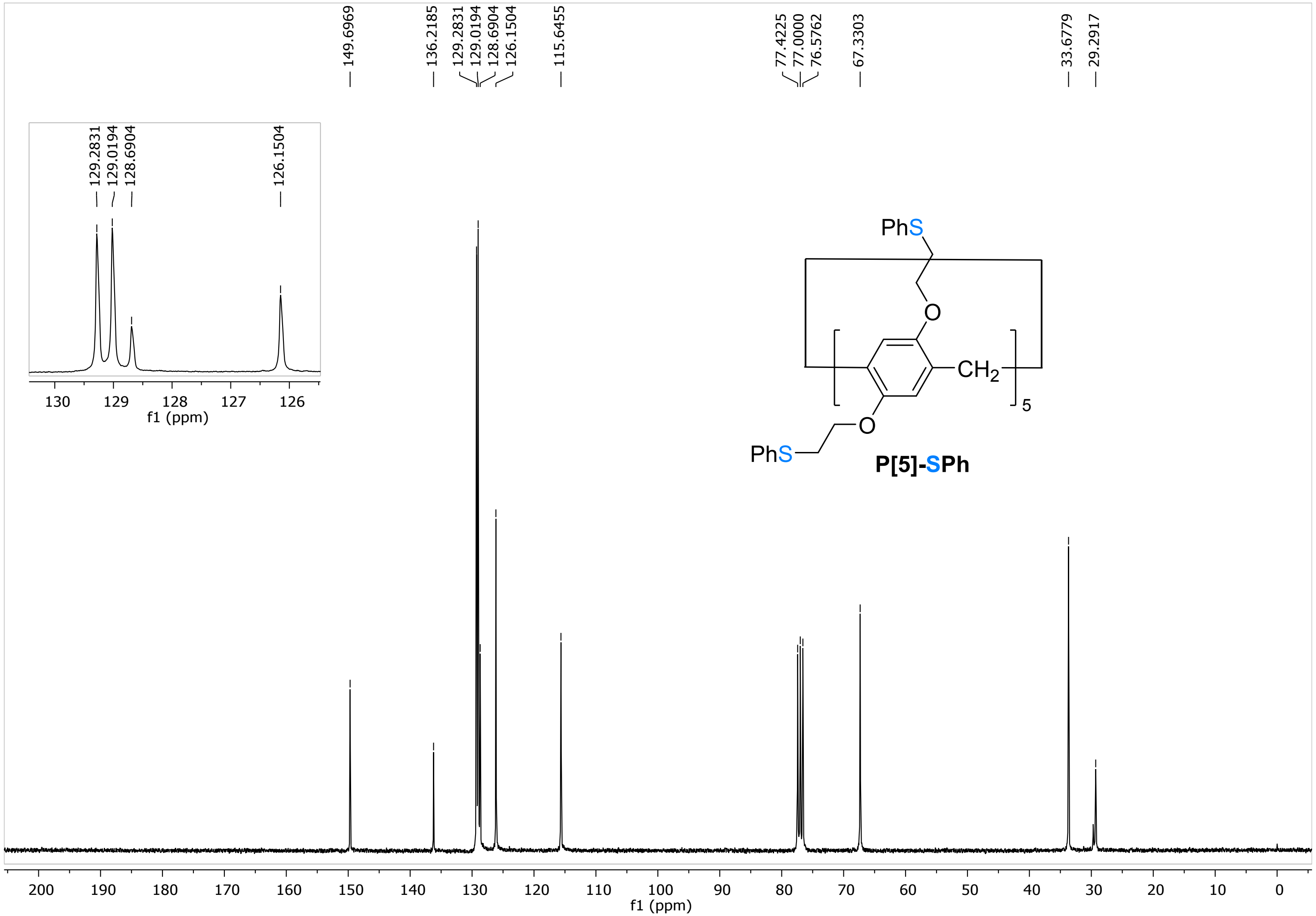

Figure S13. ^{13}C NMR (125 MHz, $CDCl_3$) of the compound **P[5]-SPh.**

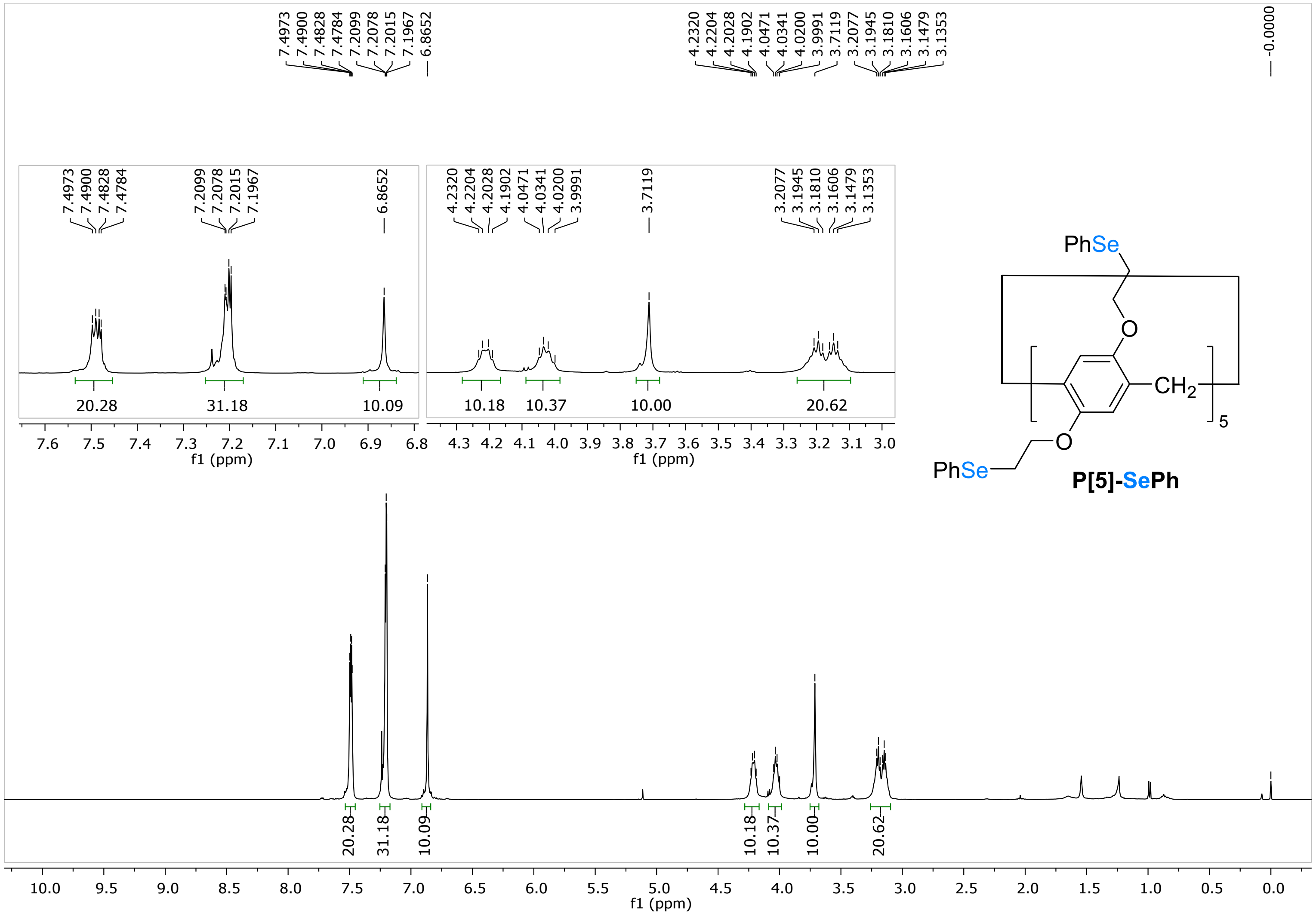

Figure S14. ^{1}H NMR (500 MHz, $CDCl_3$) of the compound **P[5]-SePh.**

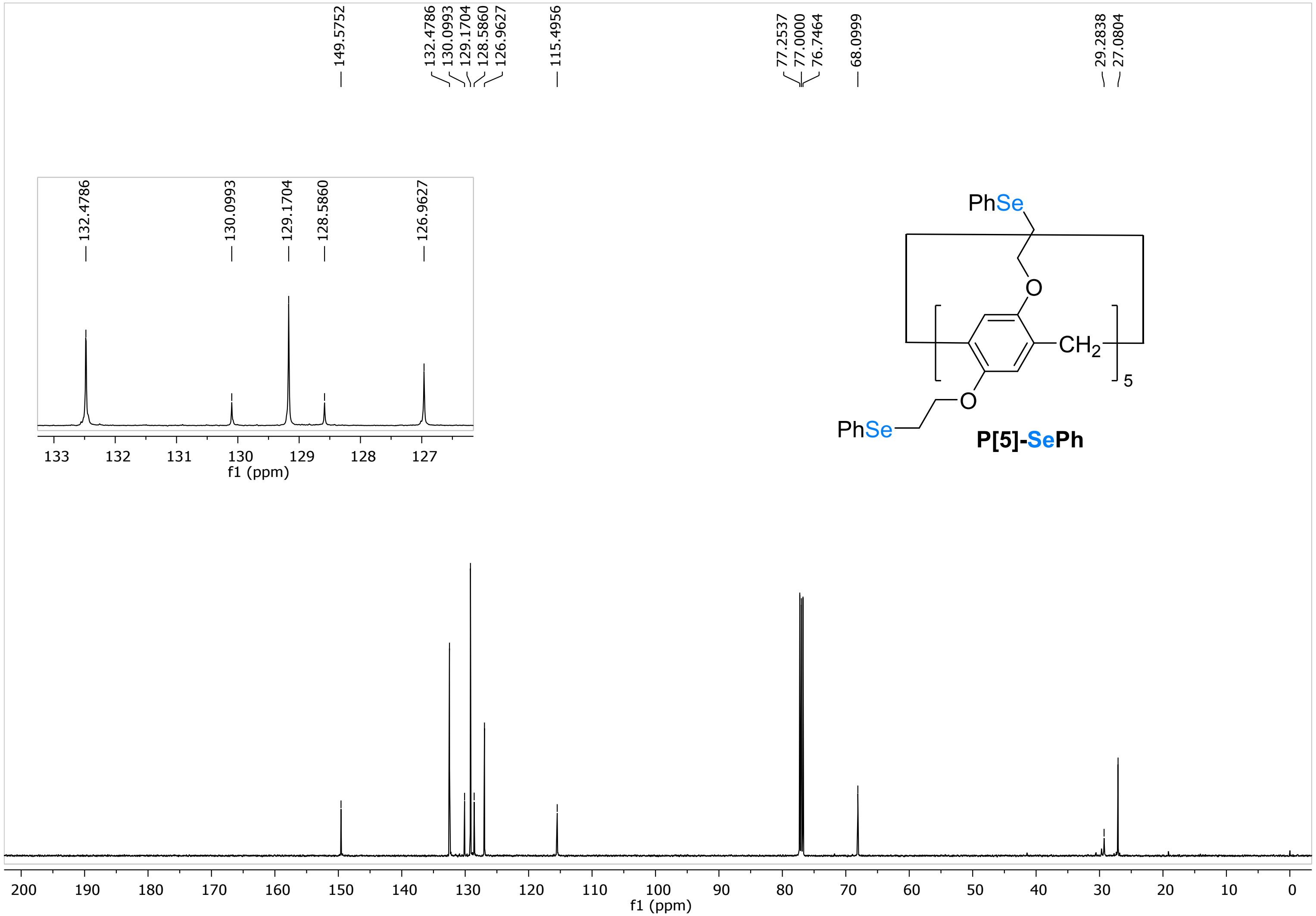

Figure S15. ^{1}H NMR (500 MHz, $CDCl_3$) of the compound **P[5]-SePh.**

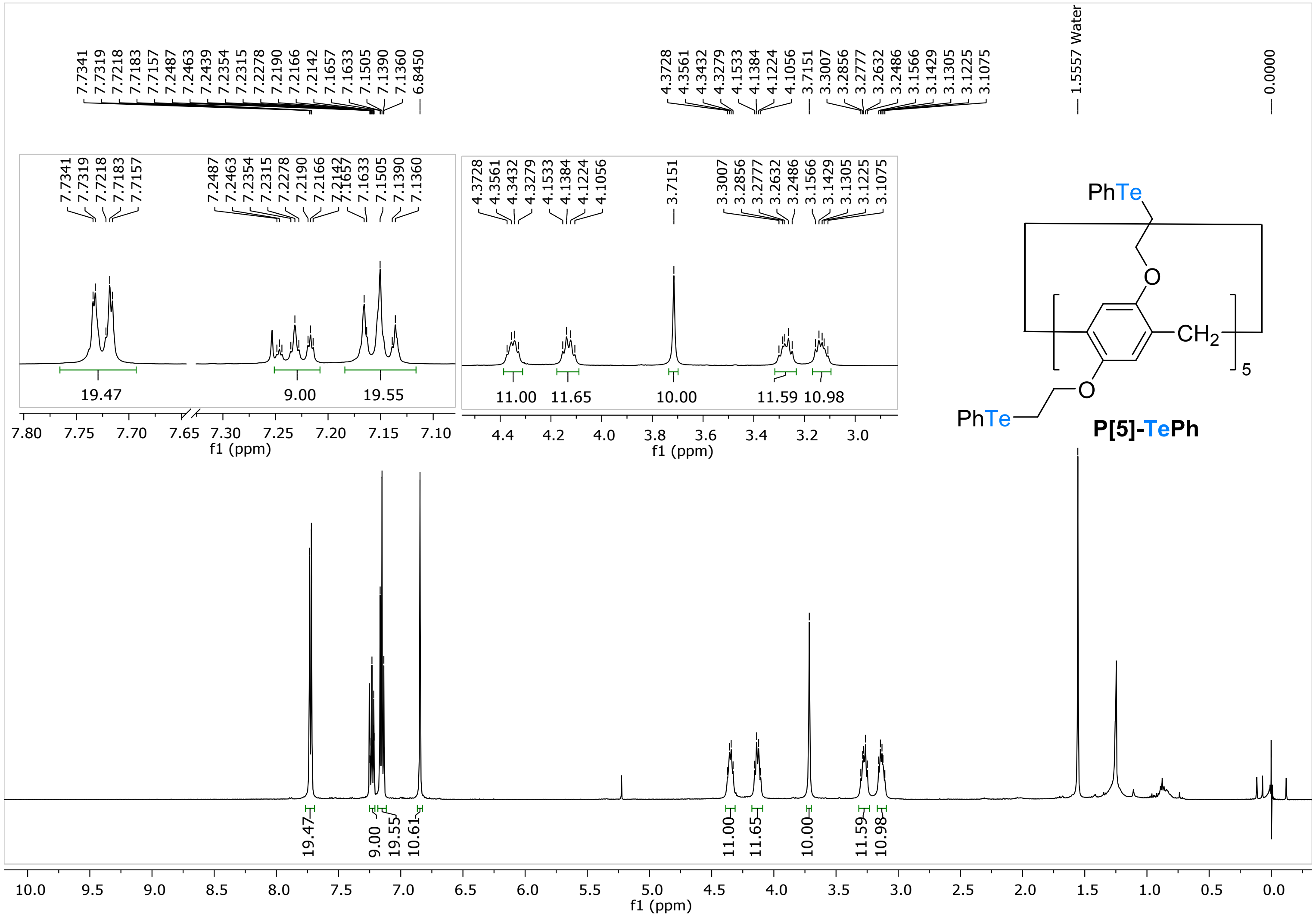

Figure S16. ^{1}H NMR (500 MHz, $CDCl_3$) of the compound **P[5]-TePh.**

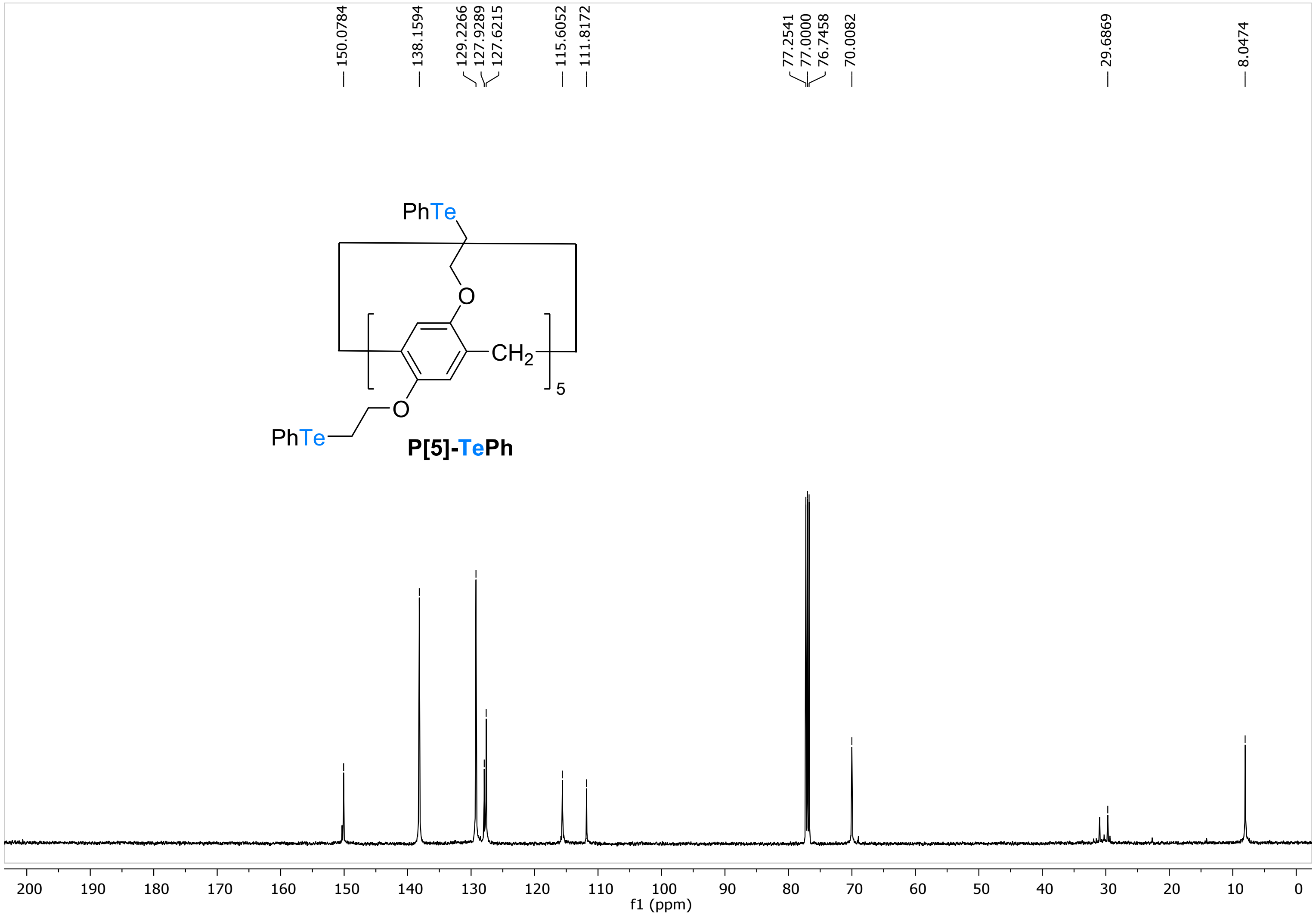

Figure S17. ^{13}C NMR (125 MHz, $CDCl_3$) of the compound **P[5]-TePh.**

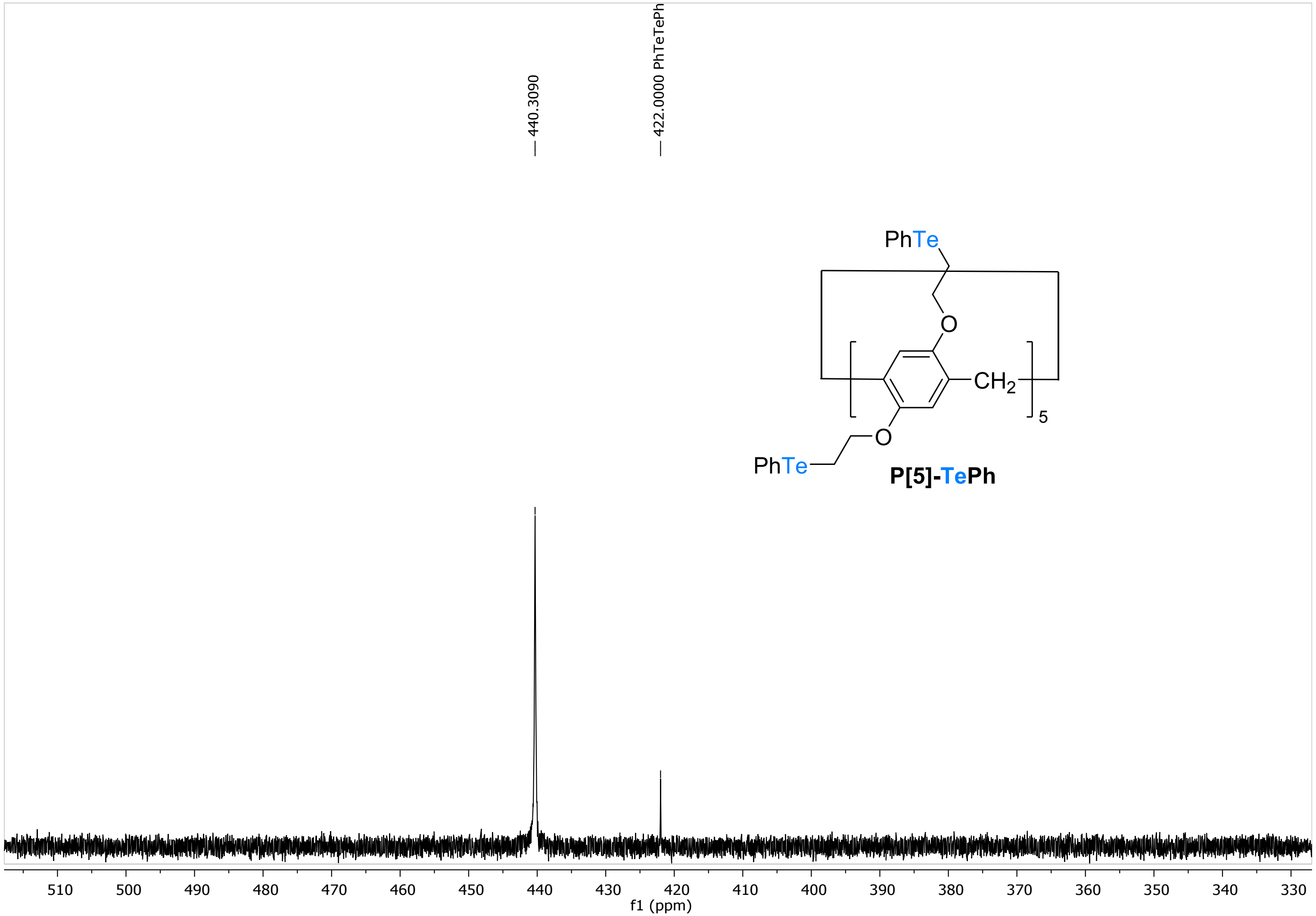

Figure S18. ^{125}Te NMR (158 MHz, $CDCl_3$) of the compound **P[5]-TePh.**

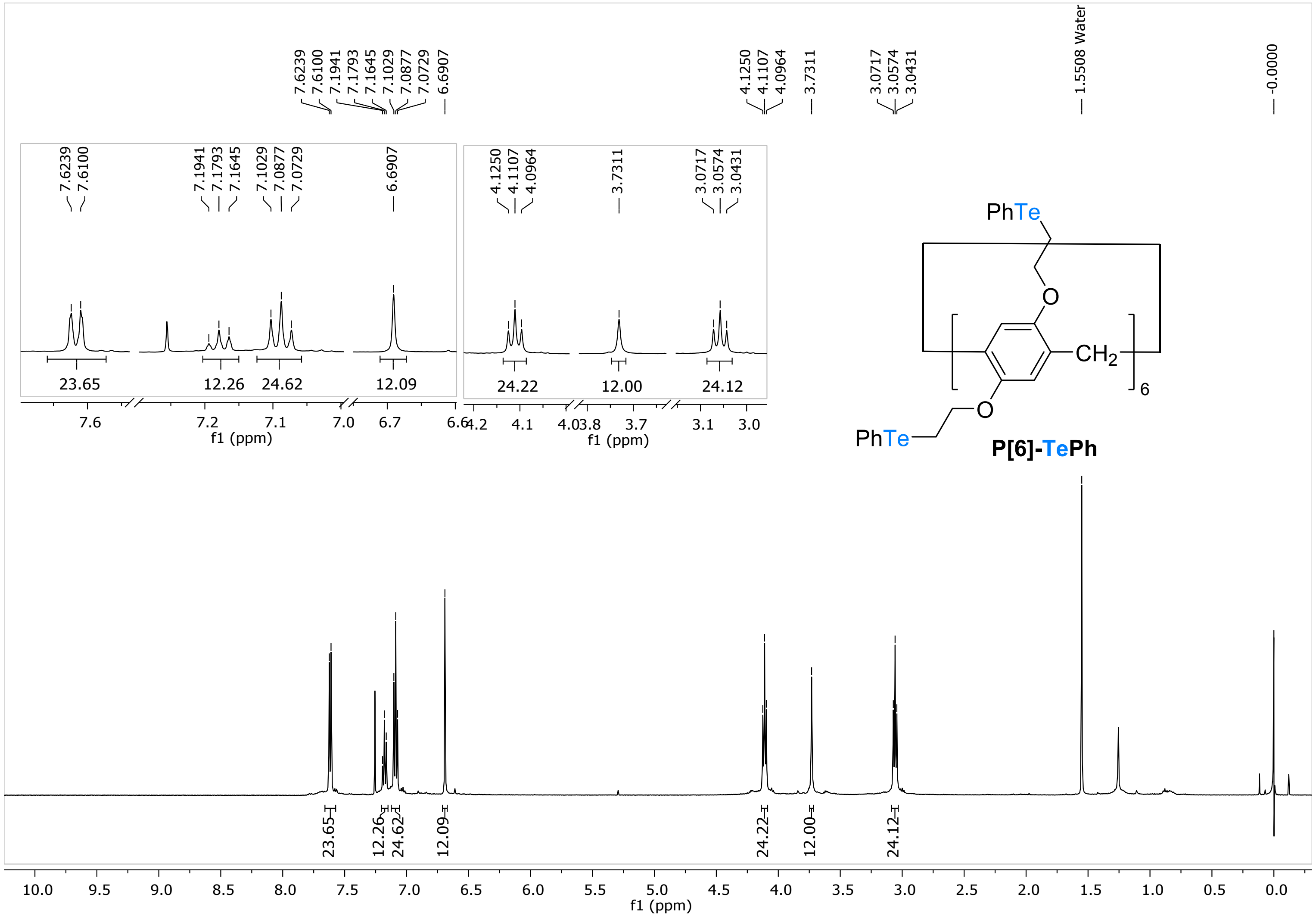

Figure S19. ^{1}H NMR (500 MHz, $CDCl_3$) of the compound **P[6]-TePh.**

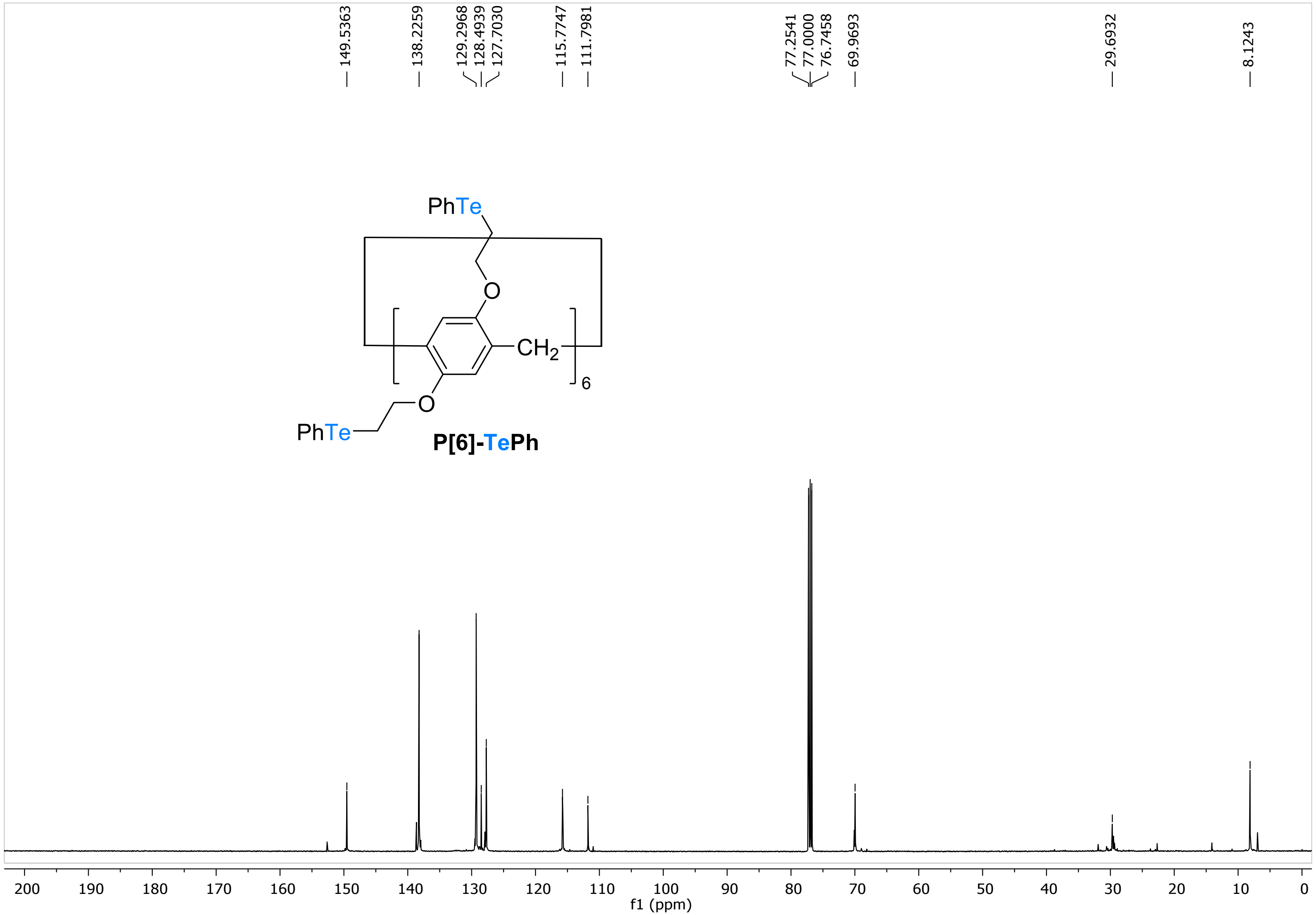

Figure S20. ^{13}C NMR (125 MHz, $CDCl_3$) of the compound **P[6]-TePh.**

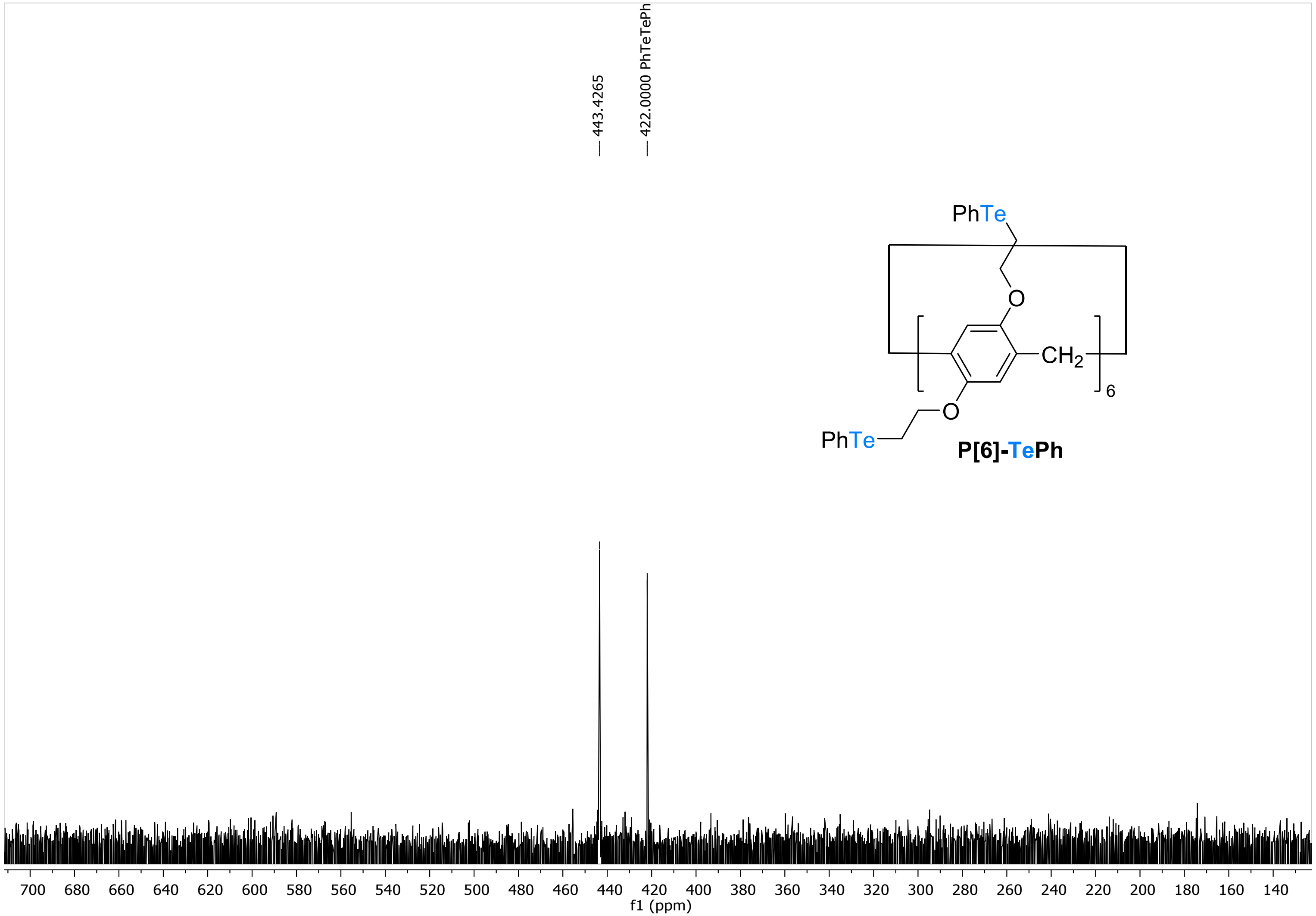

Figure S21. ^{125}Te NMR (158 MHz, $CDCl_3$) of the compound **P[6]-TePh.**

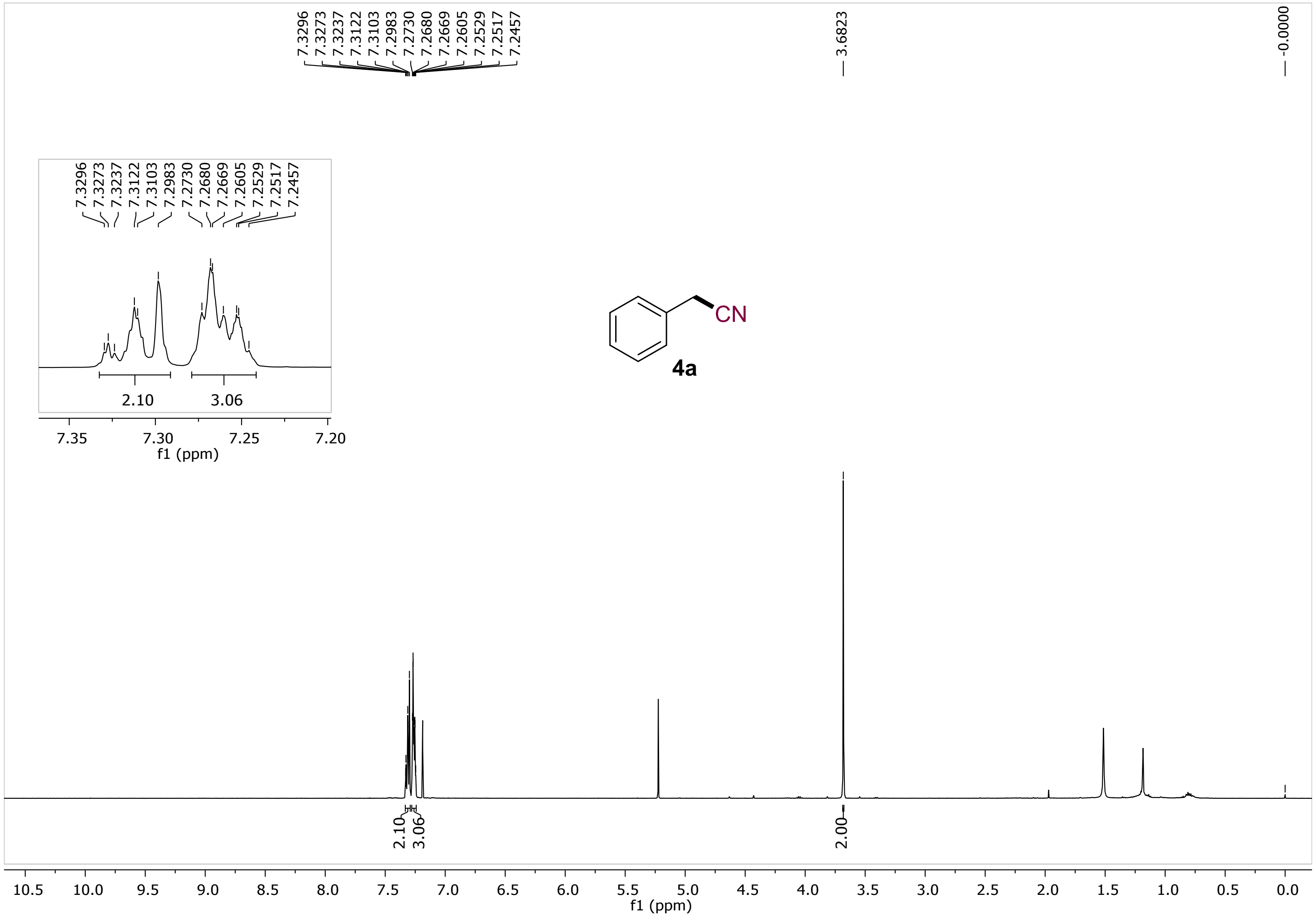

Figure S22. ^{1}H NMR (500 MHz, $CDCl_3$) of the compound **4a.**

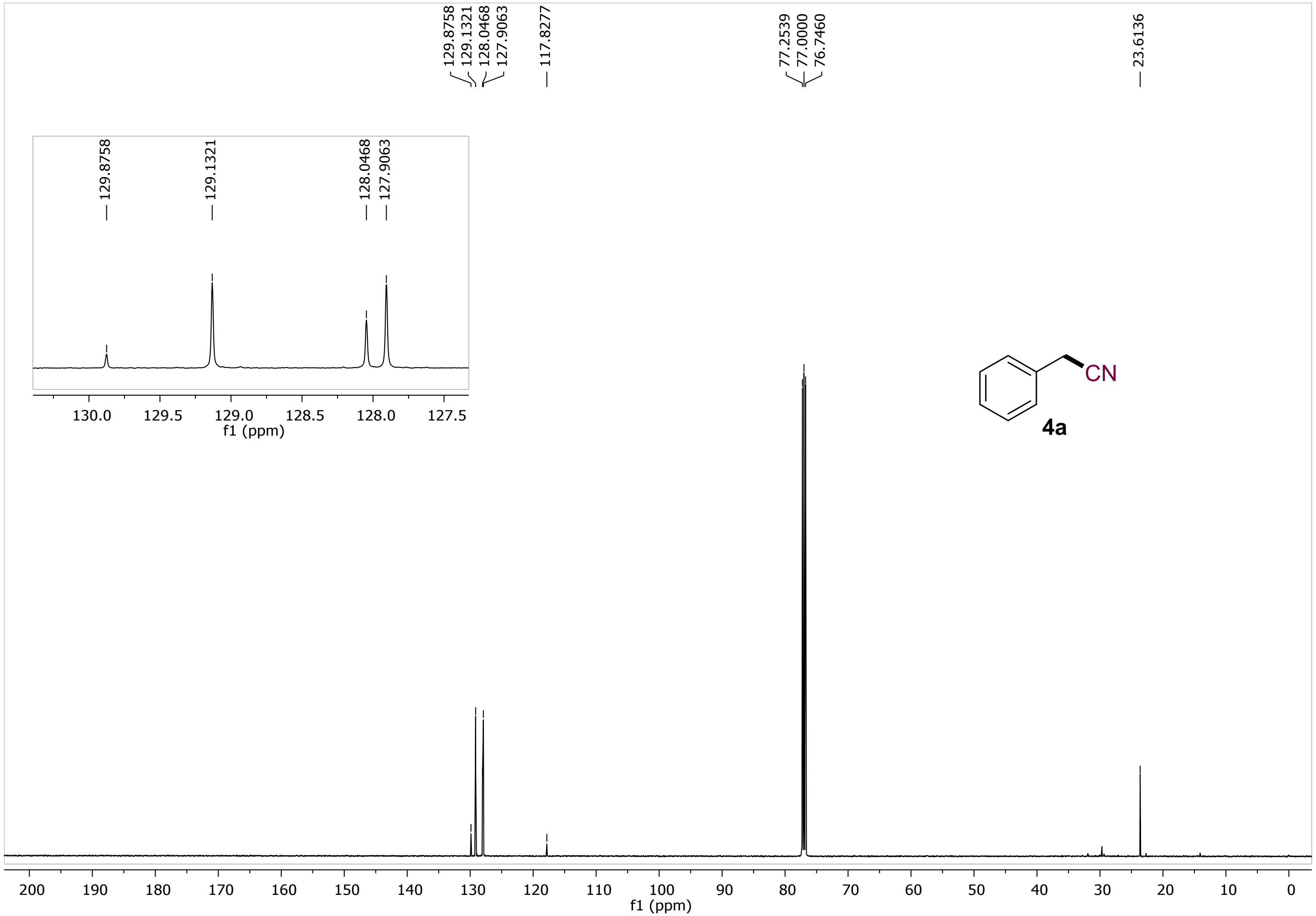

Figure S23. ^{13}C NMR (125 MHz, $CDCl_3$) of the compound **2a.**

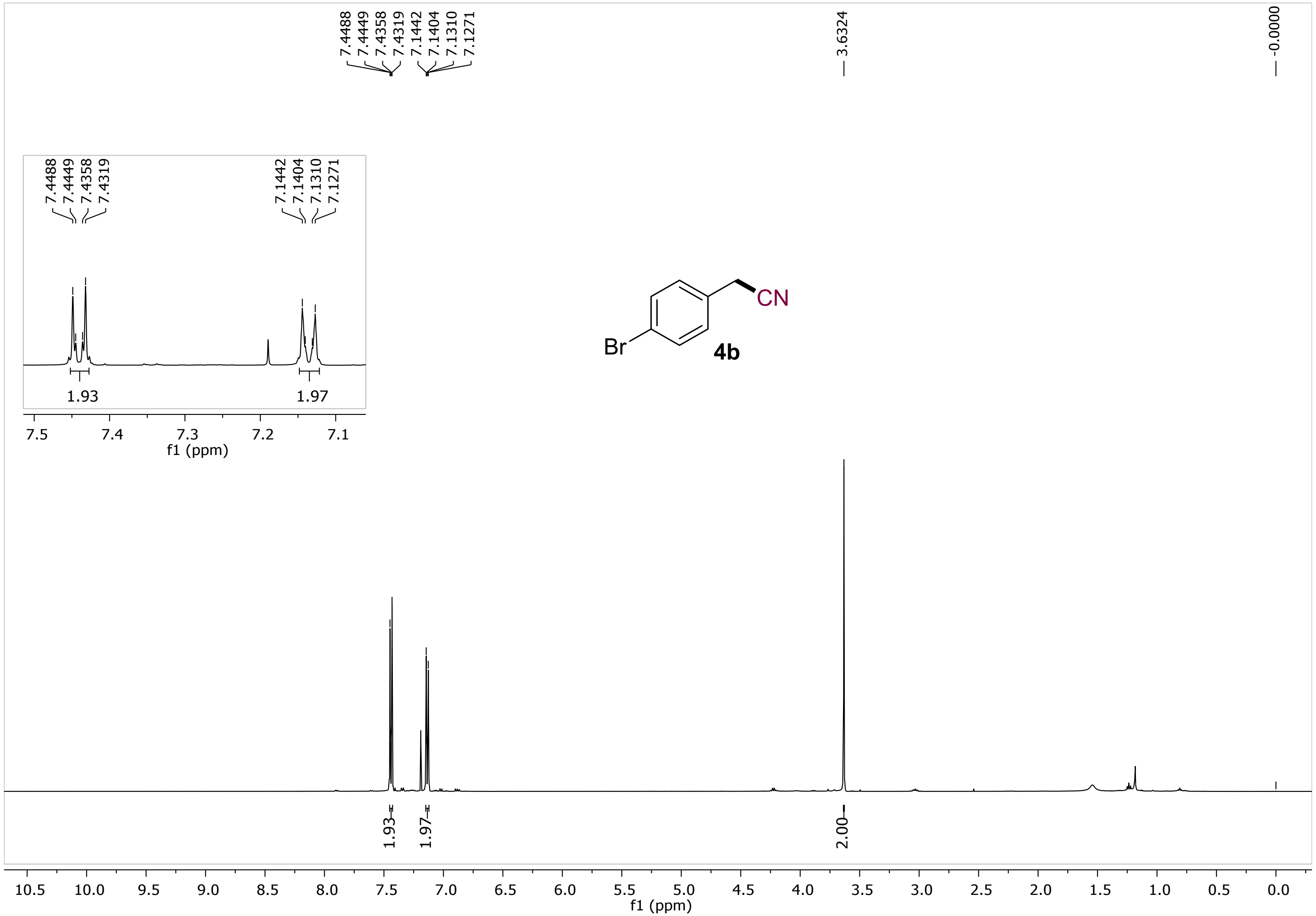

Figure S24. ^{1}H NMR (500 MHz, $CDCl_3$) of the compound **4b.**

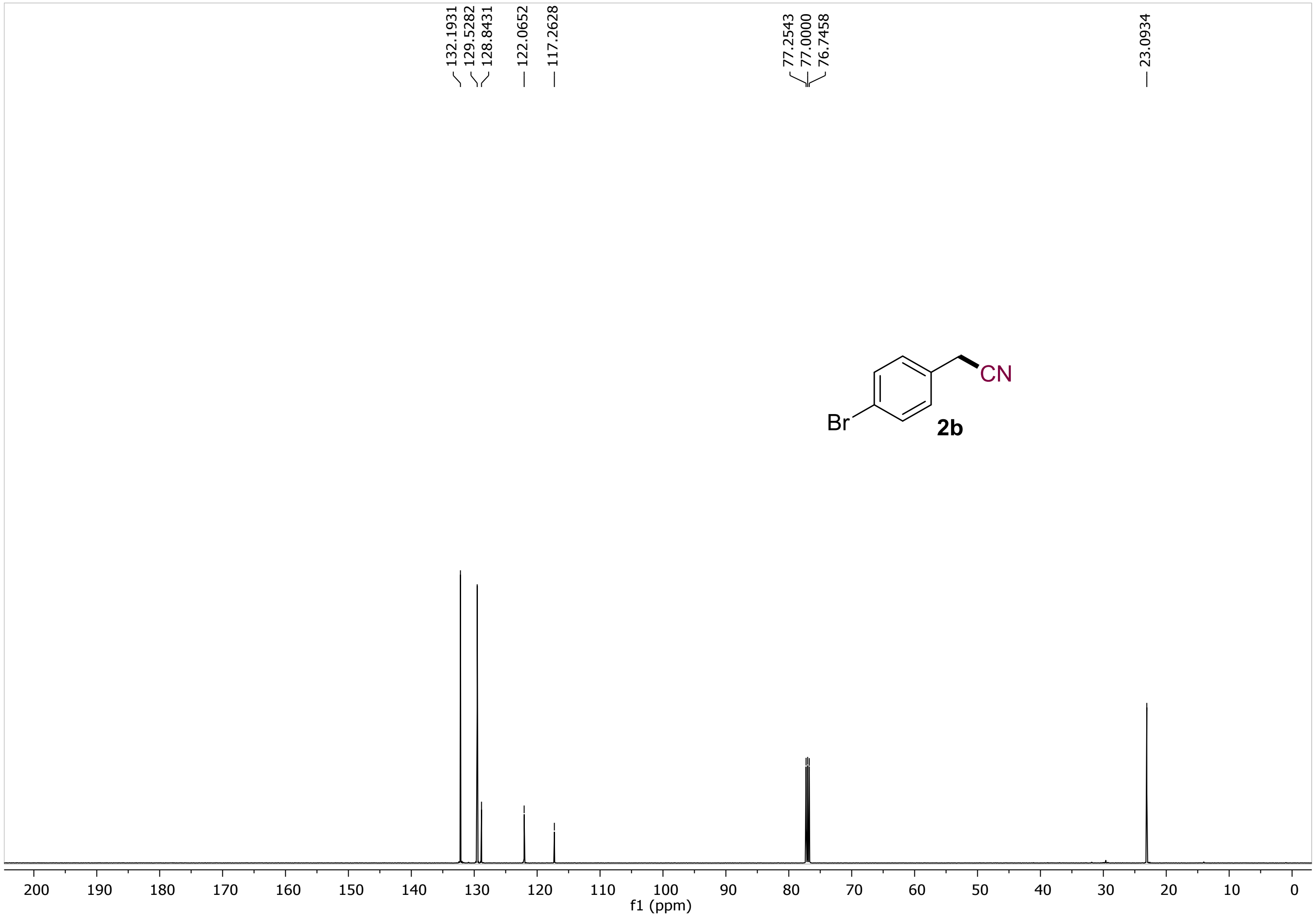

Figure S25. ^{13}C NMR (125 MHz, $CDCl_3$) of the compound **4b.**

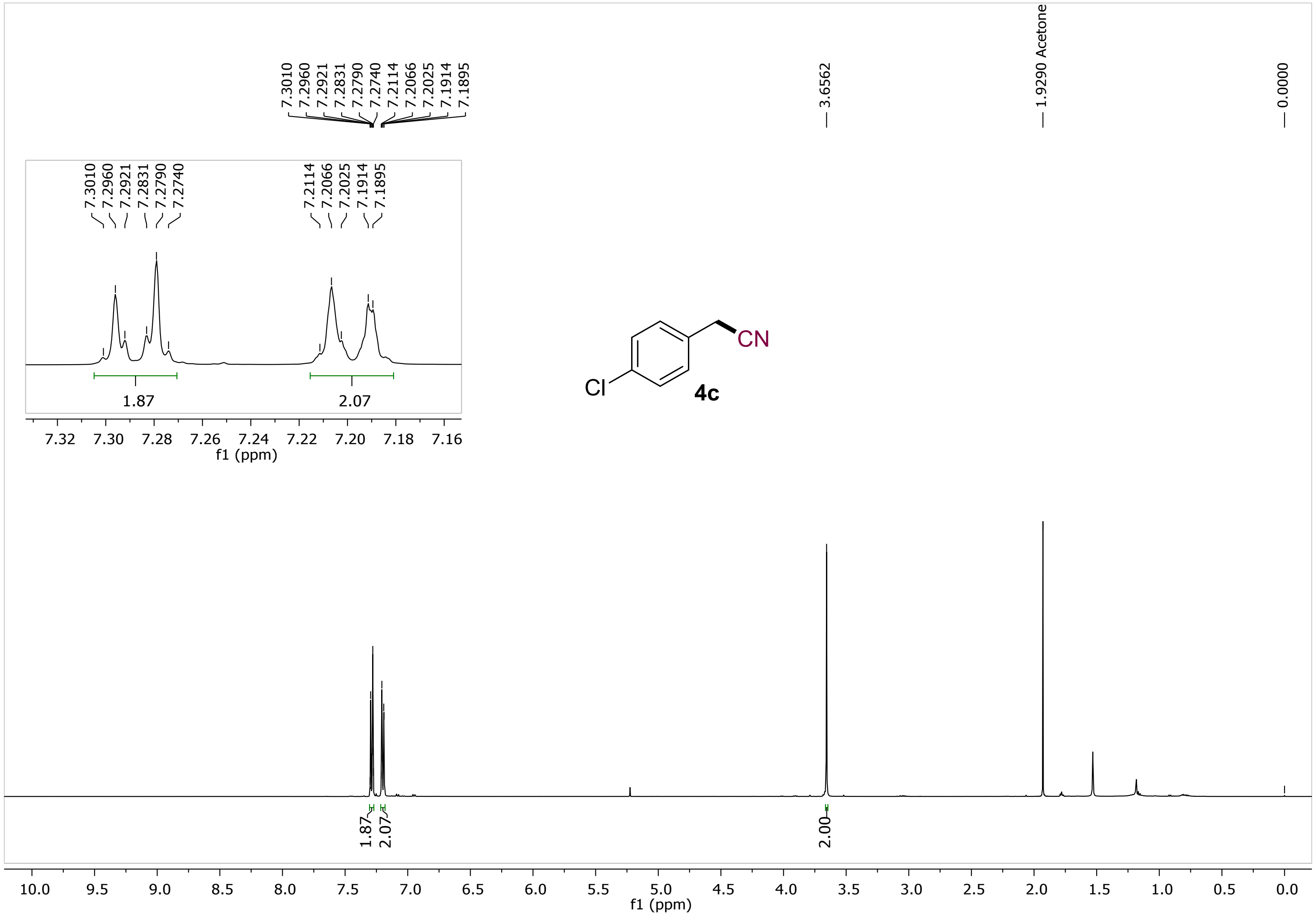

Figure S26. ^{1}H NMR (500 MHz, $CDCl_3$) of the compound **4c.**

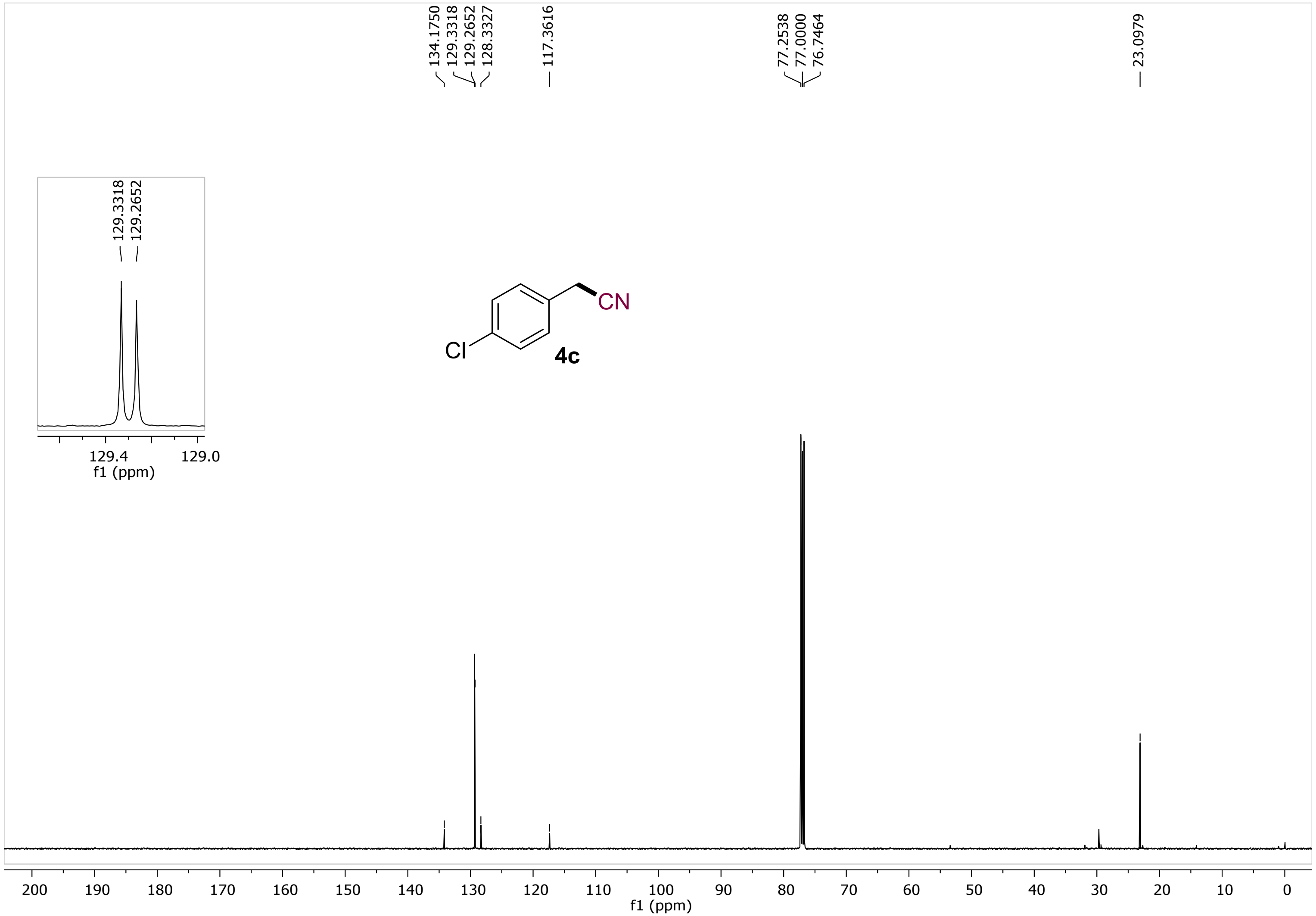

Figure S27. ^{13}C NMR (125 MHz, $CDCl_3$) of the compound **4c.**

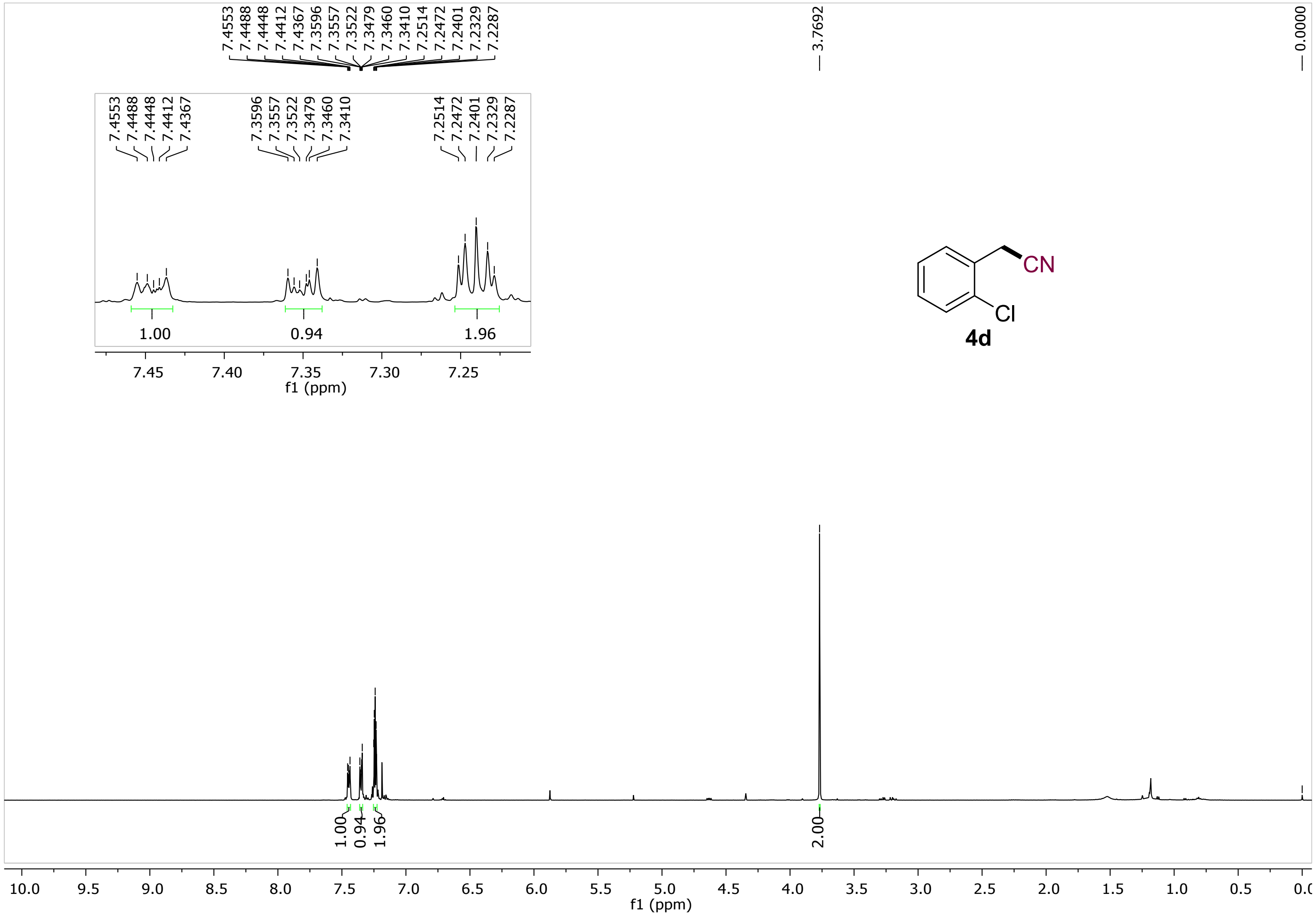

Figure S28. ^{1}H NMR (500 MHz, $CDCl_3$) of the compound **4d.**

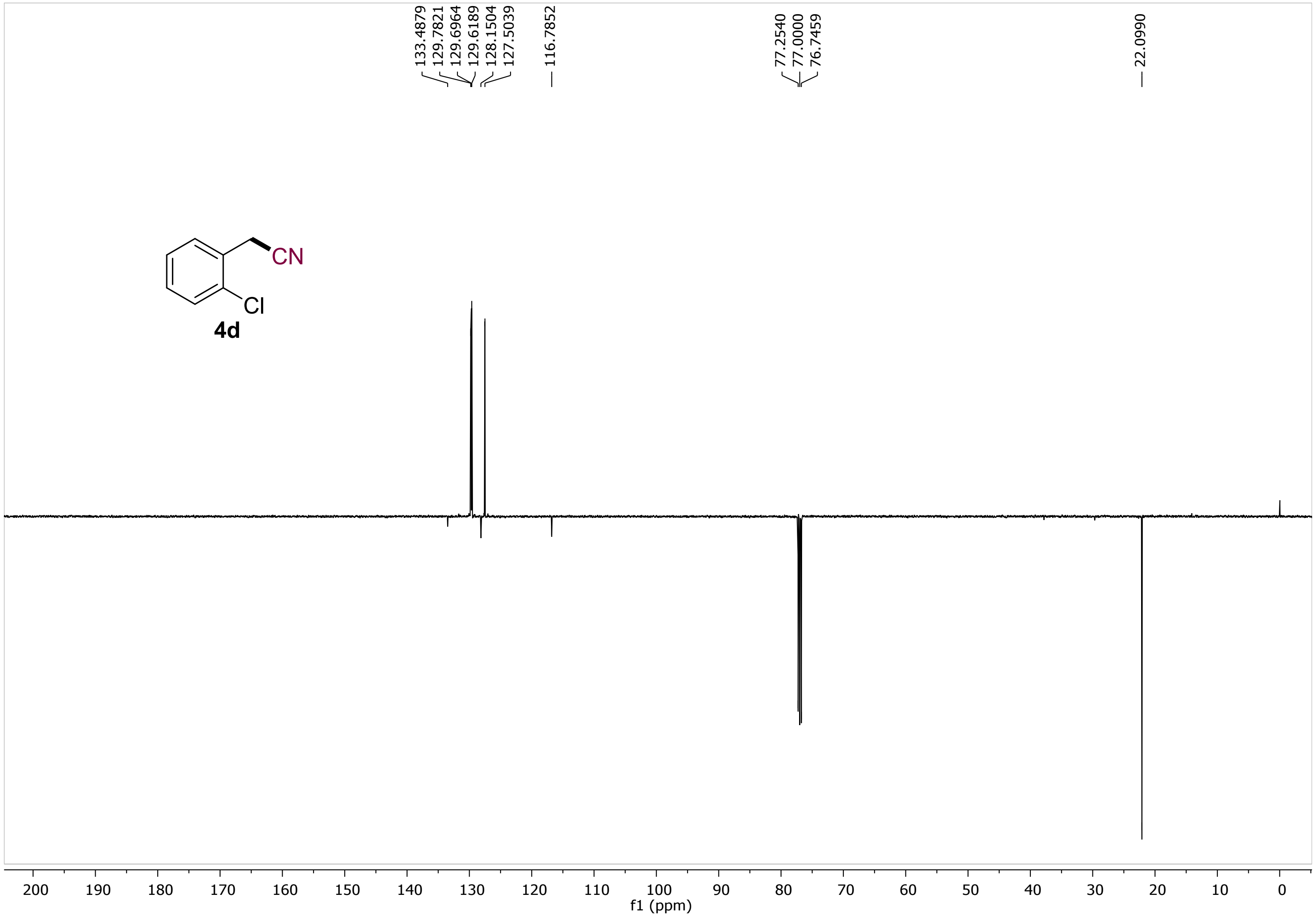

Figure S29. $^{13}C\{^{1}H\}$-APT NMR (125 MHz, $CDCl_3$) of the compound **4d.**

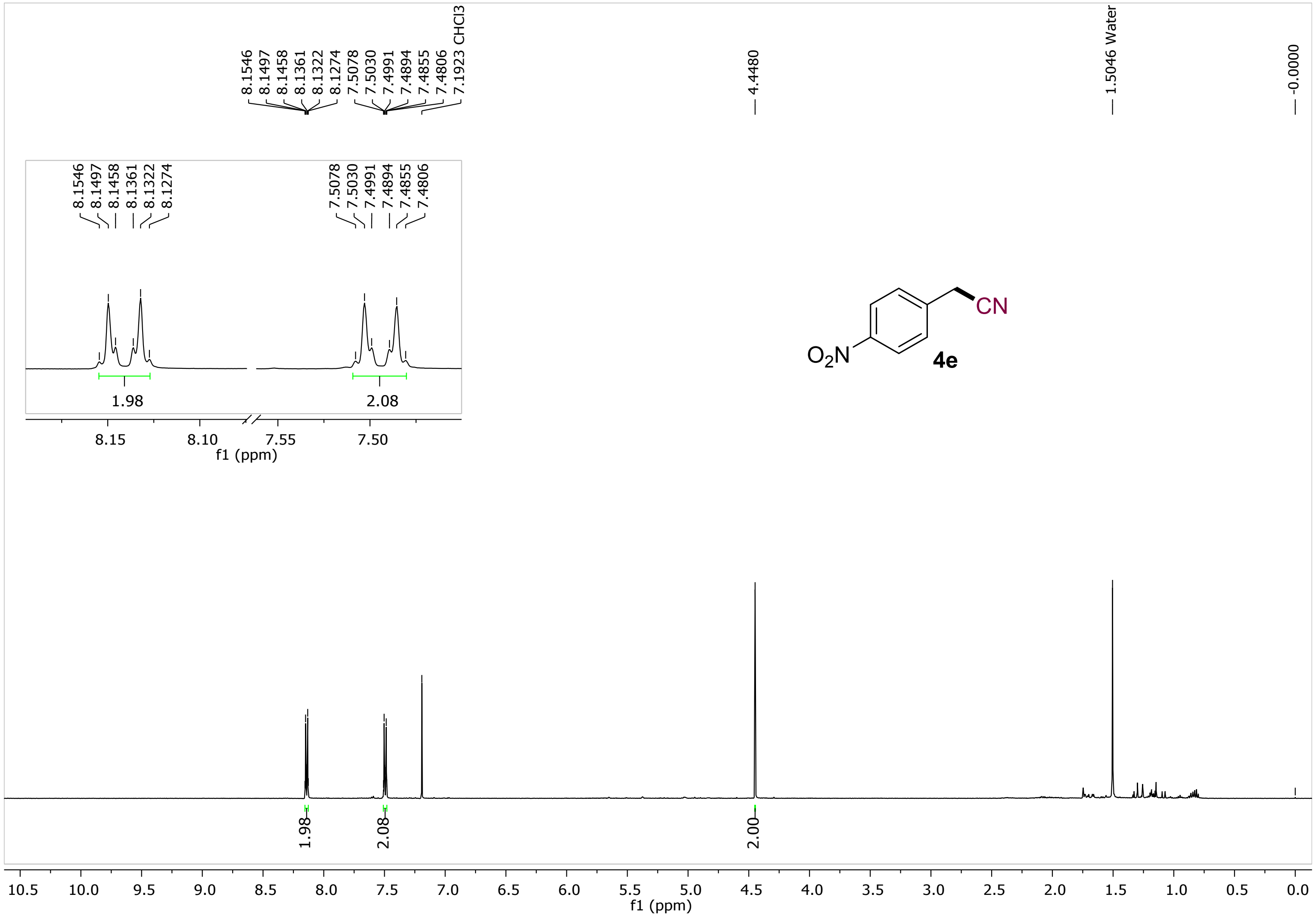

Figure S30. ^{1}H NMR (500 MHz, $CDCl_3$) of the compound **4e.**

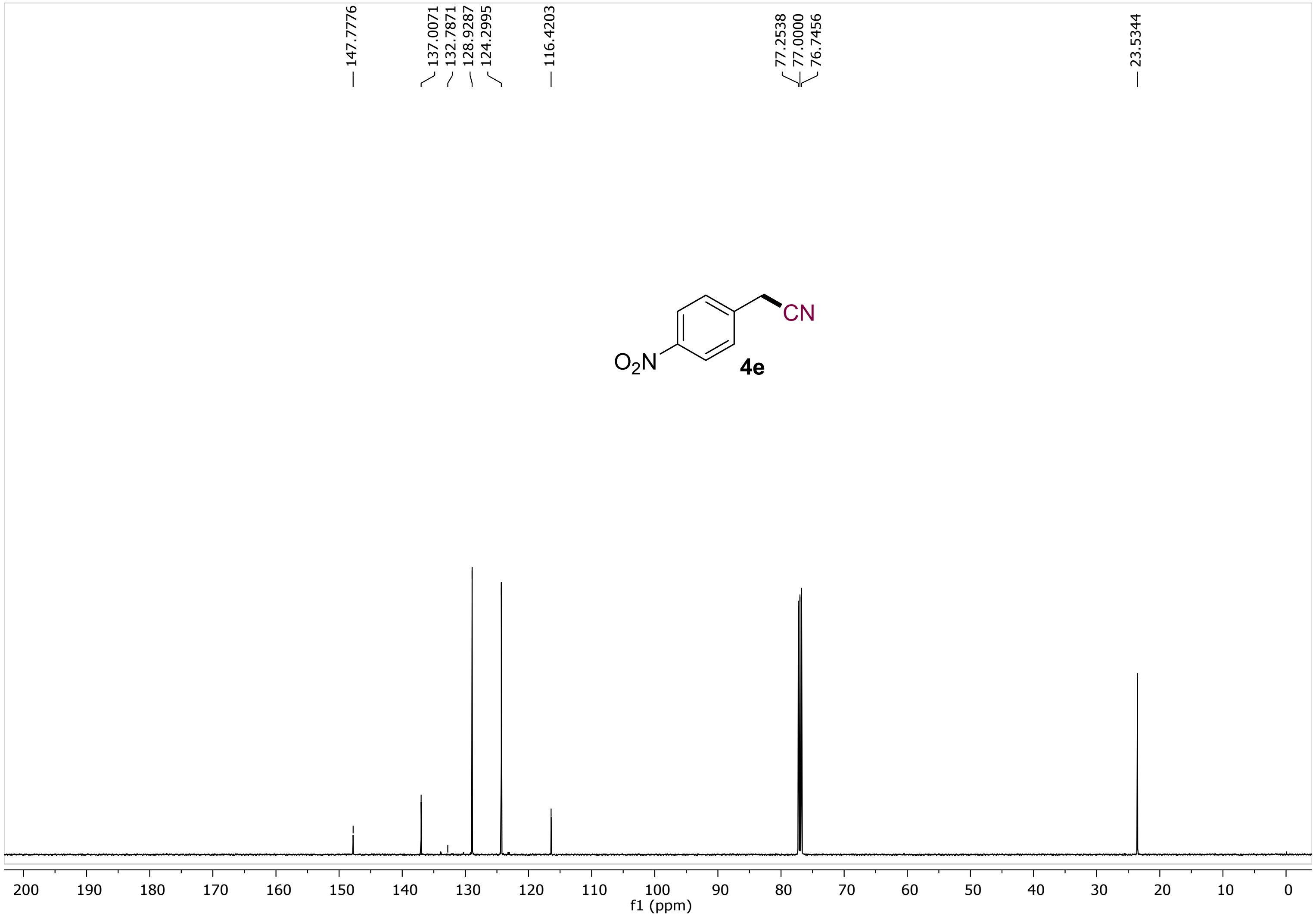

Figure S31. ^{1}H NMR (500 MHz, $CDCl_3$) of the compound **4e.**

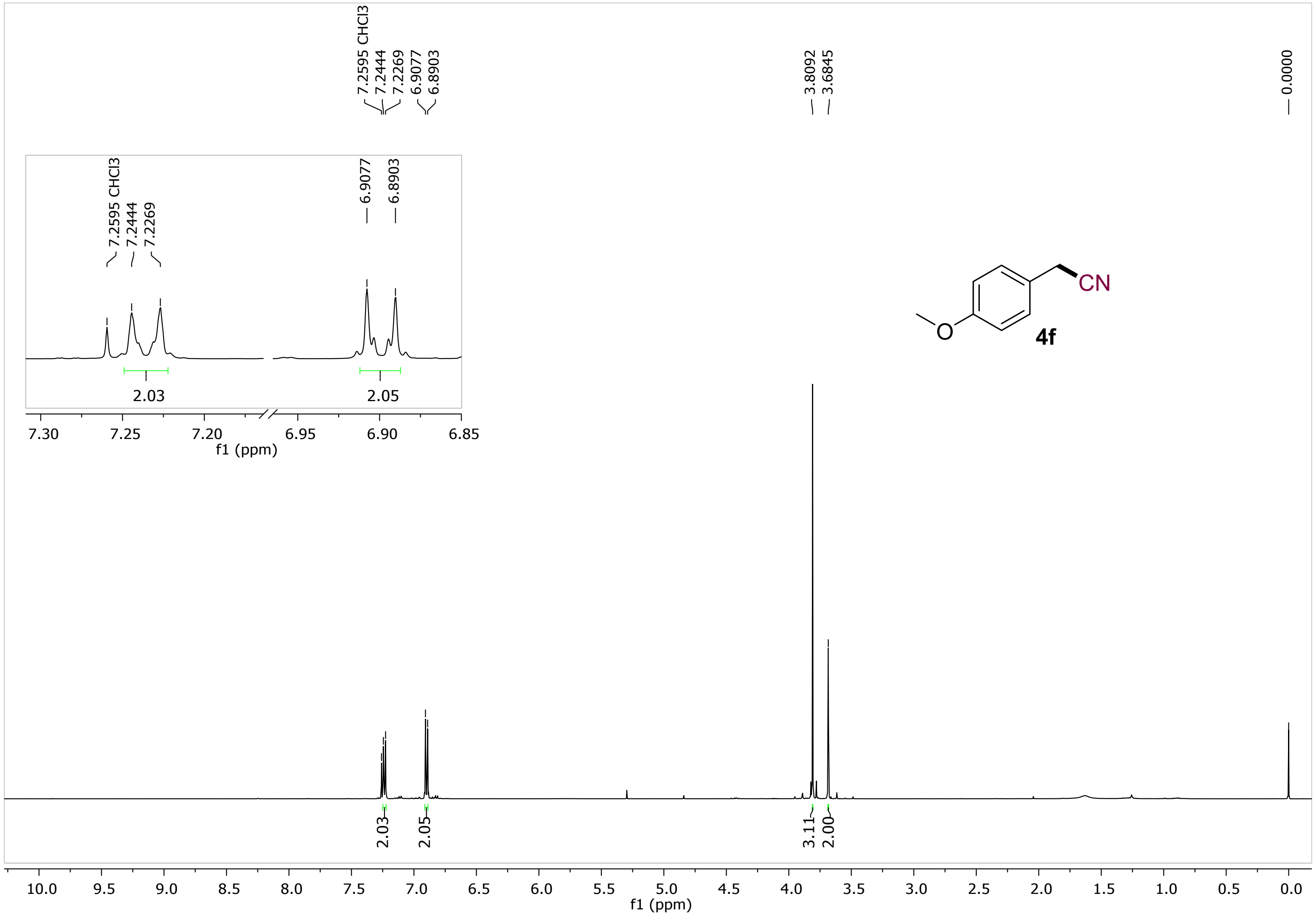

Figure S32. ^{1}H NMR (500 MHz, $CDCl_3$) of the compound **4f.**

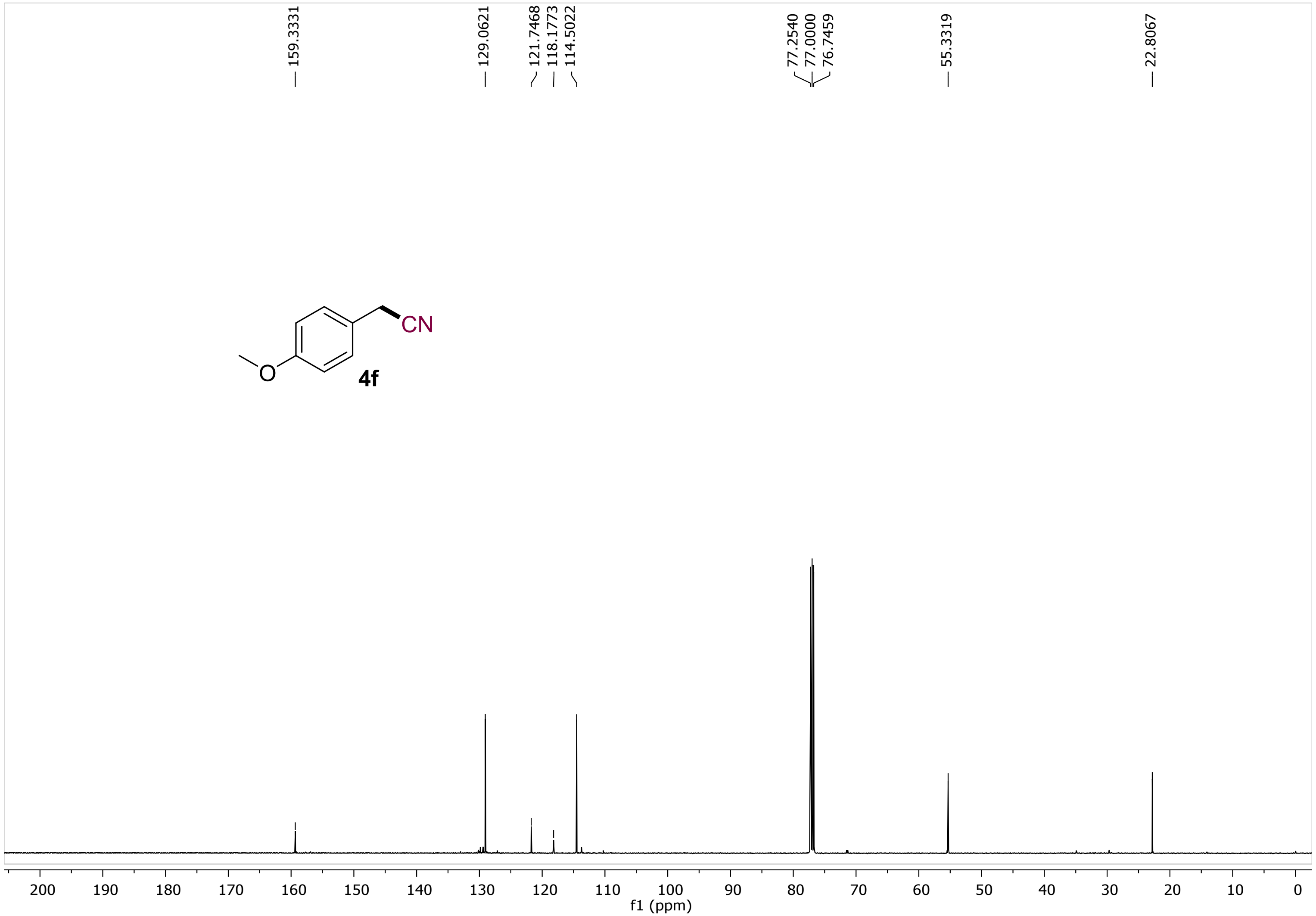

Figure S33. ^{13}C NMR (125 MHz, $CDCl_3$) of the compound **4f.**

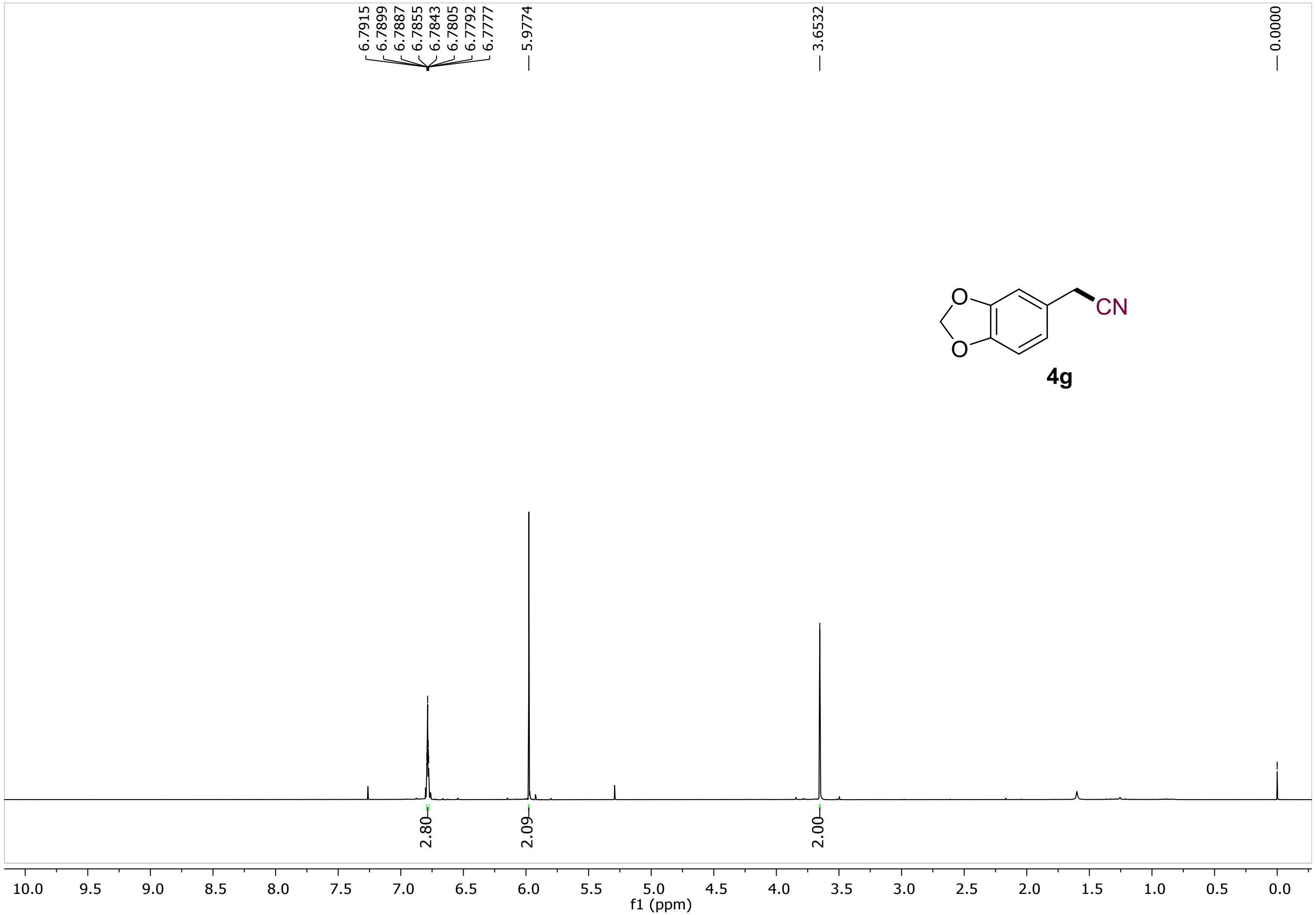

Figure S34. ^{1}H NMR (500 MHz, $CDCl_3$) of the compound **4g.**

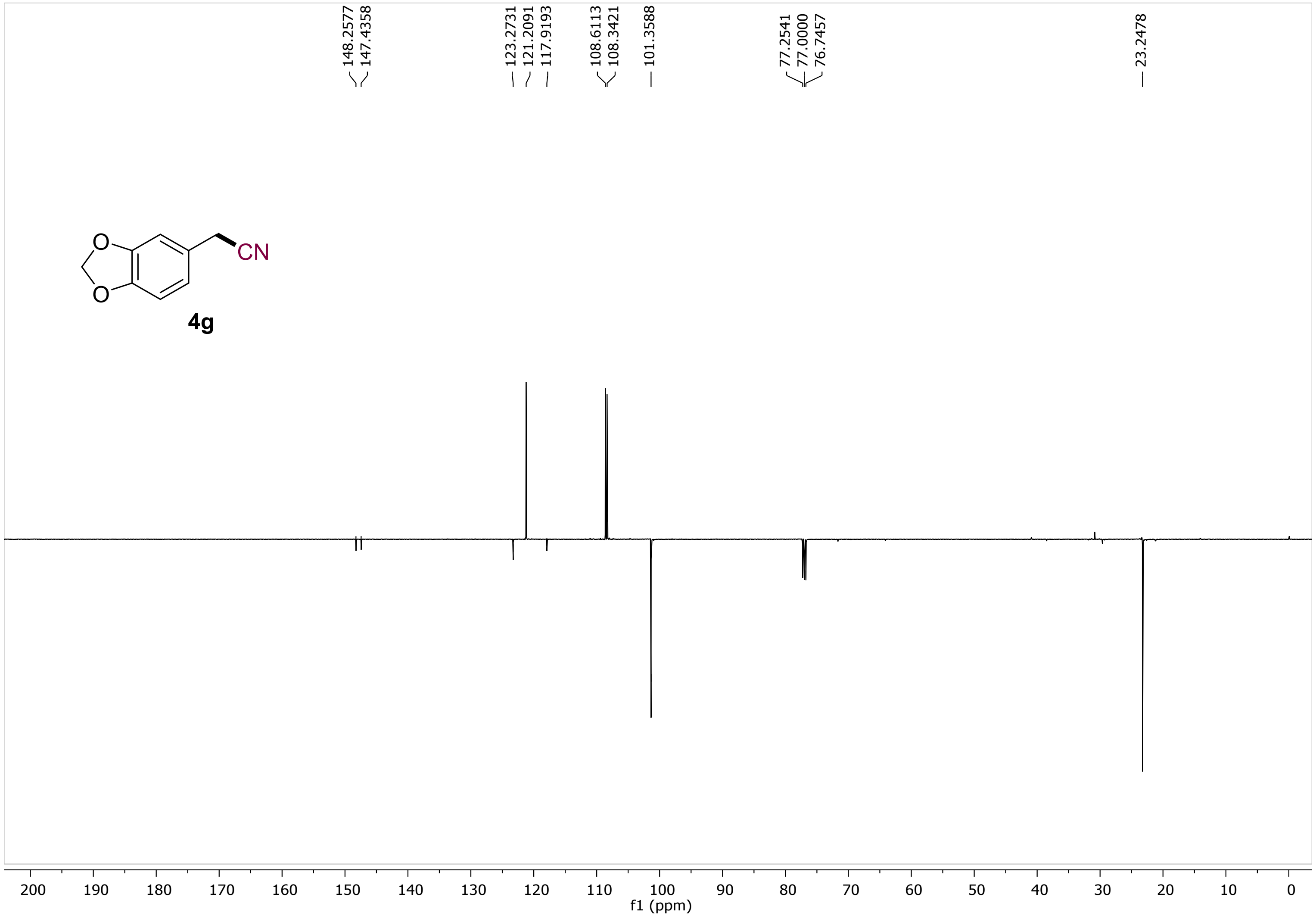

Figure S35. $^{13}C\{^{1}H\}$-APT NMR (125 MHz, $CDCl_3$) of the compound **4g.**

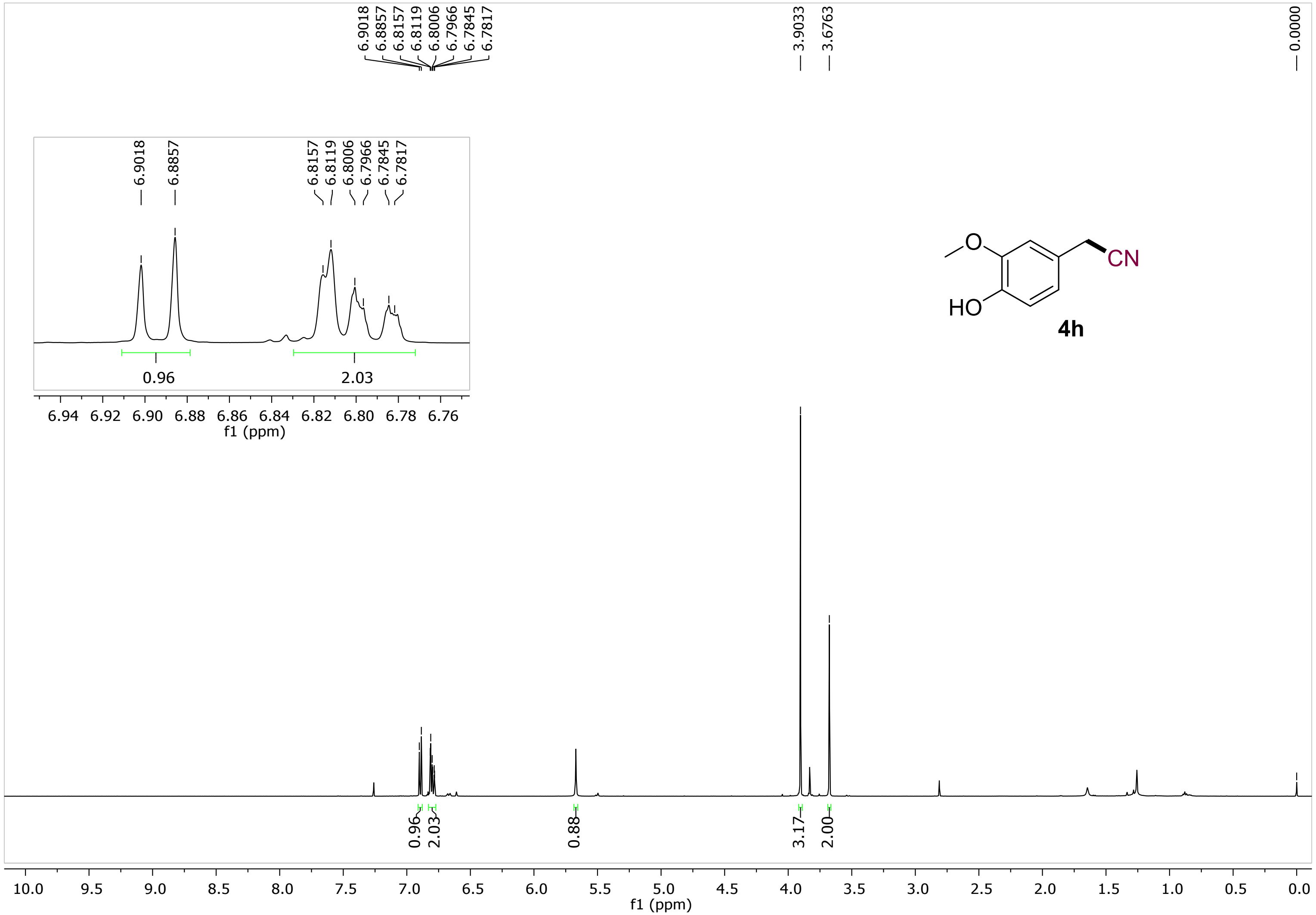

Figure S36. ^{1}H NMR (500 MHz, $CDCl_3$) of the compound **4h.**

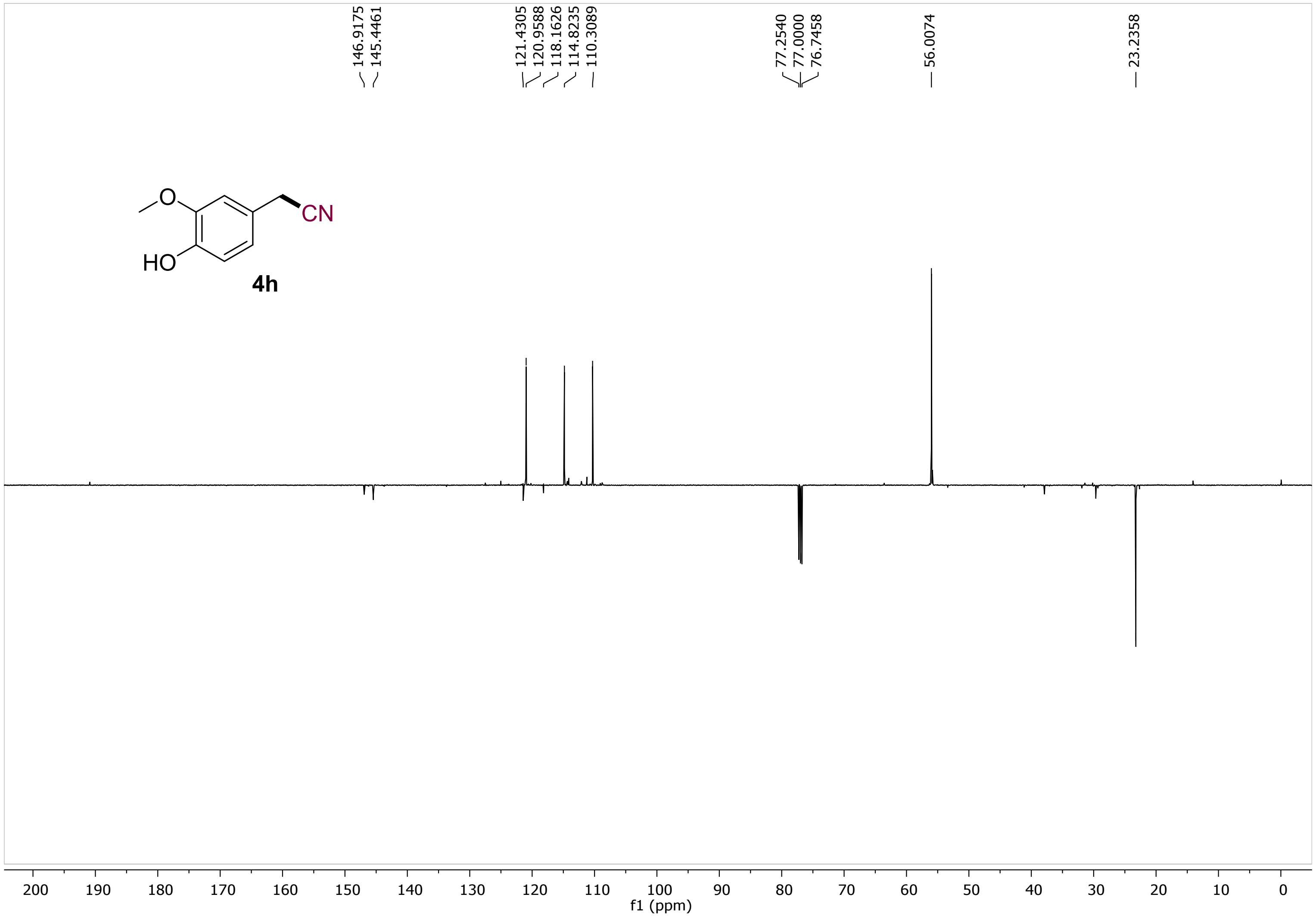

Figure S37. $^{13}C\{^{1}H\}$-APT NMR (125 MHz, $CDCl_3$) of the compound **4h.**

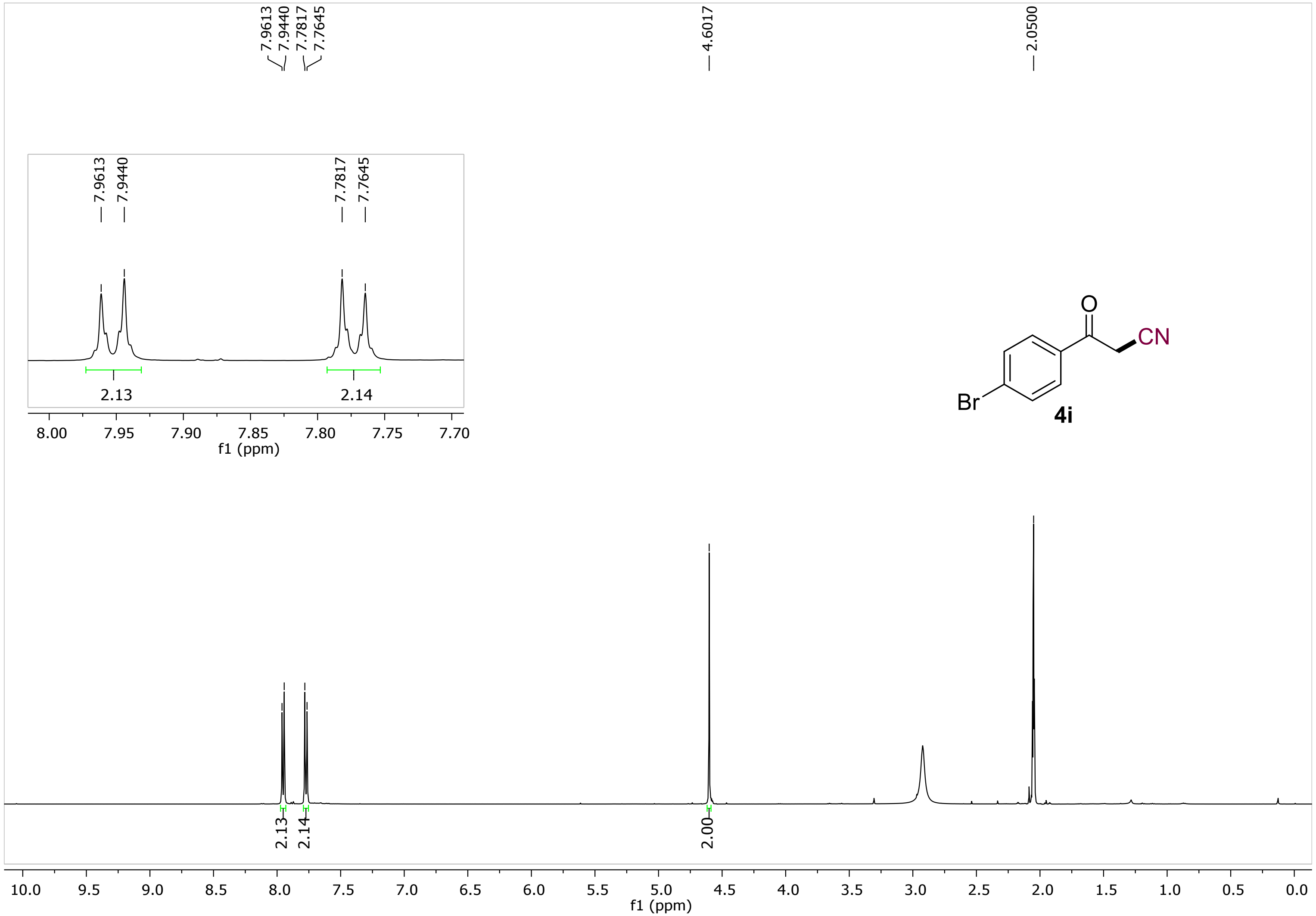

Figure S38. ^{1}H NMR (500 MHz, Acetone-d_6) of the compound **4i.**

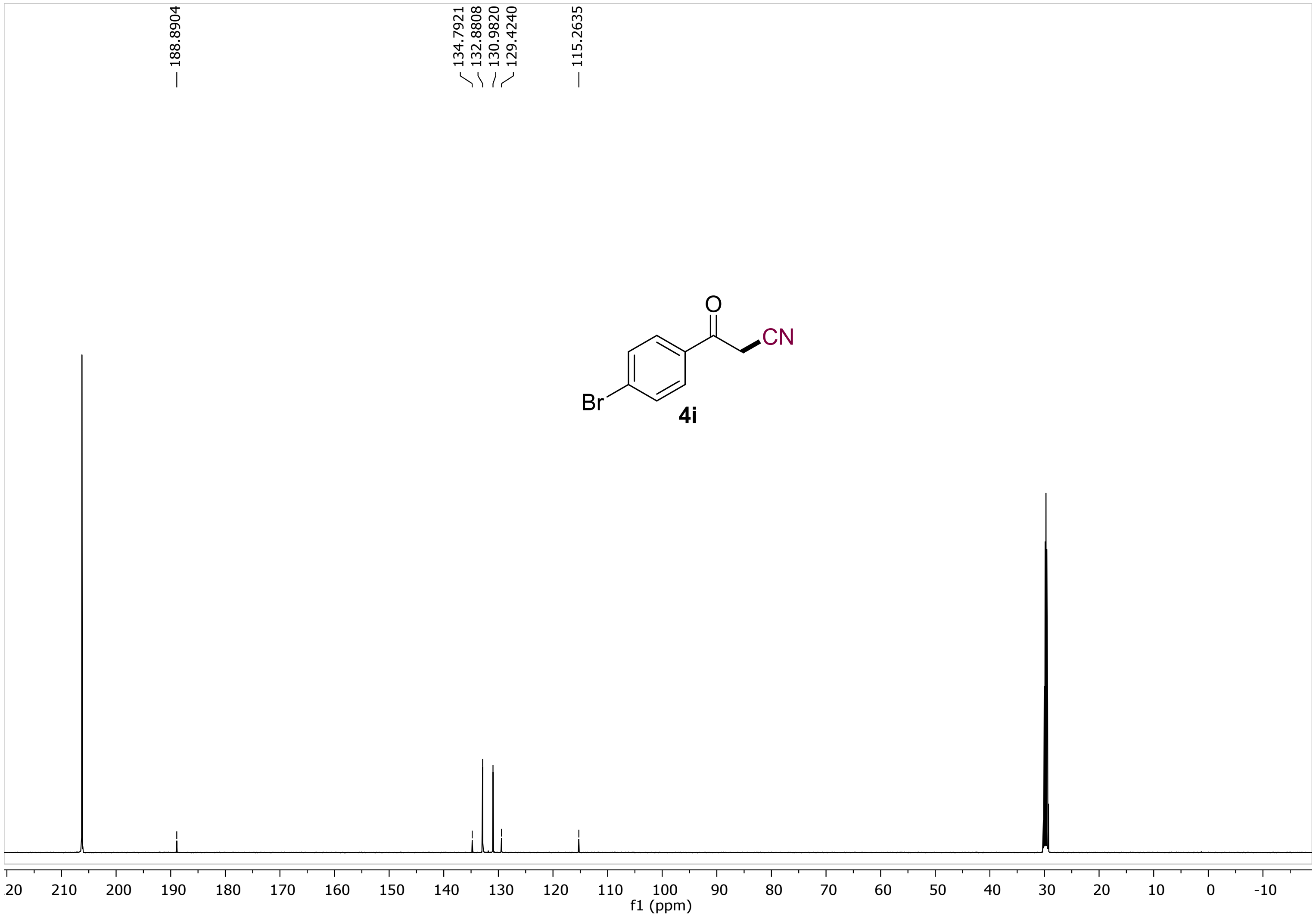

Figure S39. ^{13}C NMR (125 MHz, Acetone-d_6) of the compound **4i.**

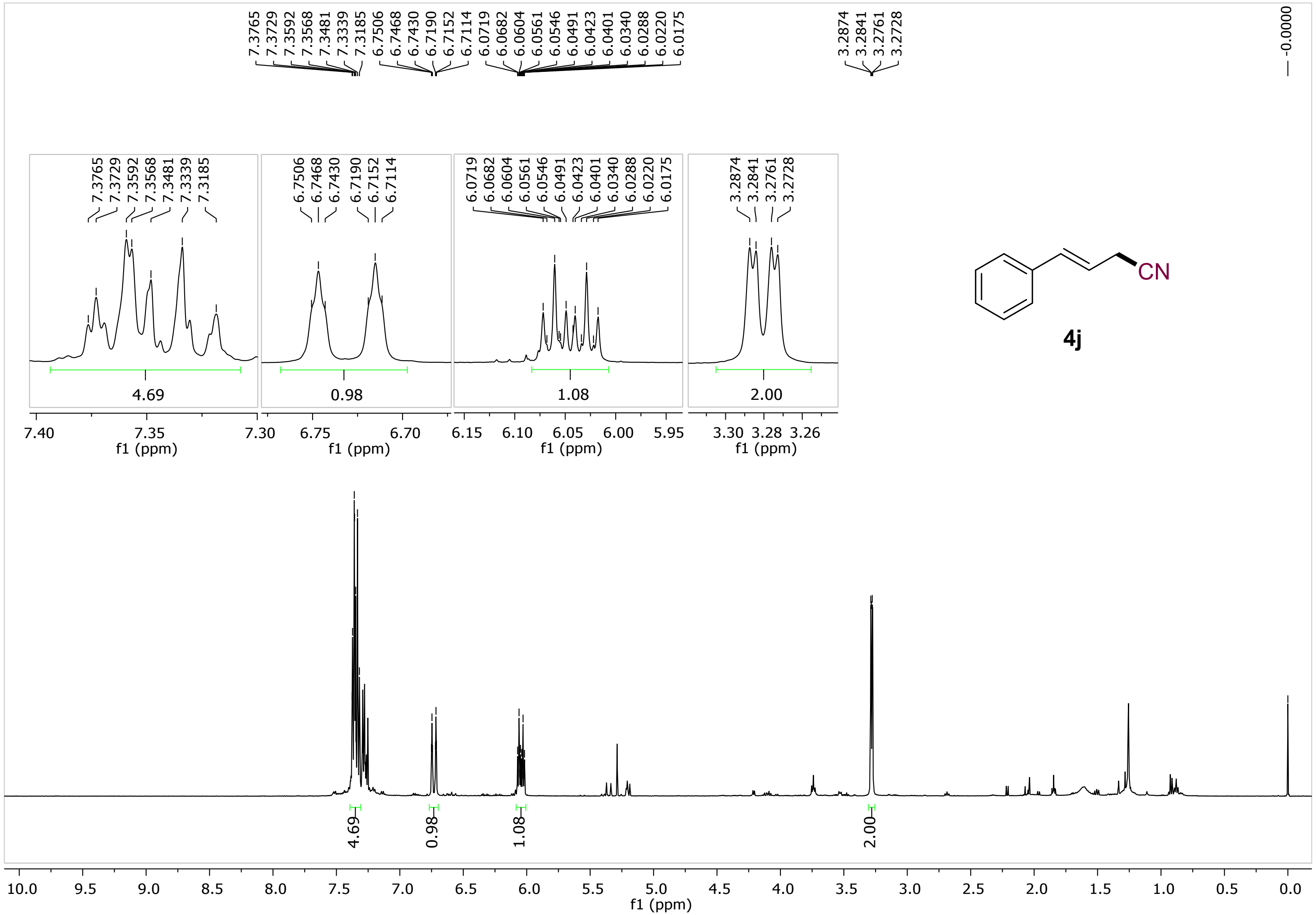

Figure S40. ^{1}H NMR (500 MHz, $CDCl_3$) of the compound **4j.**

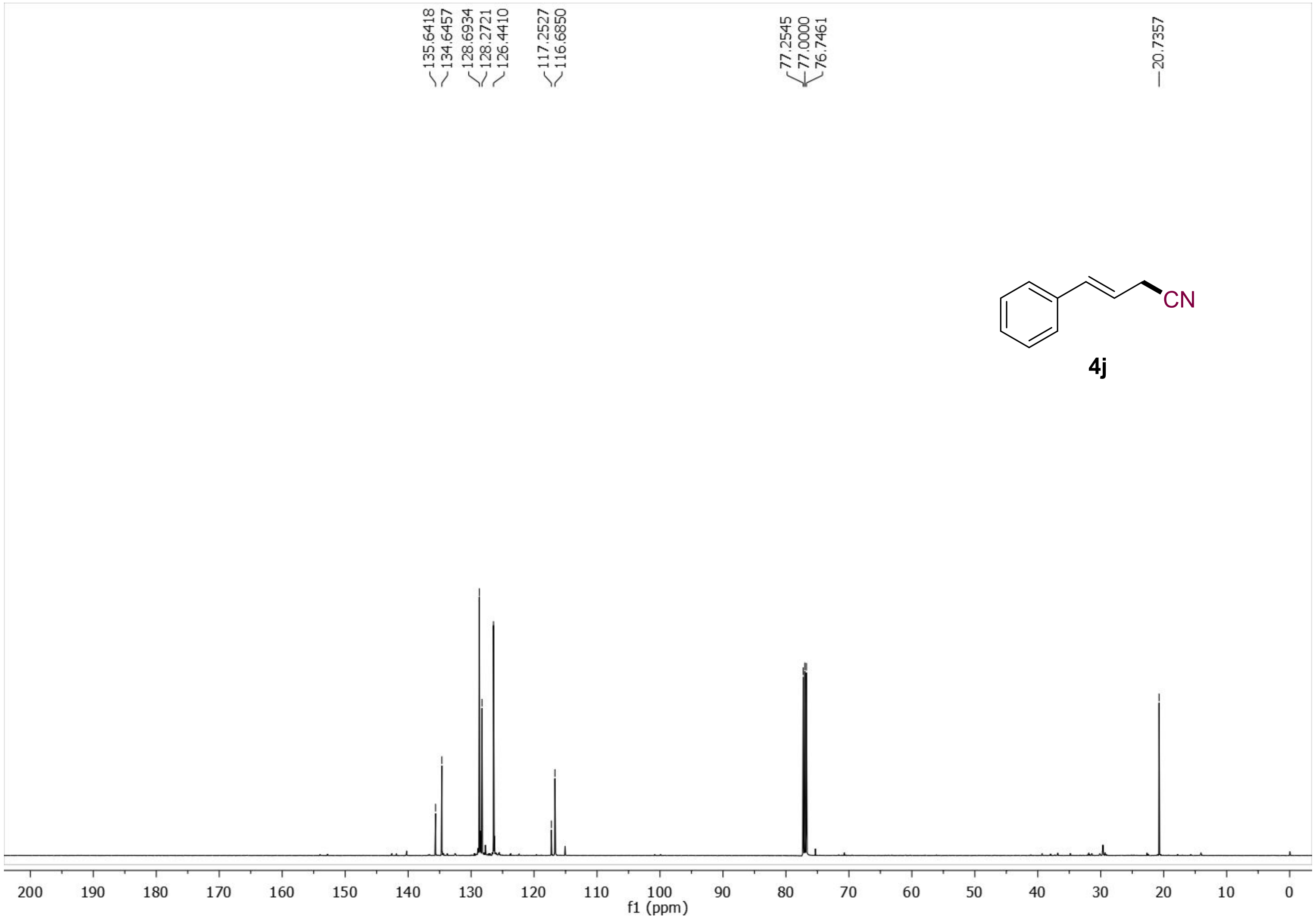

Figure S41. ^{13}C NMR (125 MHz, $CDCl_3$) of the compound **4j.**

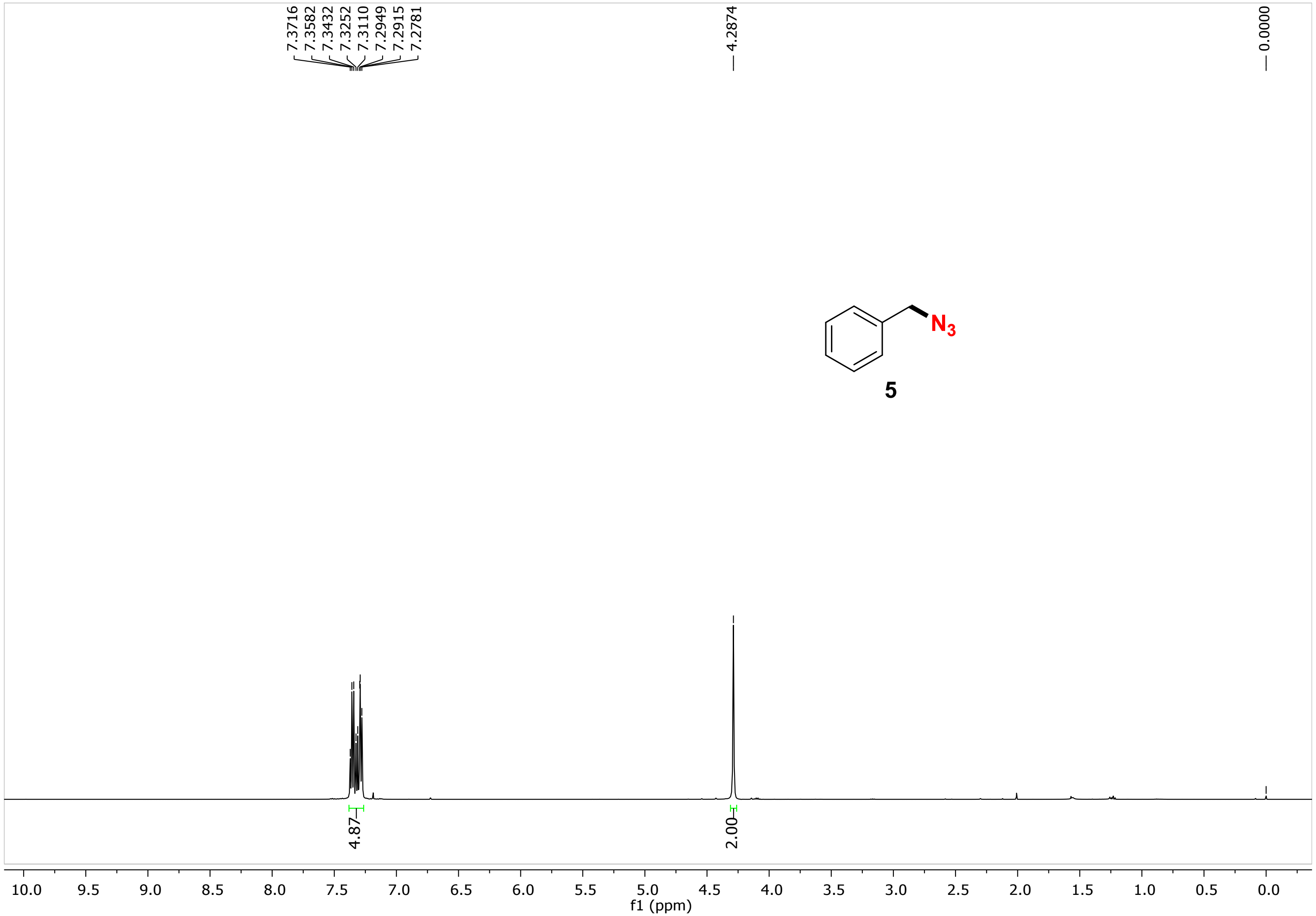

Figure S42. ^{1}H NMR (500 MHz, $CDCl_3$) of the compound **5.**

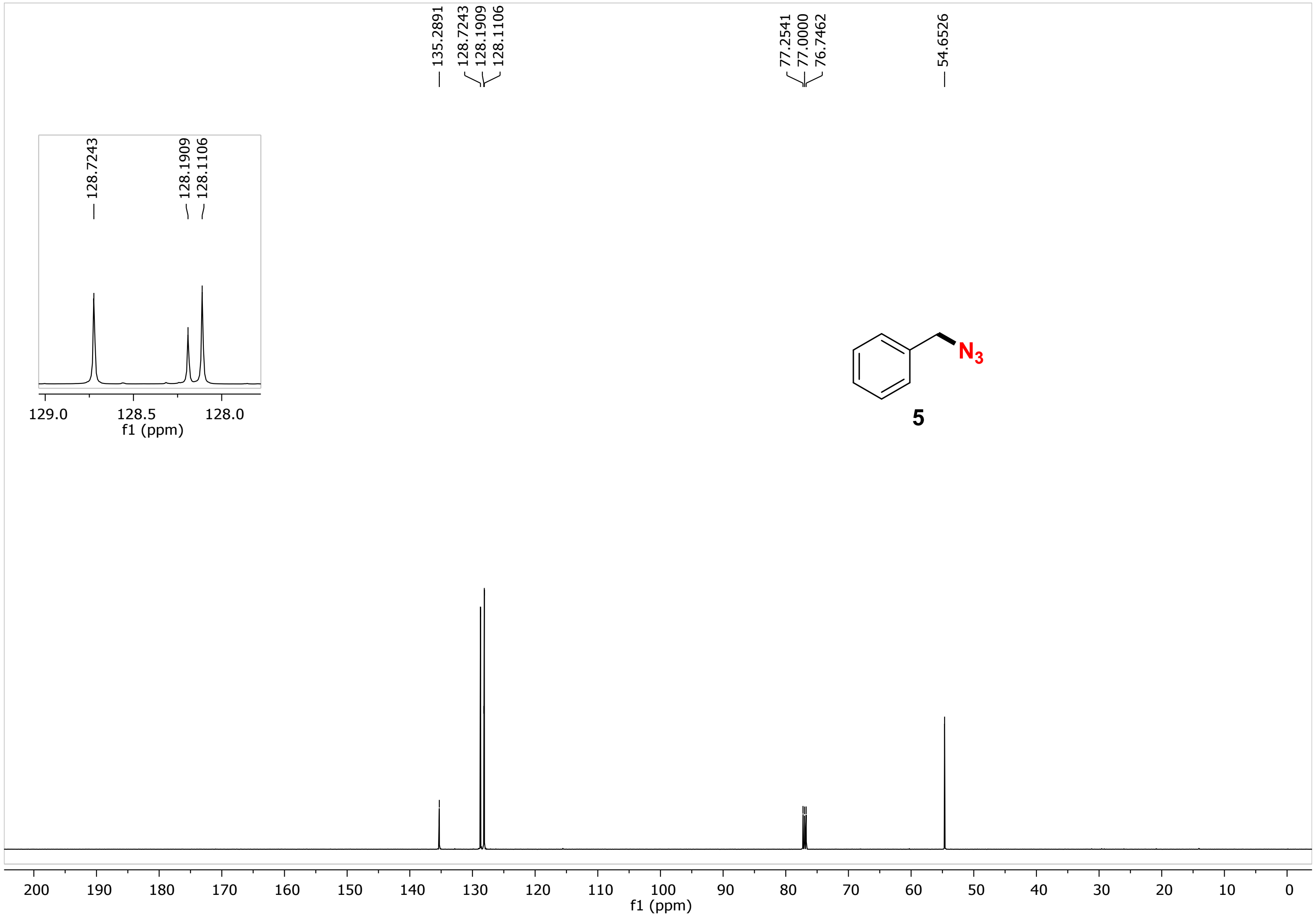

Figure S43. ^{13}C NMR (125 MHz, $CDCl_3$) of the compound **5.**
